# Conformational entropy tuning in nonfused-ring electron acceptors for low-cost and record-efficiency organic solar cells

**DOI:** 10.1093/nsr/nwag177

**Published:** 2026-03-19

**Authors:** Xiaobin Gu, Rui Zeng, Fei Han, Jing Li, Senke Tan, Jikai Lv, Na Yu, Lixuan Kan, Hao Li, Yuqi Hou, Yunhao Cai, Zhixiang Wei, Zheng Tang, Lianrui Hu, Feng Liu, Xin Zhang, Hui Huang

**Affiliations:** College of Materials Science and Opto-Electronic Technology, Center of Materials Science and Optoelectronics Engineering, University of Chinese Academy of Sciences, Beijing 101408, China; School of Chemistry and Chemical Engineering, Frontiers Science Center for Transformative Molecules, In-situ Center for Physical Science, and Center of Hydrogen Science, Shanghai Jiao Tong University, Shanghai 200240, China; School of Chemistry and Chemical Engineering, Frontiers Science Center for Transformative Molecules, In-situ Center for Physical Science, and Center of Hydrogen Science, Shanghai Jiao Tong University, Shanghai 200240, China; Shanghai Engineering Research Center of Molecular Therapeutics and New Drug Development, Shanghai Frontiers Science Center of Molecule Intelligent Syntheses, School of Chemistry and Molecular Engineering, East China Normal University, Shanghai 200241, China; School of Chemistry and Chemical Engineering, Frontiers Science Center for Transformative Molecules, In-situ Center for Physical Science, and Center of Hydrogen Science, Shanghai Jiao Tong University, Shanghai 200240, China; College of Materials Science and Opto-Electronic Technology, Center of Materials Science and Optoelectronics Engineering, University of Chinese Academy of Sciences, Beijing 101408, China; Center for Advanced Low-Dimension Materials, State Key Laboratory for Modification of Chemical Fibers and Polymer Materials, College of Materials Science and Engineering, Donghua University, Shanghai 201620, China; School of Chemistry and Chemical Engineering, Frontiers Science Center for Transformative Molecules, In-situ Center for Physical Science, and Center of Hydrogen Science, Shanghai Jiao Tong University, Shanghai 200240, China; School of Chemistry and Chemical Engineering, Frontiers Science Center for Transformative Molecules, In-situ Center for Physical Science, and Center of Hydrogen Science, Shanghai Jiao Tong University, Shanghai 200240, China; College of Materials Science and Opto-Electronic Technology, Center of Materials Science and Optoelectronics Engineering, University of Chinese Academy of Sciences, Beijing 101408, China; College of Materials Science and Opto-Electronic Technology, Center of Materials Science and Optoelectronics Engineering, University of Chinese Academy of Sciences, Beijing 101408, China; Center for Excellence in Nanoscience (CAS), Key Laboratory of Nanosystem and Hierarchical Fabrication (CAS), National Center for Nanoscience and Technology, Beijing 100190, China; Center for Advanced Low-Dimension Materials, State Key Laboratory for Modification of Chemical Fibers and Polymer Materials, College of Materials Science and Engineering, Donghua University, Shanghai 201620, China; Shanghai Engineering Research Center of Molecular Therapeutics and New Drug Development, Shanghai Frontiers Science Center of Molecule Intelligent Syntheses, School of Chemistry and Molecular Engineering, East China Normal University, Shanghai 200241, China; School of Chemistry and Chemical Engineering, Frontiers Science Center for Transformative Molecules, In-situ Center for Physical Science, and Center of Hydrogen Science, Shanghai Jiao Tong University, Shanghai 200240, China; College of Materials Science and Opto-Electronic Technology, Center of Materials Science and Optoelectronics Engineering, University of Chinese Academy of Sciences, Beijing 101408, China; College of Materials Science and Opto-Electronic Technology, Center of Materials Science and Optoelectronics Engineering, University of Chinese Academy of Sciences, Beijing 101408, China; School of Chemical Engineering and Technology, State Key Laboratory of Chemical Engineering and Low-Carbon Technology, Tianjin University, Tianjin 300072, China; The International Joint Institute of Tianjin University, Fuzhou 350205, China

**Keywords:** organic solar cells, highly cost-effective, non-fused-ring electron acceptors, conformational entropy, intramolecular noncovalent interactions

## Abstract

The commercial viability of organic solar cells (OSCs) is hindered by the trade-off between cost and performance. In particular, low-cost non-fused-ring electron acceptors (NFREAs) suffer from conformational disorder, limiting their photovoltaic performance. Herein, we strategically regulate conformational entropy (*S*_conf._) of NFREAs through the rational combination of intramolecular noncovalent interactions (INIs). The optimal candidate 3TT-SeS, identified through comprehensive density functional theory calculations, exhibits balanced Se···N and S···O INIs and demonstrates a significant reduction in *S*_conf_. This strategy enables an exclusive single stable conformation and highly ordered molecular packing, thereby facilitating efficient charge transport. Consequently, the 3TT-SeS-based OSC achieved an outstanding power conversion efficiency of 19.26% (certified at 18.75%), setting a new benchmark for NFREA-based systems to date. More importantly, 3TT-SeS-based device demonstrates exceptional economic potential with an extremely low power generation cost of 0.77$ kW^−1^, much lower than several high-performance FREA-based systems. Our work demonstrates a low-*S*_conf._ design of NFREAs for cost-effective and high-performance organic photovoltaics.

## Teaser

Forget the ‘cost vs. efficiency’ trade-off: our low-cost organic photovoltaic materials broke the 19% efficiency barrier, setting a new benchmark for affordable clean energy.

## INTRODUCTION

**Figure 1.**
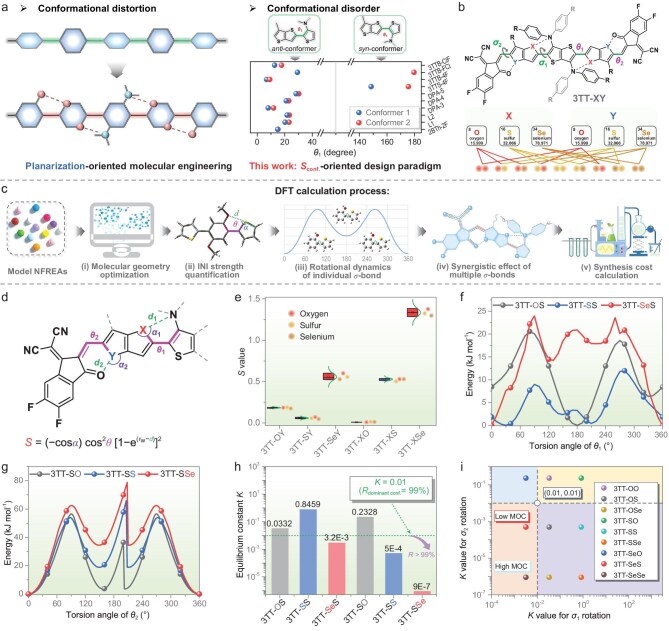
Comprehensive DFT calculations. (a) Fundamental challenges posed by rotatable *σ*-bonds. (b) Design strategy for model NFREAs. (c) Flowchart of DFT calculation procedures. (d) Geometric parameters and formula for calculating *S*_INI_ values: *r*_w_ is the sum of van der Waals radii of two involved atoms; Δ*d* is defined as *r*_w_–*d*. (e) *S*_INI_ values under different chalcogen substitutions (Each data point represents the computed *S*_INI_ value for a specific INI site (X, Y = O, S, or Se) within a candidate NFREA. Refer to Table S3 for detailed values). (f and g) PES scan curves. (h) Equilibrium constants between stable and metastable states. (i) Four-quadrant diagram of two *K* values and MOC index.

Organic solar cells (OSCs) have demonstrated great potential for multi-scenario applications due to their unique advantages, including solution processability, mechanical flexibility, and tunable transparency [1–3]. Remarkable progress in both material design and device engineering [4–6], particularly through the groundbreaking invention of fused-ring electron acceptors (FREAs) [7–9], has propelled the power conversion efficiencies (PCEs) of OSCs beyond 20% [10–16]. Despite these achievements, the field faces a long-standing cost-efficiency paradox that substantially hinders large-scale commercialization due to the complex molecular structures and costly synthetic processes required for FREAs [17,18]. In response, non-fused-ring electron acceptors (NFREAs) have emerged as promising candidates for low-cost OSCs. NFREAs feature simple-structured and *σ*-bond-connected conjugated backbones that enable facile and scalable synthesis [19–28], yet their structural flexibility presents two fundamental challenges: (i) conformational distortion (large torsion angles) and (ii) conformational disorder (coexistence of several conformers) (Fig. 1a and Fig. S1, Table S1). Current research has predominantly addressed the first challenge through planarization-oriented molecular engineering, achieving improved backbone coplanarity [29–34]. Nevertheless, the equally critical issue of conformational disorder has been largely neglected, highlighting the urgent need for innovative design paradigm in developing conformer-free NFREAs.

Conformational entropy (*S*_conf._), a fundamental thermodynamic parameter quantifying the extent of conformational disorder within molecular systems, holds a pivotal position in both chemistry and biology [35–38]. Essentially, the Boltzmann entropy formula (*S*_conf._ = *k*_B_ln*W*, where *W* denotes the number of accessible conformations [39]) establishes that reducing *S*_conf._ directly correlates with decreased conformational disorder. This principle finds remarkable parallels between biological macromolecular folding and the aggregated structural ordering of organic semiconductors [40,41]. During polypeptide-to-protein folding, the significant reduction in *S*_conf._ is essential for forming structurally defined assemblies with biological functionality [42–44]. Analogously, suppressing conformational disorder in organic semiconductors is crucial for achieving the highly ordered molecular packing and efficient charge transport required for high-performance organic optoelectronic devices [45–47]. Therefore, establishing a rational design paradigm to reduce *S*_conf._ could unlock the full potential of NFREA in cost-effective OSC applications.

In this contribution, we proposed a low-*S*_conf._ design of NFREAs and systematically investigated its influence on molecular self-assembly and photovoltaic performance. Through comprehensive density functional theory (DFT) calculations on NFREAs with varied intramolecular noncovalent interactions (INIs), we identified molecules exhibiting both low-*S*_conf._ and cost-effectiveness. Subsequently, we successfully synthesized the top-two candidates (3TT-SeS and 3TT-SSe), and conducted comprehensive theoretical and experimental analyses. The results revealed that 3TT-SeS, featuring balanced INIs, demonstrated a significant *S*_conf._ reduction, exhibiting an exclusive single conformation, highly ordered self-assembled structure, and favorable blend-film morphology. Thus, 3TT-SeS based OSCs achieved a record-high value of 19.26% (certified at 18.75%) for low-cost NFREA-based ones. Impressively, the 3TT-SeS based devices achieved a substantially lower power generation cost (0.77$ kW^−1^) than the state-of-the-art FREA-based ones, such as D18-Cl: BTP-C3F (3.69$ kW^−1^ at 20.80% PCE).

## RESULTS AND DISCUSSION

### Comprehensive DFT calculations

Developing NFREAs with low-*S*_conf._ is challenging, primarily due to the intricate rotational dynamics associated with multiple *σ*-bonds. To overcome this, we performed comprehensive DFT calculations based on model molecules, which offer a promising alternative to empirical trial-and-error methods for guiding the synthetic prioritization of novel materials [48,49]. Our strategy involves the incorporation of chalcogens (oxygen, sulfur, or selenium) into the design of model NFREAs, denoted as 3TT-XY (X, Y=O, S, or Se) (Fig. S2). This design enables the construction of various possible INIs, including S···N, Se···N, S···O, and Se···O (Fig. 1b). This screening process encompasses five key steps (Fig. 1c): (i) optimizing molecular geometry using DFT calculations; (ii) extracting geometric parameters to quantify the INI strength using an established descriptor (*S*) [50]; (iii) analyzing the rotational dynamics of individual *σ*-bonds; (iv) assessing synergistic effects among multiple *σ*-bonds; and (v) calculating synthesis costs to ensure alignment with the low-cost design goal.

As illustrated in Fig. S3, the optimized molecular geometries of all model NFREAs exhibit a relatively planar backbone. The extracted geometric parameters are summarized in Table S2. Notably, the torsion angles *θ*_1_ (∼18°) and *θ*_2_ (∼0°) are consistent with statistical data obtained from reported crystals. The intramolecular distances (*d*) observed in these models indicate chalcogen-driven INIs, with atom pairs (O···S: 2.86 Å, S···N: 3.12 Å, and Se···N: 2.93 Å in 3TT-XS; O···O: 2.70 Å, S···O: 2.71 Å, and Se···O: 2.63 Å in 3TT-SY) exhibiting substantial contraction compared to their van der Waals radii sums (*r*_w_) (Table S3). To quantify the strength of these INIs, the *S*_INI_ values are calculated and found to range between 0.178 and 0.188, 0.052–0.069, 0.525–0.601, 0.0096–0.0097, 0.513–0.537, and 1.287–1.385, respectively (Fig. 1d–e, Table S3). Note that a larger *S*_INI_ value implies a stronger INI, which is expected to stabilize the molecular conformation [50].

Due to the minimal fluctuation in each *S*_INI_ value, 3TT-XS and 3TT-SY were selected as representatives to investigate the rotational dynamics of single bonds *σ*_1_ and *σ*_2_, respectively, through relaxed potential energy surface (PES) scans (Fig. S4 and Fig. 1f–g). For *σ*_1_ in the 3TT-XS series, when X is oxygen, the stable *syn*-conformation has a torsion angle *θ*_1_ of 180°. In contrast, when X is sulfur or selenium, the preferred *anti*-conformation exhibits a torsion angle *θ*_1_ of 30° and 0°. It is worth noting that the steepness of the potential energy curves can be quantified by their first derivative, with steeper curves indicating a reduced probability of the coexistence of *anti-*/*anti*- or *syn-*/*syn*- conformers [51]. As shown in Fig. S5, the derivative values at 10° near the energy minima of the PES curves follow the trends of 3TT-SeS > 3TT-SS > 3TT-OS for *σ*_1_ and 3TT-SSe > 3TT-SS > 3TT-SO for *σ*_2_, respectively. This result indicates that stronger INIs (corresponding to larger *S*_INI_ values) not only facilitate planar conformation but also effectively suppress conformer coexistence. In addition, the energy differences (Δ*E*_s-ms_) between the stable and metastable states are 8.442, 0.415, and 14.245 kJ mol^−1^ for 3TT-OS, 3TT-SS, and 3TT-SeS, respectively. For *σ*_2_ in the 3TT-SY series (with chalcogen substitution at the Y position), the torsion angle *θ*_2_ is consistently located at 0° for all candidates. The Δ*E*_s-ms_ are 3.613, 18.858, and 34.440 kJ mol^−1^ for 3TT-SO, 3TT-SS, and 3TT-SSe, respectively. Using the equation *K* = exp(−Δ*E*_s-ms_/*RT*) [52], the equilibrium constant (*K*) for these two *anti-*/*syn*- conformers can be calculated to determine their relative populations under thermodynamic equilibrium, thereby reflecting their stability preferences. A *K* value below 0.01 suggests that the dominant conformer (*R*_dominant conf._) accounts for >99% of the population, thereby implying low *S*_conf._ As shown in Fig. 1h and Table S4, the *K* values of 3TT-OS, 3TT-SS, and 3TT-SeS, based on *σ*_1_ rotation, are 0.0332, 0.8459, and 0.0032, respectively, while those of 3TT-SO, 3TT-SS, and 3TT-SSe, based on *σ*_2_ rotation, are 0.2328, 0.0005, and 9 × 10^−7^, respectively. These findings demonstrate that Se···N, S···O, and Se···O INIs play a crucial role in reducing *S*_conf._, achieving conformational control comparable to pseudo-ladder [53,54] and hydrogen-bonded systems [55,56]. Importantly, INIs introduce superior design flexibility and synergistic effects, allowing simultaneous regulation of electronic structures [57].

The structural characteristics of multiple rotatable *σ*-bonds in NFREA systems necessitate a systematic assessment of the possibility of coexisting conformations. In NFREAs possessing *σ*_1_ and *σ*_2_ bonds, a *K*-value-mediated four-quadrant plot analysis identifies (0.01, 0.01) as the critical threshold dividing isomer-free and isomer-prone regions (Fig. 1i). This reveals that only candidates with dual *K*-values below 0.01, exemplified by 3TT-SeS and 3TT-SeSe, exhibit negligible isomer coexistence and low *S*_conf_. Cost-effectiveness emerges as an equally crucial consideration in the practical development of NFREAs. A material-only cost (MOC) analysis demonstrates that the key building block 3R-SeS exhibits significantly lower synthesis costs compared to 3R-SeSe (Fig. S6 and Table S5) [58], thereby establishing 3TT-SeS as the optimal candidate, combining low cost with low *S*_conf._ among all model NFREAs. These results demonstrate both the significant potential of selenium-containing NFREAs in advancing OSC technology and the urgent requirement for environmentally friendly and efficient synthetic strategies for these materials.

### Characterization of low-*S*_conf._ NFREAs

**Figure 2.**
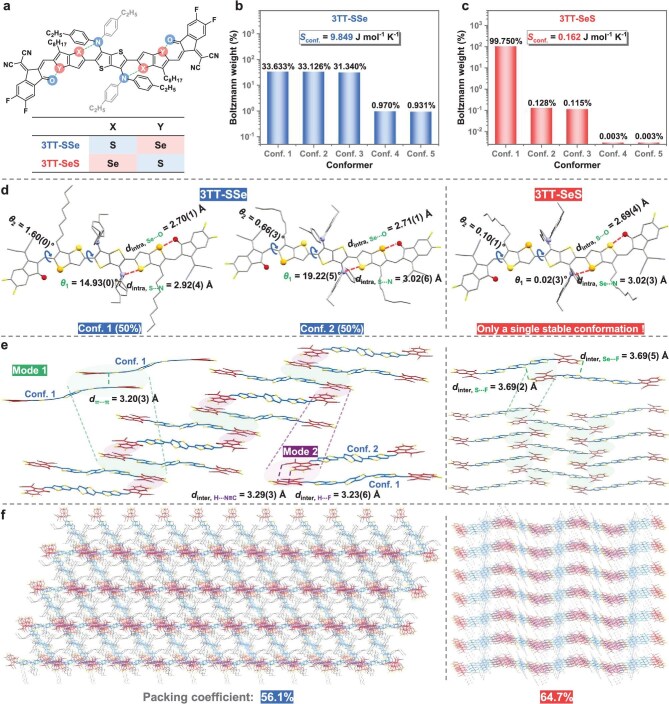
Characterizations of low-*S*_conf._ NFREAs. (a) Chemical structures. (b and c) Conformer population of 3TT-SSe (b) and 3TT-SeS (c) as calculated through quantum chemistry. (d) Single-crystal structures. (e) Intermolecular stacking modes. (f) Crystal packing diagrams.

Following the DFT calculation results, we synthesized the optimal candidate, 3TT-SeS, and the secondary candidate, 3TT-SSe, as a comparative reference. The chemical structures and synthesis details for both NFREAs are depicted in Figs 2a and S7. Both acceptors demonstrated good thermal stability, with thermal decomposition temperatures (corresponding to 5% weight loss) exceeding 300°C (Fig. S8). To gain a deep understanding of INIs within the acceptors, natural bond orbital (NBO) analysis was conducted. The results revealed significant orbital overlaps: (i) between the n-orbital of nitrogen lone pair and σ*(S−C or Se−C) anti-bonding orbitals, and (ii) between the 2p electron lone pair on the carbonyl oxygen atom and σ*(S−C or Se−C) orbitals (Fig. S9). The orbital interaction energies (*E*^(2)^) that exhibit a negative correlation with INI strength (*S*_INI_ values) were calculated as −0.83/−3.24 kcal mol^−1^ for *n*(N)→σ*(S−C)/σ*(Se−C) and −6.43/−1.37 kcal mol^−1^ for *n*(O)→σ*(Se−C)/σ*(S−C), respectively. These results not only confirm the existence of INIs within both NFREAs, but also reveal a rational combination: the transformation from weak S···N and strong Se···O INIs in 3TT-SSe into balanced Se···N and S···O INIs in 3TT-SeS, which is expected to restrict the *σ*_1_-bond rotation, thereby leading to the effective elimination of rotational freedom in 3TT-SeS.

To investigate the impact of rational INI combinations on *S*_conf._ of NFREAs, we employed a conformational search program to generate 2000 conformations through *σ*-bond rotation simulations in the conjugated backbone [59]. The top five low-energy conformers (Confs. 1–5) were selected, and their population ratios were calculated using the Boltzmann distribution in relative Gibbs free energies. As shown in Fig. 2b–c, Fig. S10, and Table S6, 3TT-SSe exhibited three dominant conformers with comparable populations (33.633%, 33.126%, and 31.340%), while 3TT-SeS demonstrated an almost exclusive preference for a single conformation (99.750%), thus resulting in a significantly reduced *S*_conf._ of 0.162 J mol^−1^ K^−1^, much smaller than that of 3TT-SSe (9.849 J mol^−1^ K^−1^). This stark contrast in conformational diversity highlights the accuracy of computer-aided screening in achieving conformer-free and low-*S*_conf._ NFREAs.

To explore the influence of reduced *S*_conf._ on molecular conformation and aggregation structure, needle-shaped crystals of 3TT-SSe and 3TT-SeS were grown using the solvent diffusion method for single-crystal X-ray diffraction analysis. Crystallographic data revealed that 3TT-SSe crystallized in the triclinic *P*-1 space group, while 3TT-SeS belonged to the monoclinic *P*2_1_*/c* space group (Tables S7 and S8). As illustrated in Figs 2d, S11, and Table S9, the 3TT-SSe unit cell existed as a mixture of two distinct conformers (1:1 ratio), characterized by the torsion angles θ_1_ = 14.93° and 19.22° for Confs. 1 and 2, respectively. The intramolecular distances (*d*_intra_) for S···N (2.92 Å) and Se···O (2.70 Å) are notably shorter than the sums of relevant van der Waals radii (*r*_w, S···N_ = 3.35 Å and *r*_w, Se···O_ = 3.40 Å), supporting the existence of S···N and Se···O INIs. Notably, the coexistence of dual conformers in ‌3TT-SSe mainly arises from the insufficient restriction of *σ*_1_-bond rotation, attributed to the relatively weak S···N INIs with small *S*_INI_ values (0.254 for Conf. 1 and 0.120 for Conf. 2). In contrast, 3TT-SeS displayed a monoconformational behavior around the *σ*_1_-bond with near-planar conjugated backbone (θ_1_/θ_2_ = 0.02°/0.10°). This conformation is stabilized by moderate-strength Se···N (*d*_intra, Se···N_ = 3.02 Å) and S···O (*d*_intra, S···O_ = 2.69 Å) INIs, as confirmed by the higher *S*_INI_ values (0.358 for Se···N; 0.563 for S···O). These results demonstrate that the INI combination strategy effectively restricts the rotation of multiple *σ*-bonds, achieving an exclusive single conformation in 3TT-SeS.

Comparative analysis of packing architectures revealed distinct molecular assembly behaviors (Fig. 2e and f). The 3TT-SSe crystal possessed a dual-mode assembly, featuring intermolecular π···π interactions (*d*_π···π_ = 3.20 Å) in Mode 1 and dual intermolecular hydrogen bonds (*d*_inter, C≡N···H_ = 3.29 Å and *d*_inter, F···H_ = 3.23 Å) in Mode 2. Notably, mediated by intermolecular S···F and Se···F interactions (*d*_inter, S···F_ = 3.69 Å and *d*_inter, Se···F_ = 3.69 Å), 3TT-SeS crystallized in a slipped-stack arrangement, where each molecule was surrounded by four neighbors. The slightly increased intermolecular distances observed in 3TT-SeS may be attributed to the larger atomic radius of selenium atoms [60]. Therefore, the denser packing mode resulted in a significant increase in the packing coefficient (*C*_packing_) from 56.1% for 3TT-SSe to 64.7% for 3TT-SeS, which facilitates efficient charge transport (Fig. 2f) [61]. The above findings collectively demonstrate that the rational combination of INIs may not only reduce the *S*_conf._ but also promote the formation of highly ordered self-assembled structures.

### Photophysical properties and stacking behavior

**Figure 3.**
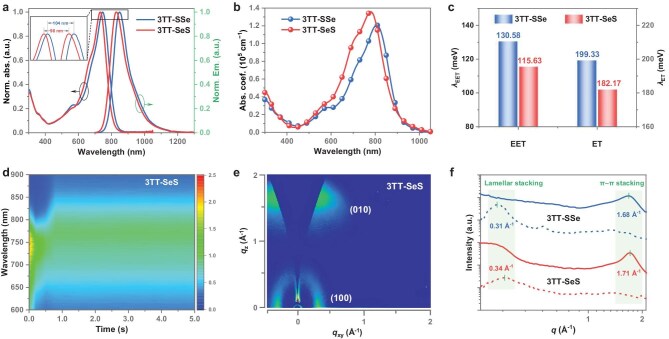
Photophysical properties and stacking behavior. (a) Normalized UV–vis absorption and photoluminescence spectra of 3TT-SSe and 3TT-SeS in dilute chloroform solutions. (b) Absorption spectra in thin films. (c) Calculated reorganization energies for exciton energy transfer (EET) and electron transport (ET) processes. (d) Time-dependent contour maps of *in-situ* UV–vis absorption spectra for the 3TT-SeS film. (e) 2D-GIWAXS pattern of the neat 3TT-SeS film. (f) In-plane (dashed lines) and out-of-plane (solid lines) line-cut profiles of the 2D-GIWAXS data.

The photophysical properties of 3TT-SSe and 3TT-SeS were systematically probed through a combination of spectroscopic techniques and theoretical calculations. In dilute chloroform solutions, 3TT-SSe exhibited maximum absorption and emission peaks at 745 and 849 nm, respectively. In contrast, 3TT-SeS displayed blue-shifted absorption (732 nm) and emission (830 nm) peaks (Fig. 3a and Table S10). Upon transitioning to the solid state, both acceptors exhibited red-shifted absorption/emission maxima: 3TT-SSe at 807/908 nm and 3TT-SeS at 781/872 nm (Fig. 3b and Fig. S12). In addition, the 3TT-SeS thin film demonstrated a higher molar extinction coefficient (1.36 × 10^5^ cm^−1^) compared to 3TT-SSe (1.21 × 10^5^ cm^−1^), suggesting a tighter packing for 3TT-SeS [62], consistent with single-crystal X-ray diffraction results. Combining absorption and emission properties, we found that the Stokes-shifts (Δ*v*) for 3TT-SeS in solution and film (1613 and 1336 cm^−1^, respectively) are slightly smaller than those of 3TT-SSe (1644 and 1378 cm^−1^), indicating an INI combination-induced molecular rigidity enhancement, which is beneficial for suppressing non-radiative energy loss (Δ*E*_nr_) in OSCs [63,64]. To corroborate the enhanced molecular rigidity, reorganization energies for exciton energy transfer (*λ*_EET_) and electron transport (*λ*_ET_) were calculated (Fig. 3c and Fig. S13, Table S11). The *λ*_EET_ values for 3TT-SSe and 3TT-SeS were found to be 130.58 and 115.63 meV, respectively, aligning with the observed trend in Stokes-shift values. Furthermore, 3TT-SeS exhibited a lower *λ*_ET_ (182.17 meV) than 3TT-SSe (199.33 meV), facilitating efficient charge transport according to the Marcus theory [65].

The electrochemical properties of the two NFREAs were investigated by cyclic voltammetry experiments (Fig. S14). The energy levels of the lowest unoccupied molecular orbital (*E*_LUMO_) and highest occupied molecular orbital (*E*_HOMO_) were estimated to be −4.08 and −5.58 eV for 3TT-SSe, and −4.04 and −5.60 eV for 3TT-SeS, respectively. Furthermore, the HOMO levels determined by ultraviolet photoelectron spectroscopy are −5.62 and −5.64 eV (Fig. S15), respectively. Based on the optical bandgaps (1.34 and 1.37 eV) derived from the absorption onset, the corresponding LUMO levels are calculated to be −4.28 and −4.27 eV. The high-lying *E*_LUMO_ of 3TT-SeS is consistent with DFT calculations (Fig. S16), thus potentially achieving a higher open-circuit voltage (*V*_oc_) in corresponding OSCs. Furthermore, *in-situ* UV–vis absorption spectra (Fig. 3d and Fig. S17) during film formation demonstrated accelerated crystallization dynamics for 3TT-SeS, contributing to the establishment of an ordered molecular arrangement. Two-dimensional grazing incidence wide-angle X-ray scattering (2D-GIWAXS) revealed a shorter lamellar stacking distance and decreased π–π stacking distance for 3TT-SeS (18.48 and 3.67 Å) than 3TT-SSe (20.27 and 3.74 Å), consistent with its denser packing and higher molar extinction coefficient of 3TT-SeS (Fig. 3e–f and Fig. S18, Table S12). Subsequently, the crystal coherence length (CCL) and the paracrystalline disorder factor (*g*-factor) were calculated to be 16.61 Å/0.189 for 3TT-SSe and 25.13 Å/0.152 for 3TT-SeS, respectively. The increased CCL and reduced *g*-factor further highlight the role of the INI combination strategy in promoting a tighter and more ordered structure in 3TT-SeS thin film.

### Photovoltaic performance and device physics

**Figure 4.**
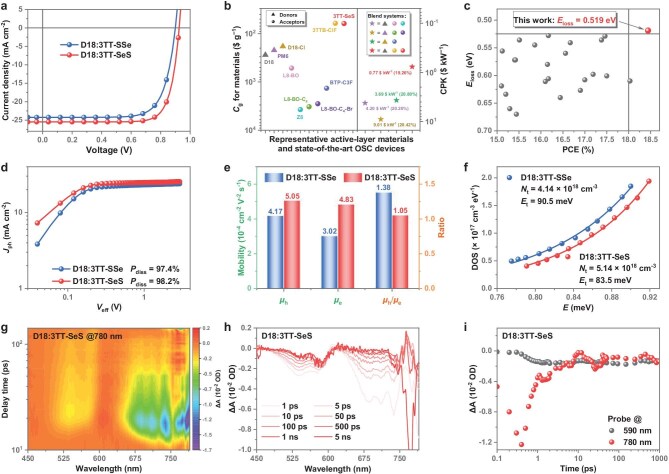
Photovoltaic performance and device physics. (a) *J*–*V* characteristics for optimized OSCs. (b) Statistics of *C*_g_ and CPK values for active-layer materials. (c) *E*_loss_ value against PCE plots based on the reported efficient NFREA-based OSCs (the list of data is available in Supplementary Table 24). (d) *J*_ph_–*V*_eff_ plots of the D18:3TT-SSe- and D18:3TT-SeS-based devices. (e) Statistical diagram of charge transport properties. (f) Electron DOS of the two devices. (g–i) 2D color plots of TA spectra (g), representative TA spectra recorded at indicated delay times (h), and TA kinetic probed at 590 and 780 nm (i) for the 3TT-SeS-based blend film.

To explore the photovoltaic performance of two NFREAs, we fabricated OSC devices featuring an architecture of indium tin oxide/2PACz/active layer/PDINN-S/Ag, utilizing polymer D18 (Figs S19–S20) as the donor material. Following established solvent processing principles [11], we employed a chloroform/*o*-xylene (*o*-XY) co-solvent system with a volume ratio of 0.88 : 0.12 to optimize NFREA-based OSCs (Fig. S21). The optimized device parameters and detailed optimization procedures are summarized in Tables 1 and Tables S13–S14. As demonstrated in Fig. 4a and Fig. S22, the 3TT-SSe-based device exhibited a moderate PCE of 16.17%, accompanied by a *V*_oc_ of 0.900 V, a short-circuit current density (*J*_sc_) of 24.22 mA cm^−2^, and a fill factor (FF) of 74.20%. Remarkably, the 3TT-SeS-based device achieved superior photovoltaic performance with simultaneous increases in *V*_oc_ to 0.925 V, *J*_sc_ to 25.47 mA cm^−2^, and FF to 78.21%, thus yielding a high PCE of 18.44%. The improved *J*_sc_ value was further supported by the consistency between the external quantum efficiency (EQE) spectra and the absorption spectra of the blend films (Figs S23–S24), showing integrated *J*_sc_ values of 25.10 and 23.77 mA cm^−2^ for the D18:3TT-SSe and D18:3TT-SeS devices, respectively. To further unlock the potential of 3TT-SeS with reduced *S*_conf._, we incorporated a reported NFREA 3TTB-ClF [26] as a guest acceptor into the binary D18:3TT-SeS host blend. This strategy resulted in a significant boost in the PCE of the 3TT-SeS-based device to 19.26% (independently certified as 18.75% by the National Photovoltaic Industry Metrology and Testing Center (NPVM), China; Figs S25–S28), representing a record-high efficiency for low-cost NFREA-based OSCs. To evaluate operational stability, encapsulated devices were subjected to continuous one-sun illumination in a nitrogen atmosphere. The NFREA-based devices retained ∼80% of their initial PCEs after 700 h, demonstrating their promising photostability for practical applications (Fig. S29).

**Table 1.** Detailed photovoltaic device parameters of the optimized OSCs.

Active layers	*V*_oc_ (V)	*J*_sc_ (mA cm^−2^)	calc. *J*_sc_^(^^a)^ (mA cm^−2^)	FF (%)	PCE^(^^b)^ (%)
D18:3TT-SSe	0.900 (0.900 ± 0.001)	24.22 (24.01 ± 0.22)	23.77	74.20 (74.90 ± 0.31)	16.17 (16.05 ± 0.11)
D18:3TT-SeS	0.925 (0.824 ± 0.001)	25.47 (25.20 ± 0.26)	25.10	78.21 (77.98 ± 0.24)	18.44 (18.31 ± 0.10)
D18:3TT-SeS: 3TTB-ClF	0.939 (0.939 ± 0.001)	25.63 (25.41 ± 0.23)	25.14	80.03 (79.72 ± 0.30)	19.26 (19.10 ± 0.14)
D18:3TT-SeS: 3TTB-ClF^(^^c)^	0.938	25.11	—	79.60	18.75

(a) The *J*_sc_ values calculated from the EQE curves

(b) The error bars correspond to the standard deviation of 30 independent devices; ^(c)^ Certified by NPVM, China.

To evaluate the commercial viability of low-*S*_conf._ NFREA-based OSCs, we conducted a comprehensive cost analysis (*C*_g_, cost-per-gram) comparing state-of-the-art FREAs with 3TT-SeS. Using an established cost-calculation model [17] (Fig. 4b), we determined the *C*_g_ values for each material (Figs S30–S39 and Tables S15–S25 for detailed synthesis schemes and calculations). Remarkably, 3TT-SeS demonstrates a significant cost advantage with a *C*_g_ of 61.69$ g^−1^, achieving >90% reduction relative to reported high-performance FREAs (518.10, 3263.32, and 1352.50$ g^−1^ for L8-BO, L8-BO-C_4_, and BTP-C3F, respectively). To better assess commercial potential, we further calculated the power generation cost (cost-per-kilowatt, CPK, $ kW^−1^) for active-layer materials. Compared with reference systems (D18: L8-BO: Z8, 4.20$ kW^−1^ at 20.20% PCE; PM6: L8-BO-C_4_: L8-BO-C_4_-Br, 9.01$ kW^−1^ at 20.42% PCE; and D18-Cl: BTP-C3F, 3.69$ kW^−1^ at 20.80% PCE), 3TT-SeS-based blend system shows exceptional cost-performance, with a notably low CPK of 0.77$ kW^−1^. This dramatic cost reduction, achieved through our *S*_conf._-oriented molecular design paradigm, highlights the great potential in the development of cost-effective NFREA-based OSCs.

To elucidate the origin of *V*_oc_ enhancement, we conducted a comprehensive energy loss (*E*_loss_) analysis (Table S27 and Fig. S40). Bandgap (*E*_g_) determinations via UV–vis/PL spectral crossover revealed *E*_g_ values of 1.428 eV (3TT-SSe) and 1.444 eV (3TT-SeS) (Fig. S41). Therefore, the calculated total *E*_loss_ values of 0.528 eV (D18:3TT-SSe) and 0.519 eV (D18:3TT-SeS), derived from *E*_loss_ = *E*_g_ — *qV*_oc_, demonstrate that the 3TT-SeS-based binary system achieves the smallest reported *E*_loss_ (0.519 eV) among high-performance NFREA-based OSCs with PCEs exceeding 15% (Fig. 4c and Table S26). Furthermore, the non-radiative energy loss (Δ*E*_nr_), determined by the EQE of electroluminescence devices (EQE_EL_), is considered the primary contributor to *E*_loss_ in OSCs. The EQE_EL_ values were found to be 2.16 × 10^−4^ and 2.87 × 10^−4^ for the devices based on D18:3TT-SSe and D18:3TT-SeS, respectively, demonstrating a strong correlation with the measured solid-state PLQYs of 5.31% (3TT-SSe) and 5.97% (3TT-SeS) [66–68]. Using the equation Δ*E*_nr_ = –*kT* ln(EQE_EL_), the corresponding Δ*E*_nr_ values were calculated to be 0.211 and 0.204 eV for these systems, respectively. The suppressed Δ*E*_nr_ observed in the 3TT-SeS-based device aligns with the well-documented enhancement in molecular rigidity.

To gain a profound understanding of the mechanisms underlying the different *J*_sc_ and FF observed in the two binary devices, we conducted a systematic investigation of their charge generation and transport processes. The exciton dissociation probability (*P*_diss_) was assessed by analyzing the correlation between the photocurrent density (*J*_ph_) and the effective voltage (*V*_eff_). As shown in Fig. 4d, the D18:3TT-SeS exhibits a higher *P*_diss_ value of 98.2% compared to the 3TT-SSe-based device (97.4%), enabling a greater probability of and thus leading to the improved *J*_sc_. Besides, space-charge-limited current measurements (Figs 4e and S42) demonstrated improved and balanced charge transport in the D18:3TT-SeS-based device, with hole/electron mobilities (*μ*_h_/*μ*_e_) of 5.05/4.83 × 10^−4^ cm^2^ V^−1^ s^−1^ (*μ*_h_/*μ*_e_ ratio = 1.05) vs. 4.17/3.02 × 10^−4^ cm^2^ V^−1^ s^−1^ (ratio = 1.38) for D18:3TT-SSe. The optimized charge transport in the 3TT-SeS-based device facilitates reduce charge accumulation and recombination, thereby contributing to the observed improvements in both *J*_sc_ and FF.

To investigate the charge recombination kinetics, we systematically analyzed the dependences of *V*_oc_ and *J*_sc_ on light-intensity (*P*_light_) (Fig. S43). The relationships follow *V*_oc_ ∝ (*nk*_B_*T*/*q*)ln(*P*_light_) and *J*_sc_ ∝ (*P*_light_)*^α^*, where *k*_B_ is the Boltzmann constant, *T* is the absolute temperature, and *q* is the elementary charge. For the 3TT-SSe-based device, the *n*/*α* values were found to be 1.29/0.96 and 1.11/0.99 for 3TT-SSe and 3TT-SeS-based device, respectively. These values approaching unity indicate suppressed trap-assisted recombination and bimolecular recombination in the D18:3TT-SeS device. Transient photovoltage (TPV) and transient photocurrent measurements revealed an extended charge carrier lifetime (τ = 7.98 μs), enhanced charge carrier density, reduced recombination exponent (*R* = 2.36), and lowered nongeminate recombination rate coefficient (*k* = 1.20 × 10^−16^ m^3^ s^−1^) in the 3TT-SeS-based device (Fig. S44). Further insight into the electron states was gained through capacitance-derived density of states (DOS) analysis. The electron DOS distribution follows $g( E )\ = \frac{{{N}_{\mathrm{t}}}}{{{E}_{\mathrm{t}}}}\ {\mathrm{exp}}[ { - \frac{{E - {E}_{{\mathrm{LUMO}}}}}{{{E}_{\mathrm{t}}}}} ]$, where *g*(*E*) is obtained by extracting $C_{\mathrm{\mu }}^{\mathrm{n}}$ from $C_{\mathrm{\mu }}^{\mathrm{n}} = L{q}^2g( E )$; *N*_t_ represents the total density per unit volume; and *E*_t_ is the energy for the exponential tail distribution that characterizes energetic disorder. As shown in Fig. 4f, the 3TT-SeS blend exhibits a high *N*_t_ of 5.14 × 10^18^ cm^−3^ and a lower *E*_t_ of 83.5 meV compared to the 3TT-SSe counterpart (4.14 × 10^18^ cm^−3^ for *N*_t_ and 90.5 meV for *E*_t_). The enhanced carrier density and reduced energetic disorder correlates with the suppressed energy loss observed in the 3TT-SeS-based device.

Hole-transfer (HT) dynamics were probed through transient absorption spectroscopy, in which an excitation wavelength of 780 nm was employed to selectively excite acceptors (Figs 4g and S45). Upon photoexcitation, immediate ground state bleaching signals (>650 nm) of the acceptors decayed, accompanied by donor-specific bleaching emergence (520–600 nm), indicating the existence of an HT channel that facilitates exciton dissociation in blend films (Fig. 4h). Biexponential fitting of kinetics (Fig. 4i) revealed two distinct processes: the ultrafast exciton dissociation at the donor/acceptor interface (τ_1_) and slower exciton diffusion in the donor phase toward the interface before dissociation (τ_2_). Notably, the 3TT-SeS blend exhibited accelerated dynamics with τ_1_ = 0.26 ps and τ_2_ = 5.61 ps, surpassing the 3TT-SSe counterpart (τ_1_ = 0.74 ps, τ_2_ = 8.30 ps). These findings indicate a superior hole transfer process in the D18:3TT-SeS blend, aligning with the excellent performance.

### Micromorphology characterization

**Figure 5.**
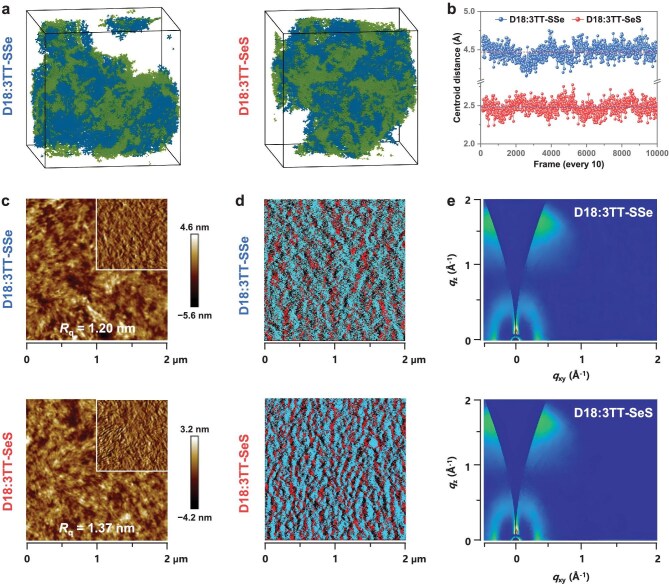
Micromorphology characterization of blend films. (a) Snapshots of MD simulation (blue and green balls represent donor and acceptor molecules, respectively). (b) Statistical diagram of donor–acceptor centroid distance of the two blend systems. (c) AFM height images (2 μm × 2 μm) (insets are phase images of 1 μm × 1 μm size). (d) The combined AFM-IR images of the two blend films. (e) 2D-GIWAXS patterns for D18:3TT-SSe and D18:3TT-SeS, respectively.

The molecular dynamics (MD) simulations were employed to investigate the microstructure of blend films. Figure 5a presents equilibrium-state snapshots of the simulated molecular blends. To quantitatively analyze phase separation behavior, we calculated the donor–acceptor centroid distance distribution and obtained the corresponding average values (Fig. 5b). The results reveal a significantly shorter average centroid distance in the D18:3TT-SeS blend (2.48 Å) compared to the D18:3TT-SSe system (4.47 Å), suggesting the formation of a more compact and ordered microstructure with enhanced separation between D18 and 3TT-SeS components. Surface morphology and phase-separation patterns were probed through atomic force microscopy (AFM) and AFM-coupled infrared spectroscopy (AFM-IR). As shown in Fig. 5c, the root-mean-square roughness (*R*_q_) of the 3TT-SSe- and 3TT-SeS- based blend films were determined to be 1.20 and 1.37 nm, respectively, suggesting a more pronounced surface crystallization in the D18:3TT-SeS film. Complementary characterization through Fourier-transform infrared spectroscopy (Fig. S46) identified distinct vibrational features for the donor (1579 cm^−1^) and acceptor (1148 cm^−1^) components. Advanced AFM-IR phase analysis (Fig. S47) revealed both D18 and 3TT-SeS formed a denser fibrillar network structure in the 3TT-SeS blend compared to the 3TT-SSe blend. The further overlay analysis (Fig. 5d) demonstrated entangled donor/acceptor fibrillar domains and higher-quality bicontinuous interpenetrating network morphology observed in the D18:3TT-SeS blend film. The molecular stacking characteristics of both blend films were investigated using 2D-GIWAXS (Figs 5e and S48, Table S28). The blend films exhibited the preferential face-on orientation observed in their neat counterparts, with π–π stacking distances of 3.79 Å for the 3TT-SSe blend and 3.74 Å for the 3TT-SeS film. Notably, the 3TT-SeS-based blend demonstrated superior structural ordering, as evidenced by its increased CCL (21.67 Å) and reduced *g*-factor (0.166) compared to the 3TT-SSe counterpart (CCL = 13.96 Å, *g*-factor = 0.208). These results demonstrate that 3TT-SeS, with its lower *S*_conf._, promotes tighter molecular packing and favorable film micromorphology, thus providing a more efficient charge transport pathway and rationalizing the improved *J*_sc_ and FF in corresponding OSC device.

## CONCLUSION

In this work, we proposed a low-*S*_conf._ design of NFREAs for highly cost-effective OSCs. Through comprehensive DFT calculations, we identified 3TT-SeS as an optimal low-*S*_conf._ and low-cost NFREA via an INI combination strategy. Both theoretical and experimental investigations confirmed that the 3TT-SeS indeed achieves an ultralow *S*_conf._ (0.162 J mol^−1^ K^−1^) and a unique conformer-free state, thus yielding densely ordered crystal packing, along with improved charge transport property and suppressed energetic disorder. These advancements enabled 3TT-SeS-based OSCs to achieve a remarkable PCE of 19.26% (independently certified at 18.75%) in ternary devices, setting a new efficiency benchmark for low-cost OSCs. Importantly, the MOC of 3TT-SeS-based OSC was much lower than that of the state-of-the-art FREA-based systems. This breakthrough not only addresses the long-standing trade-off between cost and efficiency in organic photovoltaics but also presents a scalable, industrially viable strategy for developing next-generation organic semiconductors.

## MATERIALS AND METHODS

Detailed materials and methods are available in the online supplementary data.

## Supplementary Material

nwag177_Supplemental_Files
