# Supplementary material for: Conformational entropy tuning in nonfused-ring electron acceptors for low-cost and record-efficiency organic solar cells
Source: Natl Sci Rev. 2026 Mar 19;13(9):nwag177. doi: 10.1093/nsr/nwag177 (PMC13221954; doi:10.1093/nsr/nwag177)
Supplement: nwag177_Supplemental_Files [file nwag177_supplemental_files.zip › Supplementary data.pdf]

Supporting Information

Conformational entropy tuning in nonfused-ring electron acceptors for low-cost and record-efficiency organic solar cells

Xiaobin Gu[1,†], Rui Zeng[2,†], Fei Han[2], Jing Li[3], Senke Tan[2], Jikai Lv[1], Na Yu[4], Lixuan Kan[2], Hao Li[2], Yuqi Hou[1], Yunhao Cai[1], Zhixiang Wei[5], Zheng Tang[4], Lianrui Hu[3], Feng Liu[2,*], Xin Zhang[1,*] and Hui Huang[1,6,7,*]

Table of contents

1 Supplementary Methods 2
- 1.1 Materials and general characterization 2
- 1.2 Computational method 3
- 1.3 Single-crystal growth and X-ray diffraction analysis 4
- 1.4 Solar cell device fabrications and measurements 4
- 1.5 Energy loss analysis 5
- 1.6 Analysis of charge generation and extraction 6
- 1.7 Measurements of the hole and electron mobility by the SCLC method 6
- 1.8 TPV and TPC measurement 6
- 1.9 TA spectroscopy measurements 6
- 1.10 Morphology characterization 6
- 1.11 AFM-IR measurements 7

2 Synthetic Section 8
- 2.1 General procedure for the synthesis of 2a and 2b 8
- 2.2 General procedure for the synthesis of 3a and 3b 9
- 2.3 General procedure for the synthesis of 4a and 4b 10
- 2.4 General procedure for the synthesis of 3TT-SSe and 3TT-SeS 11

3 Supplementary Figures 12

4 Supplementary Tables 80

5 Supplementary References 116

1 Supplementary Methods

1.1 Materials and general characterization

The polymer donor D18 (M_n = 5.95 kDa, PDI = 2.08, Fig. S21) and hole-transport-layer material 2PACz were purchased from Solarmer Materials Inc. The other reagents were purchased from Energy Chemical, Macklin, Shanghai Haohong Scientific Co.,Ltd., Adamas-beta®, and used as received unless otherwise specified.

Details of the synthetic procedures for the two NFREAs and the corresponding structural characterization are presented in Figs S7 and S49–S65. ^{1}H and ^{13}C NMR spectra were recorded on a JEOL JNM-ECZ500R (500 MHz) spectrometer with $CDCl_3$ as a solvent and tetramethylsilane as an internal reference.

UV−vis absorption and photoluminescence spectra were carried out with Agilent Cary 60 spectrometer and Edinburgh steady state/transient fluorescence spectrometer (FLS1000), respectively.

Electrochemical measurements were carried out under nitrogen in a deoxygenated solution of tetra-*n*-butylammonium hexafluorophosphate (0.1 M) in CH_3CN using a computer-controlled CHI600 electrochemical workstation, a glassy-carbon working electrode coated with samples, a platinum-wire auxiliary electrode, and Ag/AgCl as a reference electrode. Potentials were referenced to the ferrocenium/ferrocene ($FeCp_2^{0/+}$) couple by using ferrocene as an internal standard. According to the equations of $E_{HOMO} = -e(\varphi_{ox} + 4.8)$ (eV) and $E_{LUMO} = -e(\varphi_{red} + 4.8)$ (eV), where φ_{ox} and $_{red}$ are the onset of oxidation and reduction potential versus $FeCp_2^+/FeCp_2^0$, which has formal potential of − 4.8 eV in the Fermi scale.

The FT-IR spectra were recorded on a Nicolet iS50 FT-IR with a built-in diamond attenuated total reflection.

1.2 Computational method

Density functional theory (DFT) calculations were performed using the Gaussian 16 program with the B3LYP/6-31G(d) level.[1] All-electron triple-ξ valence basis sets with polarization functions (6-31G) are used for all atoms. Geometry optimizations were performed with full relaxation of all atoms. Calculations were performed in gas phase without solvent effects. Vibrational frequency calculations were performed to check that the stable structures had no imaginary frequency. Track visualization were carried out by Multiwfn and VMD.[2]

In order to obtain the initial molecular conformation of the system, we first conducted a conformation search using Molclus[3]/xTB[4, 5]/Gaussian 16[6]/ORCA[7] software to determine the most stable conformer. In the conformation search process, the xTB software was used to randomly generate 2000 different molecular conformations by molecular dynamic. Then xTB software was used to batch optimize the geometry of 2000 clusters at GFN0-xTB[8] level. Next, the first 150 configurations was used to batch optimize the geometry of 2000 clusters at GFN2-xTB[8] level. Next, the first 5 configurations were selected by energy ranking for further structural optimization and frequency analysis with higher precision at the B3LYP[9]-D3(BJ)[10, 11]/def2-SVP level[12, 13]. Finally, the single point energy was calculated at the M062X-D3[14]/def2-QZVPP[12, 13] level. The most table conformation was obtained by Gibbs free energy ordering by Shermo software[15]. The VMD[16] software was used to visualize the structure of molecular clusters.

1.3 Single-crystal growth and X-ray diffraction analysis

Liquid phase diffusion method was employed to obtain the needle-like diffraction quality crystals. Each acceptor material (1.5 mg) and $CHCl_3$ (0.5 mL) was added to NMR tube and fully dissolved, followed by the slow addition of 0.5 mL methanol. The system was tightly sealed and left standing for a few days (5-7 days) until needle crystals emerged. X-ray crystallographic data were collected at 193 K on Rigaku XtaLAB PRO MM007 DW. The crystal was kept at during data collection. The structure was solved by intrinsic phasing method (SHELXT) and refined by least squares method (SHELXL) integrated in Olex2. The detailed crystal parameters are summarized in Table S7.

The packing coefficient (C_{packing}) quantifies atomic packing efficiency within a unit cell as the fraction of volume occupied by atoms (typically modeled as rigid spheres). Mathematically, it is expressed as: $C_{\text{packing}} = n \cdot v/V$, where n is the number of atoms per unit cell, v is the atom volume (= $4\pi r^3/3$, r as the atomic radius), and V is the unit cell volume. This parameter reflects the density of atoms arrangement, with higher values indicating more efficient packing.

1.4 Solar cell device fabrications and measurements

The organic solar cells were fabricated with a conventional architecture of ITO/2PACz/active layer/ PDINN-S/Ag. Patterned ITO glass was successively cleaned twice in an ultrasonic bath by detergent, deionized water, acetone, and isopropyl alcohol for 15 min each and then dried under dry oven. The precleaned substrates were treated in an ultraviolet-ozone chamber for 20 min, then the self-assembled monomolecular layer of 2PACz was deposited onto the ITO surface by spin-coating and baked at 100 °C for 5 min. Active layer was fabricated by spin-coating a mixed chloroform solution of D18 and acceptors (weight ratio of 1:1.2) in precursor CF/*o*-XY co-solvent system (with 3 mg mL^{-1} solid additive of DIB) at the donor concentration of 6.5 mg mL^{-1} on the ITO/2PACz substrate in a nitrogen glove box, followed by annealing treatment at 100 °C 10 min. Then, a thin PDINN-S layer was spin-coated on the active layer, followed by the deposition of Ag.

The cells were characterized under a temperature of 25–30 °C in a glove box filled with nitrogen. The current density-voltage (*J-V*) curves were measured with a computer-controlled Keithley 2450 Source Measure Unit under AM 1.5G white light source (Enlitech), the optical power at the sample was 100 mW/cm^2. Simulator irradiance was characterized using a calibrated spectrometer and illumination intensity was set using a certified silicon diode (SRC-2020, Enlitech). External quantum efficiency (EQE) values of the devices were measured using a QE-R3011 instrument (Enli Technology Co. Ltd., Taiwan, China) with a scan increment of 5 nm per point.

1.5 Energy loss analysis

The E_{loss} of OSCs is defined as the difference between the bandgap (E_g) of the photoactive materials and the V_{oc} of the device, which can be classified into three parts: $E_{\mathrm{loss}} = (E_g - E_{\mathrm{CT}}) + \Delta E_r + \Delta E_{\mathrm{nr}} = \Delta E_{\mathrm{CT}} + \Delta E_r + \Delta E_{\mathrm{nr}}$. The E_g is determined by the crossing point between the normalized absorption and emission spectra of the blend films.

Sensitive EQE (sEQE) measurement was performed as follows: A 150 W quartz halogen lamp (LSH-75, Newport) acted as a light source, passing through the monochromator (CS260-RG-3-MC-A, Newport) to provide an adjustable monochromatic light source for testing, and then emitted an optical signal at a 173 Hz frequency through the chopper (3502 Optical Chopper, Newport) and focused on the OSC devices. The current generated by the device was amplified by the front-end current amplifier (SR570, Stanford) to reduce the impact of the noise signal. The final signal was collected and analyzed by a Phase-locked Amplifier (SR830 DSP Lock-In Amplifier, Stanford).

Fitting of E_{CT}: The tails of the EQE spectra are determined by a sEQE setup, which is composed with a halogen lamp, a monochromator, a current amplifier and a lock-in amplifier. A set of long pass filters are used to cut the higher order wavelengths from the monochromator, which generated monochromatic light for test. To further extend the range of the EQE spectra measured by the sensitive EQE setup, electroluminescence (EL) spectra of the solar cells are also measured, using a small injection current. Since EL spectra of the OSCs are dominated by CT state emission, they can be converted to CT state absorption spectra using the reciprocal relation. The tail of the EQE spectrum, corresponding to CT absorption, was used to determine the energy of CT state (E_{CT}) by fitting the equation derived in the framework of Marcus theory.

$$EL(E) = EQE(E)\phi_{BB}(E)[\exp\left(\frac{qV}{kT}\right) - 1] \tag{1}$$

$$EQE(E) = \frac{fE}{\sqrt{4\pi\lambda kT}}\exp\left(-\frac{(E_{CT}+\lambda+E)^2}{4\lambda kT}\right) \tag{2}$$

To avoid an arbitrary fitting, two boundary conditions are imposed. Firstly, we calculate the lower limit for the radiative recombination voltage loss ($V_{r,sq}$) for a solar cell as a function of E_{CT} using the Shockley-Quessior theory, assuming that E_{CT} is the effective energy of the bandgap of an OSCs. Then, the Gaussian region in the lower energy part of the EQE spectrum is selected and fitted. During the fitting process, a set of f values typically in the range between 0.0001 to 0.1 are used as constant input values, while E_{CT} and λ are left as fit parameters.

1.6 Analysis of charge generation and extraction

J_{ph} is defined as $J_{ph} = J_L - J_D$, where J_L and J_D represent the current densities that are illuminated and in the dark, respectively. V_{eff} is defined as $V_0 - V_{app}$, where V_0 is the built-in voltage, which refers to the voltage at which J_{ph} is zero, and V_{app} represents the applied voltage bias.[17]

1.7 Measurements of the hole and electron mobility by the SCLC method

The electron-only devices were fabricated with ITO/ZnO/active layer/ PDINN-S/Ag structures and hole-only devices were fabricated with ITO/2PACz/active layer/MoO_x/Ag structures. The SCLC mobility was calculated according to the Mott-Gurney square law: $J = 9\varepsilon_r\varepsilon_0\mu V^2/8L^3$,[18] where J is the current density, ε_r is the relative dielectric constant of the transport medium component, ε_0 is the vacuum permittivity (8.85419×10^{-12} $CV^{-1}m^{-1}$), μ is the electron or hole mobility, L is the thickness of active layer, and V is the effective voltage ($V = V_{appl} - V_{bi}$, here V_{appl} is the applied potential, and V_{bi} the built-in potential which results from the difference in the work function of the anode and the cathode).

1.8 TPV and TPC measurement

The lifetime of carriers can be measured by the transient photovoltage measurement. The background illumination was provided by a normal LED light source, and pulsed light was provided by arbitrary wave generator. The photovoltage traces were registered by the oscilloscope. The photocurrent traces were registered with the resistance of 50 Ω, switching open-circuit mode to short-circuit mode. The integrated TPC signal provides a measure of the total charge generated by the laser pulse (ΔQ).

1.9 TA spectroscopy measurements

For fs-TA spectroscopy, the fundamental output from Yb:KGW laser (1030 nm, 220 fs Gaussian fit, 100 kHz, Light Conversion Ltd) was separated into two light beams. One was introduced to NOPA (ORPHEUS-N, Light Conversion Ltd) to produce a certain wavelength for pump beam (here we use 550 and 750 nm, 30 fs pulse duration), the other was focused onto a YAG plate to generate white light continuum as the probe beam. The pump and probe overlapped on the sample at a small angle of less than 10°. The transmitted probe light from the sample was collected by a linear CCD array.

1.10 Morphology characterization

2D-GIWAXS data were measured on the XEUSS SAXS/WAXS equipment at National Center for Nanoscience and Technology (NCNST) of China. AFM images were obtained by Bruker Dimension Icon in the tapping mode.

1.11 AFM-IR measurements

An IR-neaSCOPE from neaspec was used to map IR absorption via detecting the mechanical response of an AFM cantilever upon sample stimulation by pulsed IR light. In the instrument, a pulsed tunable QCL laser is focused onto a PtIr coated AFM probe via a parabolic mirror, while the microscope is operated in the intermittent contact mode. The readout of the optically induced changes in the sample is performed by providing bimodal excitation of the cantilever and monitoring the response at the second cantilever Eigen-mode, while the first Eigen-mode is used for AFM topography and feedback. Such an active bimodal technique enables reliable tracking of the cantilever resonance, which in turn suppresses mechanically induced artifacts in the AFM-IR image contrasts. The pulse width of the QCL laser is in the ns range in tapping AFM-IR operation. The linewidth of the laser is typically about 1 cm^{-1}. The accessible spectral range depends on the selection of central wavelengths when equipping the laser. Up to 4 ranges can be selected and within those ranges, the laser is freely tunable. In this measurement mode, a resolution of ~1 cm^{-1} can be achieved within the tuning range of the laser.

2 Synthetic Section

2.1 General procedure for the synthesis of 2a and 2b

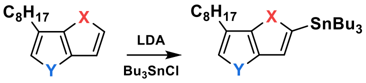

1a: X = S, Y = Se
1b: X = Se, Y = S
2a: X = S, Y = Se
2b: X = Se, Y = S

Compound **1** (898.05 mg, 3.00 mmol) was dissolved in dry THF (15 mL) under N_2 atmosphere. Then LDA (2.4 M in hexane, 1.25 mL, 3.00 mmol) was added dropwise slowly at −78 °C. After stirred 1 h, tributyltin chloride (1.07 g, 3.30 mmol) was added, and the reaction mixture was naturally heated to room temperature overnight. The resulting mixture was quenched by addition of water and extracted with dichloromethane (DCM); the organic layer was dried over anhydrous Na_2SO_4. The solvent was removed by rotary evaporation to give the products as a yellow oil, which was used without further purification.

Compound **2a** (1.64 g, 93%): ^{1}H NMR (500 MHz, $CDCl_3$, δ): 7.46 (s, 1H), 7.32 (s, 1H), 2.70 (t, *J* = 7.7 Hz, 2H), 1.80 – 1.75 (m, 2H), 1.63 – 1.58 (m, 6H), 1.39 – 1.35 (m, 10H), 1.32 – 1.28 (m, 6H), 1.17 – 1.13 (m, 6H), 1.93 – 1.90 (m, 12H).

Compound **2b** (1.1.59 g, 90%): ^{1}H NMR (500 MHz, $CDCl_3$, δ): 7.56 (s, 1H), 6.95 (s, 1H), 2.73 (t, *J* = 7.7 Hz, 2H), 1.78 – 1.73 (m, 2H), 1.63 – 1.59 (m, 6H), 1.39 – 1.35 (m, 10H), 1.31 – 1.28 (m, 6H), 1.16 – 1.12 (m, 6H), 0.95 – 0.91 (m, 12H).

2.2 General procedure for the synthesis of 3a and 3b

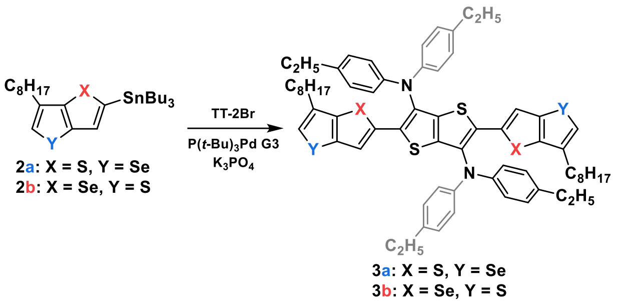

TT-2Br (372.3 mg, 0.50 mmol), compound **2** (735.5 mg, 1.25 mmol), P(*t*-Bu)$_3$Pd G3 (11.4 mg, 0.02 mmol), and K_3PO_4 (530.7 mg, 2.50 mmol) were dissolved in dry THF (15.0 mL). The tube was purged with nitrogen for 3 evacuate/refill cycles, and the reaction mixture was stirred at room temperature for 1 hour. The resulting reaction mixture was washed with water, followed by extraction with DCM. The residue was then subjected to silica gel column chromatography eluted with petroleum ether (PE)/DCM (9:1, v/v) to afford the desired product.

Compound **3a**: yellow solid (541.0 mg, 91.6%); ^{1}H NMR (500 MHz, $CDCl_3$, δ): 7.37 (s, 2H), 7.23 (s, 2H), 7.09 – 7.06 (m, 16H), 2.61 – 2.54 (m, 12H), 1.64 – 1.60 (m, 4H), 1.28 – 1.20 (m, 32H), 0.88 – 0.86 (m, 6H); ^{13}C NMR (126 MHz, $CDCl_3$, δ): 143.45, 142.16, 138.69, 137.24, 137.05, 135.29, 134.08, 132.90, 132.48, 128.68, 124.30, 121.75, 120.29, 32.09, 31.57, 29.58, 29.51, 29.42, 28.49, 28.36, 27.11, 22.87, 15.66, 14.34.

Compound **3b**: yellow oil (545.2 mg, 92.3%); ^{1}H NMR (500 MHz, $CDCl_3$, δ): 7.37 (s, 2H), 7.11 – 7.09 (m, 16H), 6.84 (s, 2H), 2.62 – 2.58 (m, 12H), 1.64 – 1.60 (m, 4H), 1.27 – 1.21 (m, 32H), 0.89 – 0.87 (m, 6H); ^{13}C NMR (126 MHz, $CDCl_3$, δ): 143.42, 140.78, 139.17, 138.95, 138.05, 137.43, 135.90, 133.83, 132.59, 128.68, 121.74, 121.38, 118.82, 32.05, 30.74, 29.87, 29.53, 29.45, 29.36, 28.76, 28.33, 27.58, 22.83, 15.60, 14.30, 13.91, 8.91.

2.3 General procedure for the synthesis of 4a and 4b

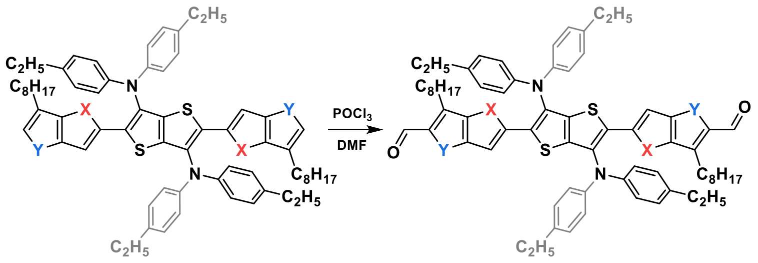

Compound **3** (472.6 mg, 0.40 mmol) was dissolved in dry DMF (10.0 mL). The tube was purged with nitrogen for 3 evacuate/refill cycles, $POCl_3$ (1 mL) was then dropwise to above solution, and the reaction mixture was stirred at 90 °C overnight. After cooling to room temperature, the resulting reaction mixture was washed with water, followed by extraction with DCM. The residue was then subjected to silica gel column chromatography eluted with PE/DCM (1:2, v/v) to afford the desired product as an orange solid.

Compound **4a** (423.7 mg, 85.6%): ^{1}H NMR (500 MHz, $CDCl_3$, δ): 9.89 (s, 2H), 7.20 (s, 2H), 7.11 – 7.06 (m, 16H), 2.93 (t, J = 7.6 Hz, 4H), 2.60 (q, J = 7.6 Hz, 8H), 1.68 – 1.63 (m, 4H), 1.28 – 1.19 (m, 32H), 0.89 – 0.86 (m, 6H). ^{13}C NMR (126 MHz, $CDCl_3$, δ): 182.92, 147.73, 143.22, 143.10, 143.05, 142.62, 140.84, 139.37, 135.17, 134.21, 131.60, 128.75, 121.96, 120.53, 31.93, 30.03, 29.81, 29.75, 29.50, 29.40, 29.22, 28.28, 22.72, 15.57, 14.21.

Compound **4b** (416.8 mg, 84.2%): ^{1}H NMR (500 MHz, $CDCl_3$, δ): 9.99 (s, 2H), 7.36 (s, 2H), 7.12 – 7.07 (m, 16H), 2.95 (t, J = 7.6 Hz, 4H), 2.62 (q, J = 7.6 Hz, 8H), 1.68 – 1.64 (m, 4H), 1.27 – 1.21 (m, 32H), 0.88 – 0.85 (m, 6H). ^{13}C NMR (126 MHz, $CDCl_3$, δ): 181.99, 147.85, 145.69, 143.51, 143.04, 141.72, 139.63, 138.09, 135.18, 135.07, 134.44, 128.83, 121.89, 118.78, 31.93, 30.09, 29.82, 29.45, 29.38, 29.20, 29.07, 28.29, 22.73, 15.55, 14.22.

2.4 General procedure for the synthesis of 3TT-SSe and 3TT-SeS

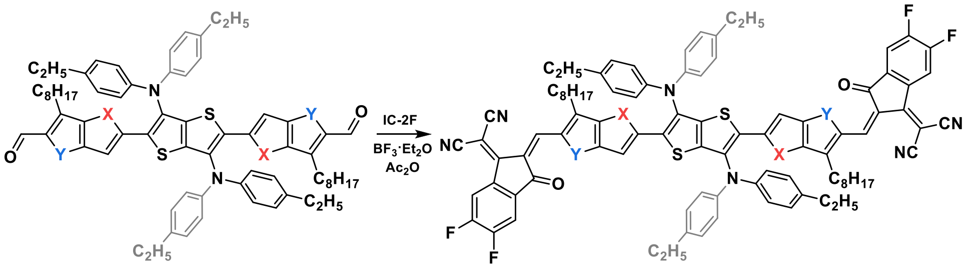

Compound **4** (371.3 mg, 0.30 mmol), 2-(5,6-difluoro-3-oxo-2,3-dihydro-1*H*-inden-1-ylidene)malononitrile (IC-2F, 151.8 mg, 0.66 mmol), acetic anhydride (0.1 mL) were dissolved in dry toluene (15.0 mL). Then $BF_3 \cdot Et_2O$ (0.5 mL) was added dropwise to the above solution and the reaction mixture was stirred at room temperature. After 30 mins, the mixture was concentrated to 5 mL and poured into methanol (50.0 mL) and then filtered. The residue was then subjected to silica gel column chromatography eluted with PE/DCM (1:1.5, v/v) to afford the desired product as a dark blue solid.

3TT-SSe (426.8 mg, 85.6%): ^{1}H NMR (500 MHz, $CDCl_3$, δ): 9.06 (s, 2H), 8.52 – 8.47 (m, 2H), 7.64 – 7.59 (t, *J* = 7.5 Hz, 2H), 7.29 – 7.27 (m, 2H), 7.14 – 7.11 (m, 8H), 7.10 – 7.07 (m, 8H), 2.95 (t, *J* = 7.8 Hz, 4H), 2.62 (q, *J* = 7.6 Hz, 8H), 1.60 – 1.57 (m, 4H), 1.26 – 1.22 (m, 32H), 0.88 – 0.86 (m, 6H). ^{13}C NMR (126 MHz, $CDCl_3$, δ): 186.38, 158.99, 155.45, 153.42, 153.35, 145.09, 144.77, 143.03, 139.95, 137.90, 136.66, 136.51, 135.84, 135.59, 134.56, 134.51, 131.96, 128.87, 122.24, 120.74, 119.71, 114.96, 114.93, 114.79, 114.58, 112.57, 112.42, 68.68, 31.95, 31.88, 31.49, 30.85, 29.71, 29.67, 29.39, 29.36, 29.12, 28.26, 22.68, 22.62, 15.39, 14.05. ^{19}F NMR (471 MHz, $CDCl_3$, δ): -123.0 – -123.2 (m), -124.3 – -124.4 (m). HR-MS (MALDI-TOF) *m/z* calcd. for $C_{92}H_{78}F_4N_6O_2S_4Se_2$: 1662.3336; found: 1662.4104.

3TT-SeS (429.3 mg, 86.1%): ^{1}H NMR (500 MHz, $CDCl_3$, δ): 8.97 (s, 2H), 8.54 – 8.49 (m, 2H), 7.66 – 7.62 (m, 2H), 7.42 – 7.39 (m, 2H), 7.16 – 7.13 (m, 8H), 7.11 – 7.08 (m, 8H), 2.98 (t, *J* = 7.6 Hz, 4H), 2.63 (q, *J* = 7.6 Hz, 8H), 1.67 – 1.62 (m, 4H), 1.27 – 1.22 (m, 32H), 0.88 – 0.85 (m, 6H). ^{13}C NMR (126 MHz, $CDCl_3$, δ): 185.86, 159.04, 155.41, 155.32, 155.29, 153.42, 153.34, 147.29, 142.92, 140.17, 136.55, 136.49, 136.04, 135.63, 135.12, 134.55, 134.49, 134.27, 128.98, 122.13, 122.05, 120.20, 118.76, 115.01, 114.91, 114.72, 112.60, 112.45, 68.57, 31.95, 30.93, 30.85, 29.80, 29.62, 29.43, 29.16, 28.32, 22.72, 15.56, 14.22. ^{19}F NMR (471 MHz, $CDCl_3$, δ): -123.1 – -123.2 (m), -124.2 – -124.4 (m). HR-MS (MALDI-TOF) *m/z* calcd. for $C_{92}H_{78}F_4N_6O_2S_4Se_2$: 1662.3336; found: 1662.4381.

3 Supplementary Figures

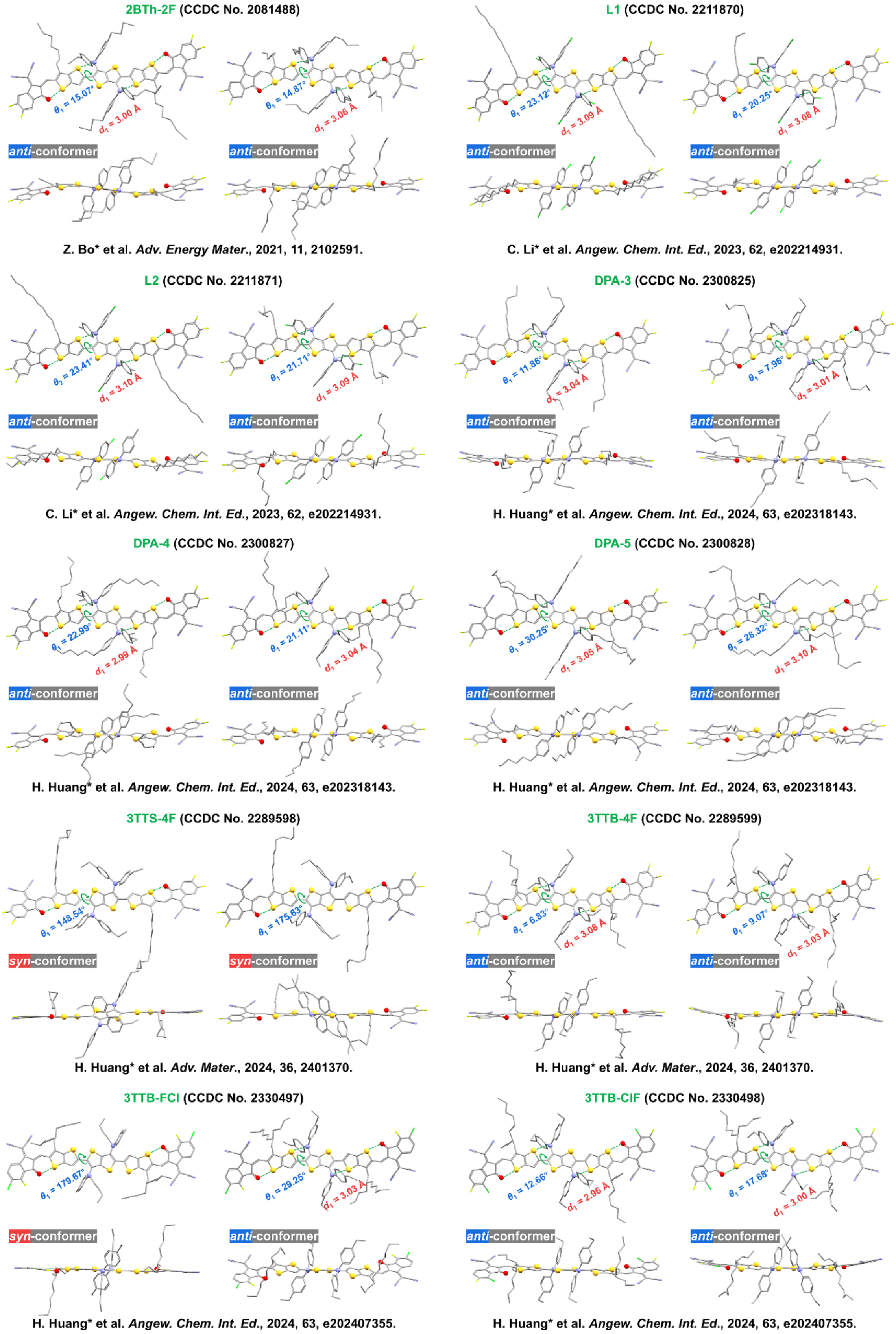

Figure S1. Crystallographic statistics. Crystal structures of conformational isomers for 3TT-series NFREAs reported in literatures.

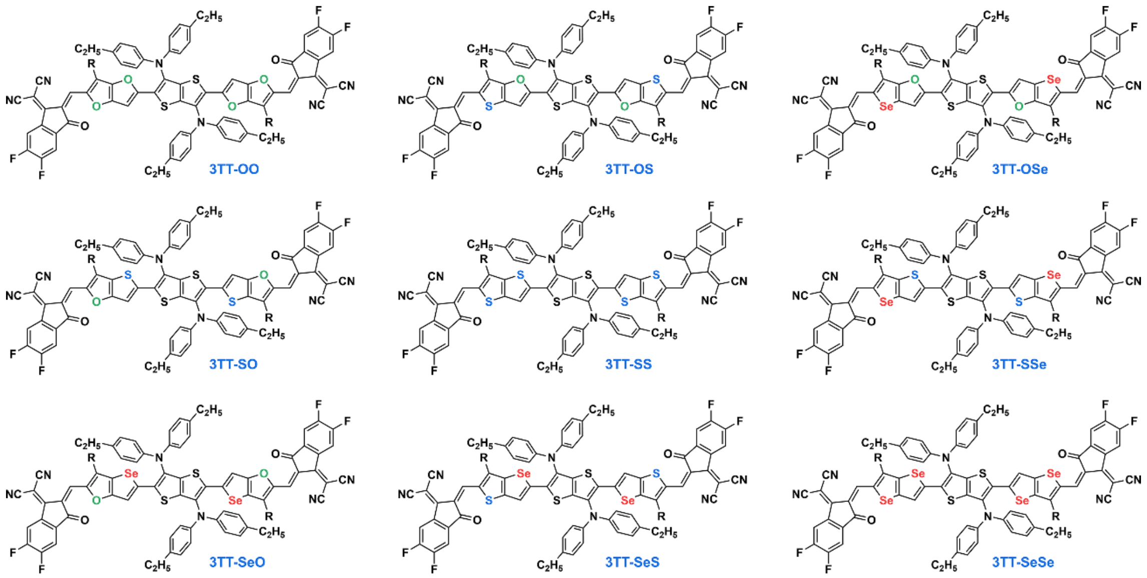

Figure S2. Model molecular design. Chemical structures of model NFREAs featuring diverse chalcogens (oxygen, sulfur, and selenium).

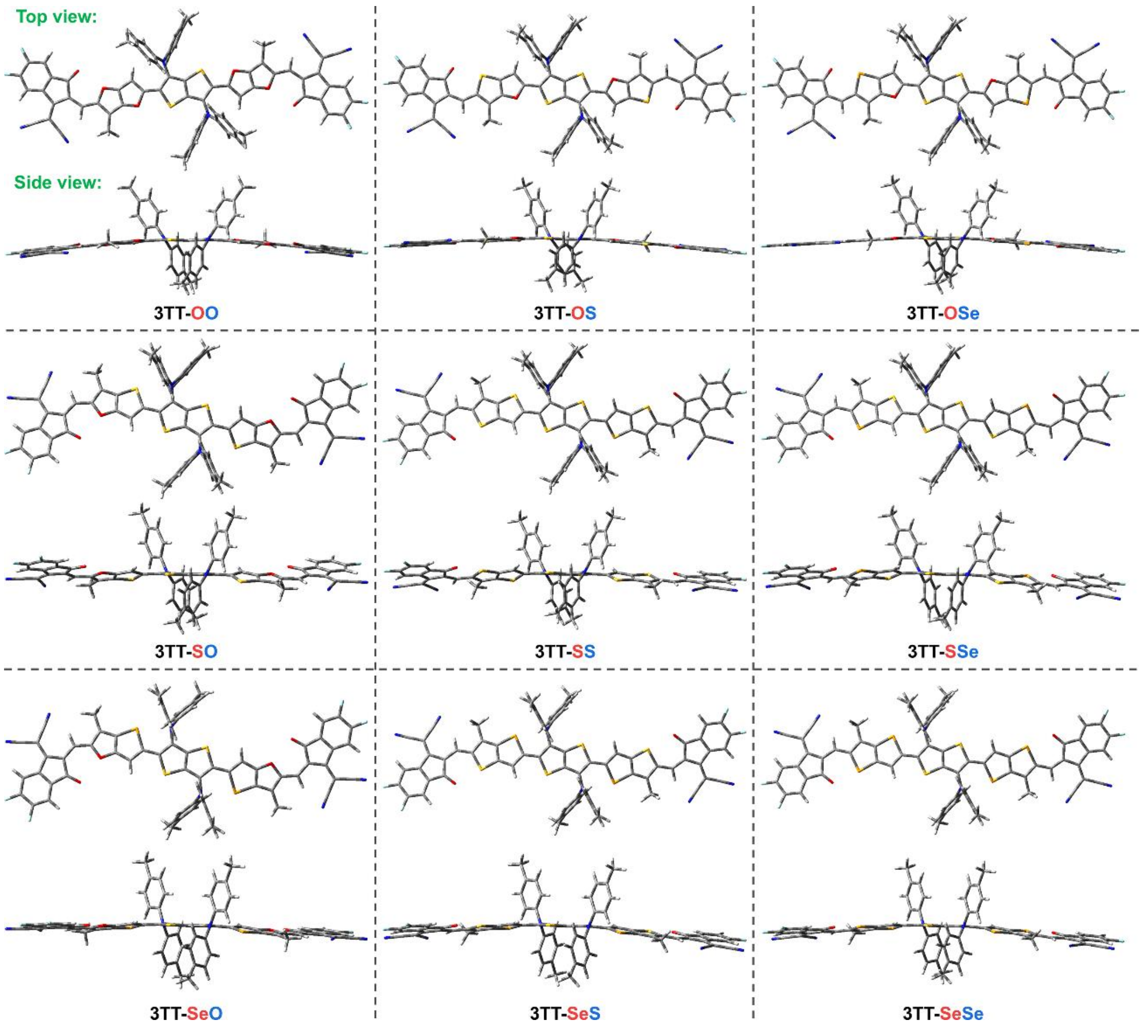

Figure S3. Molecular geometry. Optimal geometries of model NFREAs by DFT calculations.

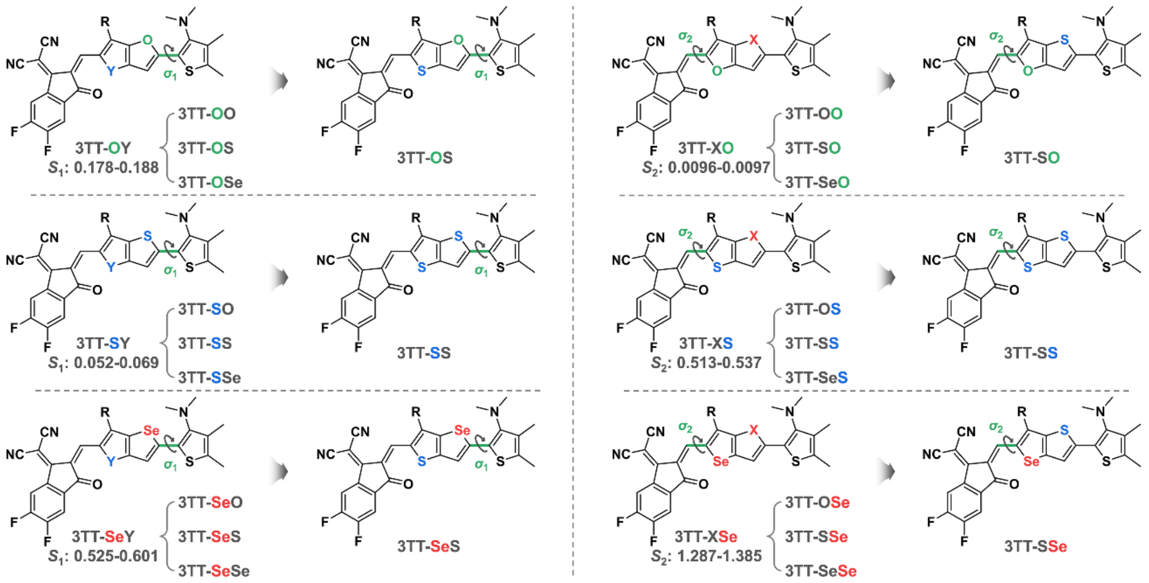

Figure S4. Calculated *S* value and selected representative models. To enable comparative analysis, 3TT-XS and 3TT-SY were selected as representatives to investigate the rotational dynamics of single bonds σ_1 and σ_2, respectively.

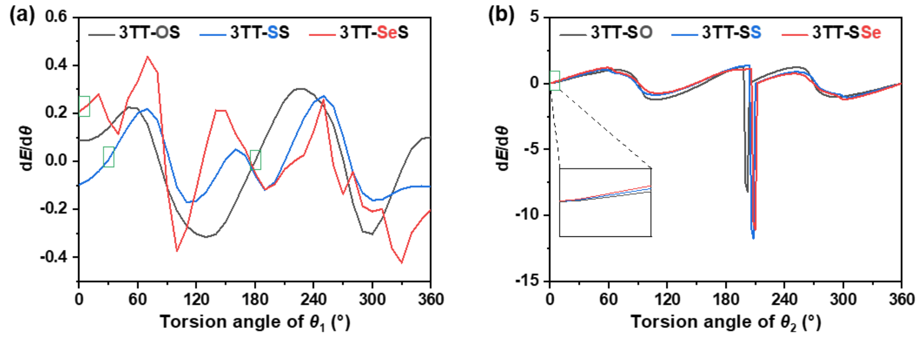

Figure S5. Quantify the steepness of the potential energy curve. The first derivative of the potential energy curves.

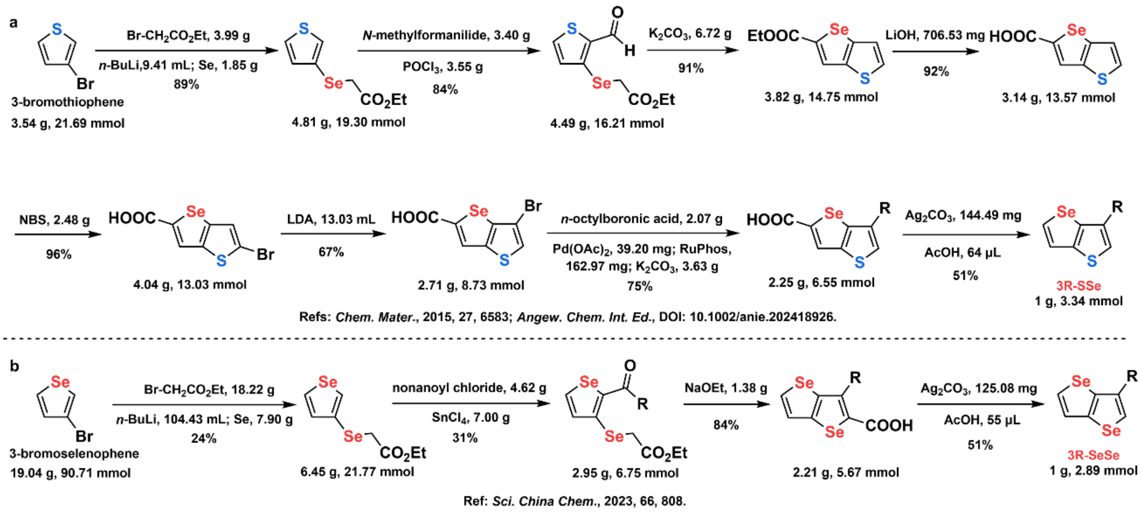

Figure S6. Synthetic route for MOC calculations. The detailed synthetic routes and yields of for 3R-SeS (**a**) and 3R-SeSe (**b**).

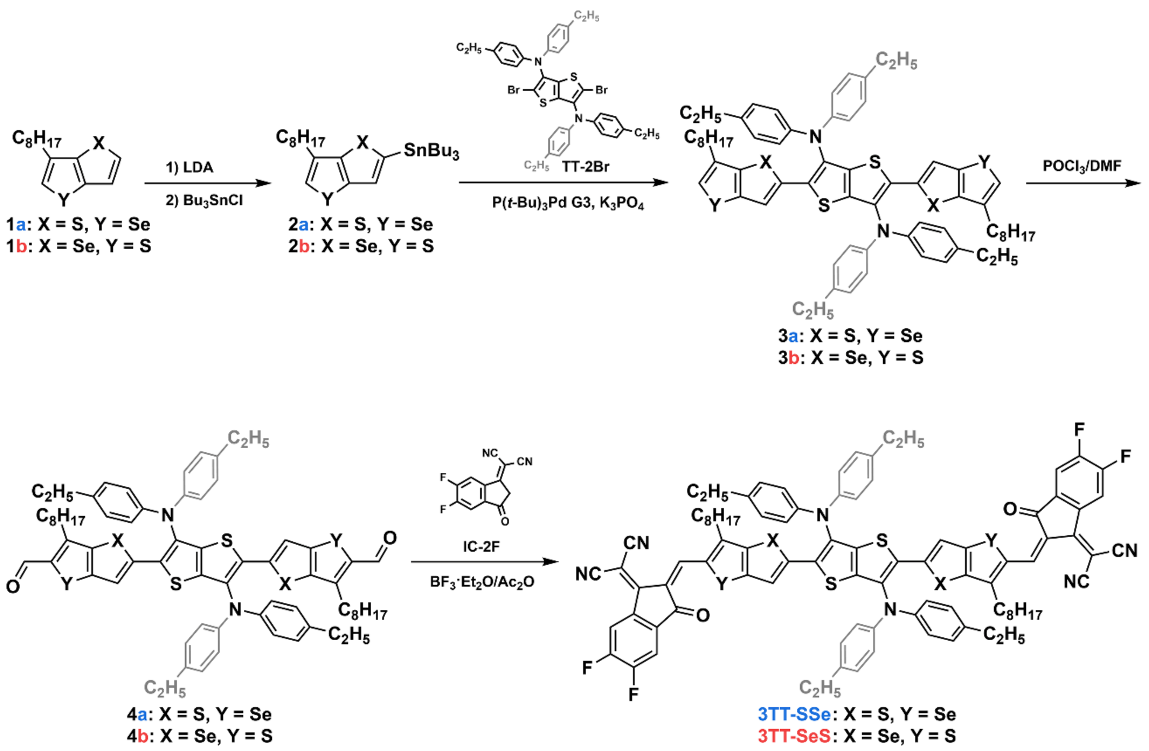

Figure S7. Synthetic route toward 3TT-SSe and 3TT-SeS. Compound 1 and TT-2Br was synthesized according to the reported methods[19, 20]. Detailed characterizations of the key intermediates and final products are shown in Supplementary Figures 50-66.

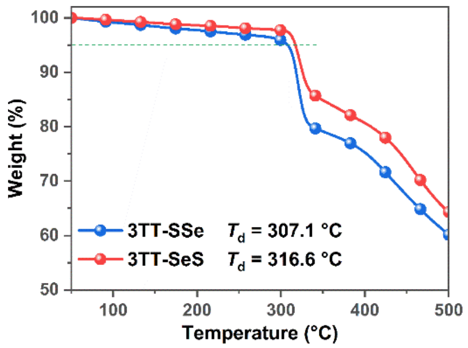

Figure S8. Thermogravimetric analysis. TGA traces of 3TT-SSe and 3TT-SeS recorded at a heating rate of 10 °C min^{-1} under a N_2 atmosphere.

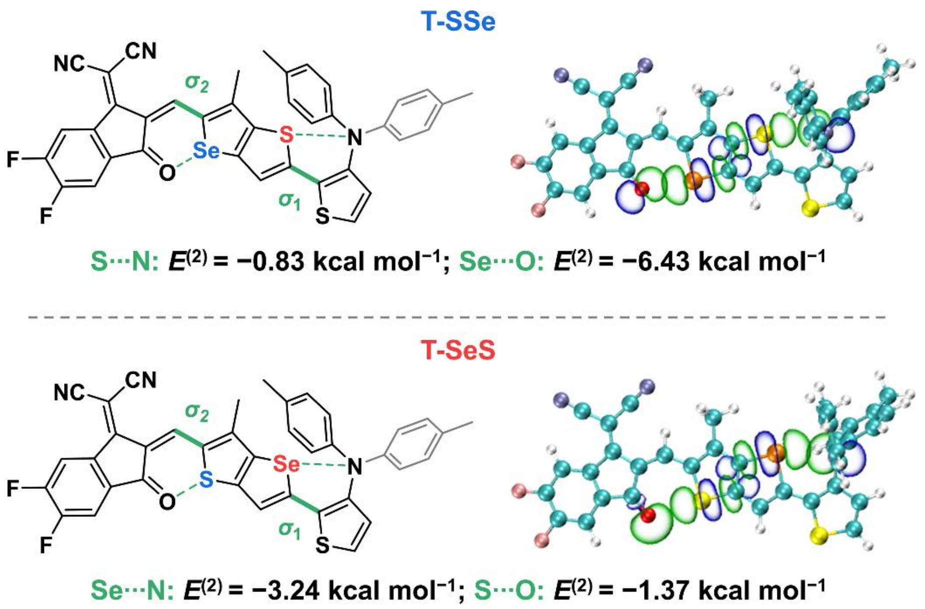

Figure S9. **Natural bond orbital analysis.** Orbital overlap diagrams and second-order perturbative energies ($E^{(2)}$). The semi-structural models (T-SSe and T-SeS) were extracted from parent systems (3TT-SSe and 3TT-SeS) to simplify computational analysis.

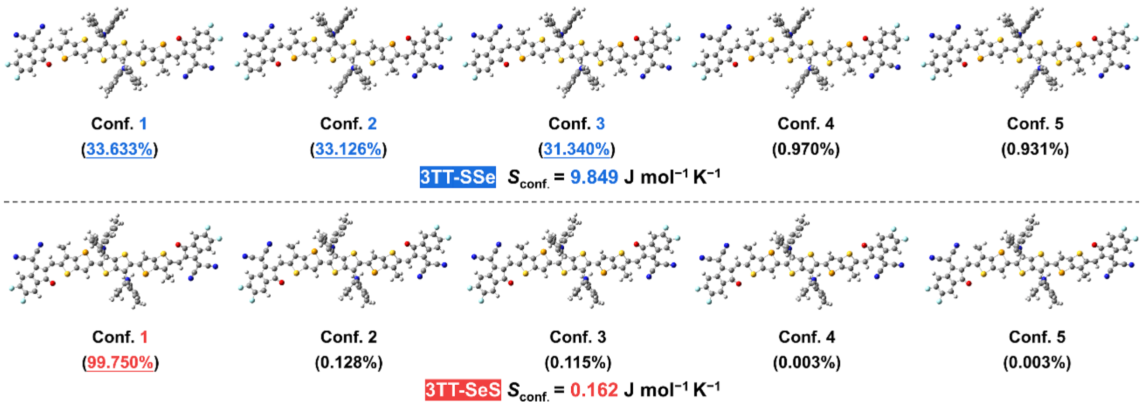

Figure S10. Conformer population. Geometry of the top-five conformers screened by conformation search program.

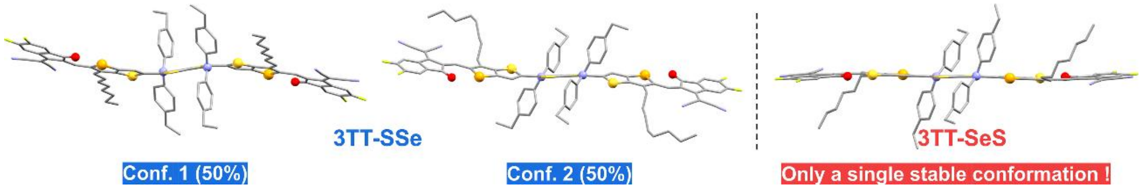

Figure S11. Crystal structures. Side-view representations for 3TT-SSe and 3TT-SeS.

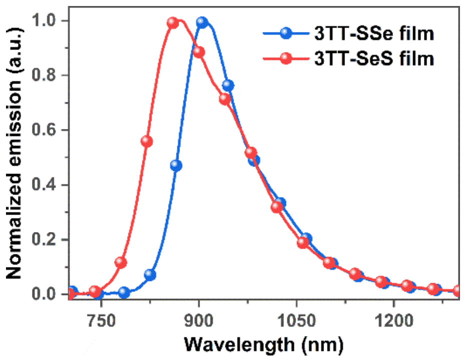

Figure S12. Emission Property. PL spectra of 3TT-SSe and 3TT-SeS in thin films.

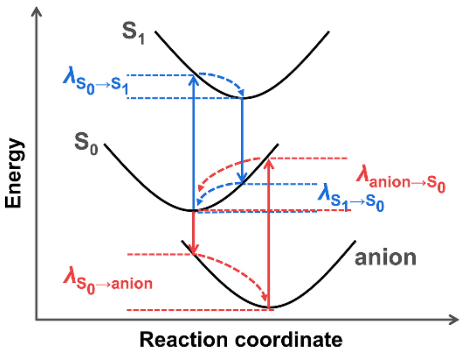

$$\lambda_{EET} = \lambda_{S_0 \rightarrow S_1} + \lambda_{S_1 \rightarrow S_0}$$

$$\lambda_{e} = \lambda_{S_0 \rightarrow anion} + \lambda_{anion \rightarrow S_0}$$

Figure S13. Reorganization energies calculation. Illustration of the related transitions among the ground state (S_0), the lowest singlet excited state (S_1), and the anion state during photoelectric conversion processes, by taking the electron acceptor as an example.

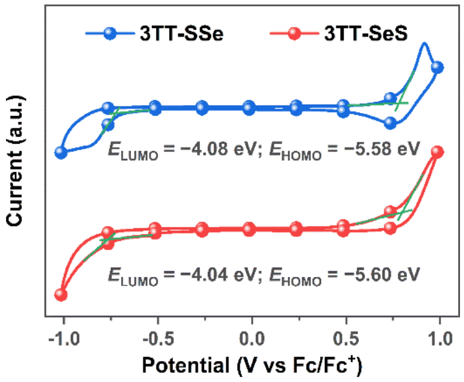

Figure S14. Electrochemical characterization. CV plots of both acceptors measured in 0.1 M [(*n*-Bu)$_4$N]$^+$PF$_6^-$ acetonitrile solution at a scan rate of 100 mV s^{-1}.

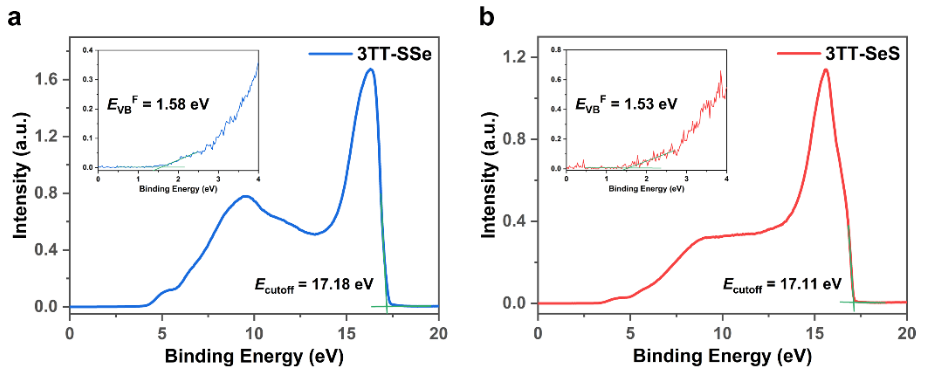

Figure S15. UPS spectra. UPS spectra (He I: 21.22 eV) of the onset (inset) and the secondary edge region of 3TT-SSe (a) and 3TT-SeS (b).

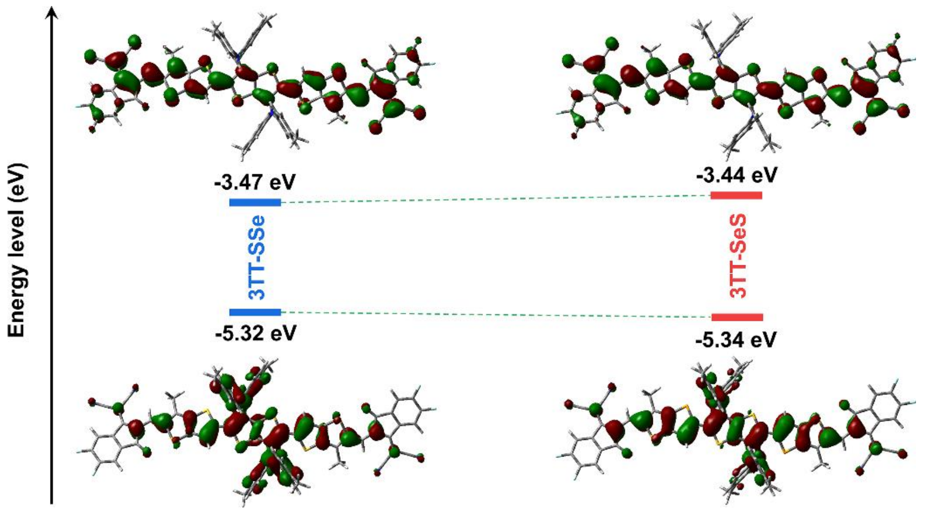

Figure S16. DFT-calculated energy levels. Charge density distributions of HOMO/LUMO and their energy levels.

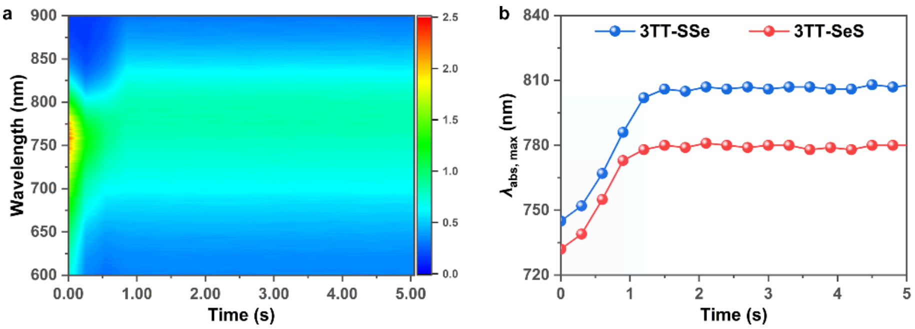

Figure S17. In-situ UV–vis absorption spectra of neat films. a, Time-dependent contour maps of in-situ UV–vis absorption spectra for 3TT-SSe film. **b**, Time evolution of the maximum absorption peak locations of the 3TT-SSe and 3TT-SeS films.

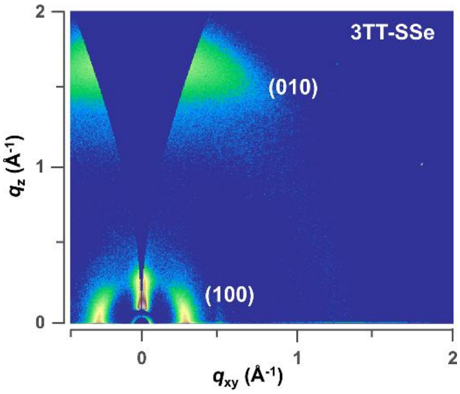

Figure S18. Near-surface micro-structure. 2D-GIWAXS pattern of the neat 3TT-SSe film.

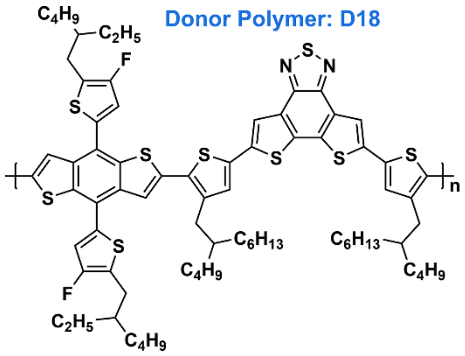

Figure S19. Donor material. Chemical structure of polymer donor D18.

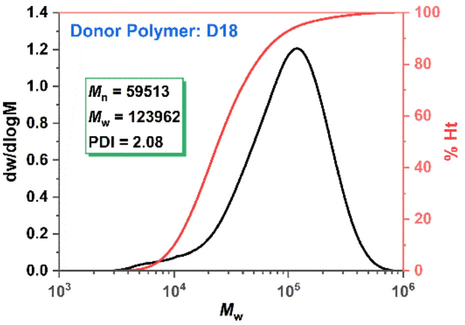

Figure S20. Molecular weight and polydispersity analysis. GPC trace of polymer donor D18.

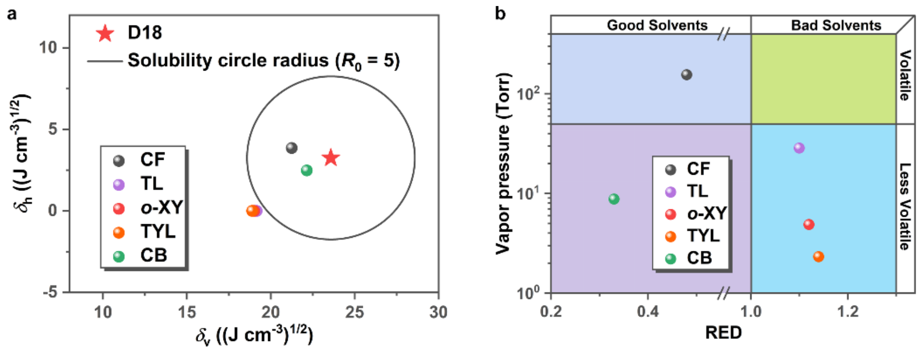

Figure S20. Solvent selection. a, The solubility of D18 in various solvents in the δ_v–δ_h diagram, where δ_h is molecular hydrogen bonding interactions, δ_v is calculated using the following equation: $\delta v = \sqrt{\delta_D^2 - \delta_P^2}$ (δ_D: atomic dispersive interactions; δ_P: permanent dipole molecular interactions). **b**, Solvent classification diagram based on vapour pressure and solubility. The above data is from ref. [21].

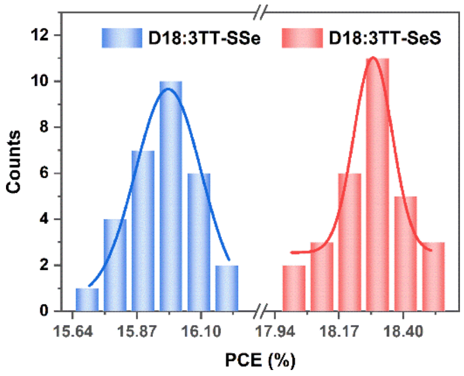

Figure S22. Photovoltaic efficiency statistics. Histogram of the PCE measurements for 30 devices based on D18:3TT-SSe and D18:3TT-SeS, fitted with Gaussian distributions (solid lines).

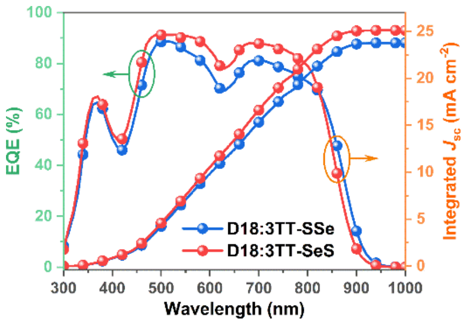

Figure S23. EQE response. EQE spectra for optimized D18:3TT-SSe and D18:3TT-SeS devices.

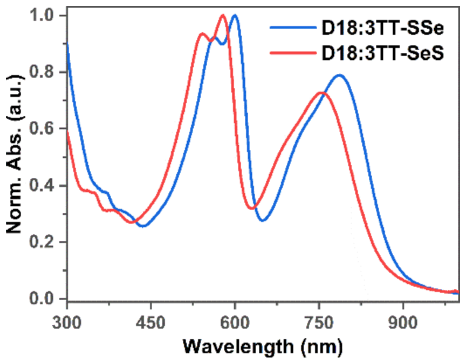

Figure S24. Absorption spectra. UV–vis absorption spectra of the D18:3TT-SSe and D18:3TT-SeS blend films.

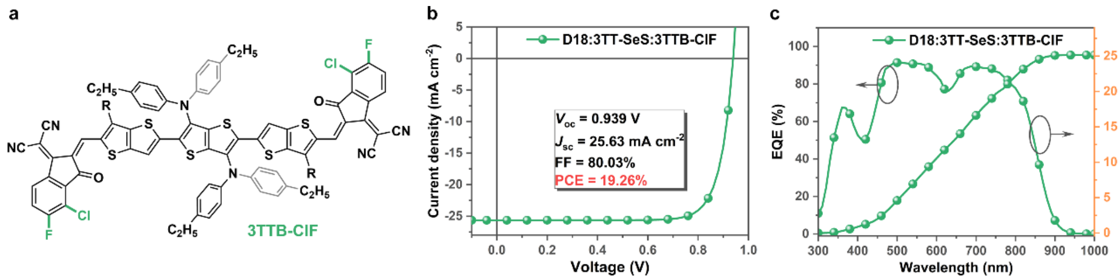

Figure S25. Ternary photovoltaic device. a, The chemical structure of 3TTB-ClF. *J*–*V* curve (**b**)and EQE response (**c**) of the D18:3TT-SeS:3TTB-ClF based OSC.

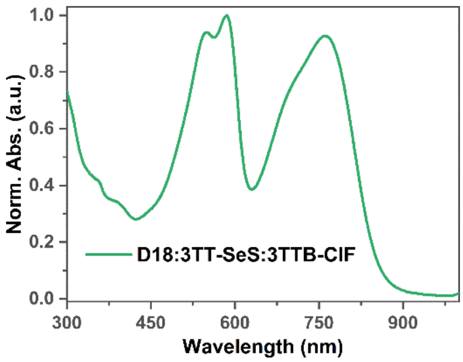

Figure S26. Absorption spectra. UV–vis absorption spectra of the D18:3TT-SeS:3TTB-ClF blend film.

福建省计量科学研究院
FUJIAN METROLOGY INSTITUTE
（国家光伏产业计量测试中心）
National PV Industry Measurement and Testing Center

检 测 报 告

Test Report

报告编号：24Q3-00731
Report No.

客户信息 Name of Custome	中国科学院大学（University of Chinese Academy of Sciences）
联络信息 Contact Information	北京市怀柔区雁栖湖东路1号（Huairou District, Beijing 101408, P. R. China）
物品名称 Name of Items	有机太阳能电池（Organic Solar Cell）(IV)
型号/规格 Type /Specification	聚合物-非富勒烯（Polymer-Non-fullerene based）
物品编号 Items No	45
制造厂商 Manufacturer	中国科学院大学（University of Chinese Academy of Sciences）
物品接收日期 Items Receipt Date	2024-11-11
检测日期 Test Date	2024-11-11

（Stamp）

批准人：黎健生 Approved by

核验员：何翔 Checked by

检测员：陈彩云 Test by

发布日期：2024 年 11 月 18 日
Date of Report Year Month Day

扫一扫 查真伪

本院/本中心地址：福州市屏东路9-3号
Address: 9-3 Pingdong Road,Fuzhou,China
电话：0591-87845050 Telephone
传真：0591-87808417 Fax
邮编：350003 Post Code
网址：www.fjjl.net Web Site
咨询电话：0591-87845050 Inquire line
投诉电话：0591-87823025 Complaint Tel

未经本院/本中心书面批准，部分采用本报告内容无效。
Partly using this Report will not be admitted unless allowed by FMI/ Center.

检测结果/说明：
Results of Test and additional explanation.

1. 标准测试条件 Standard Test Condition (STC)：

总辐照度 Total Irradiance：1000 W/m^2
被测电池温度 Temperature：25.0 °C
光谱分布 Spectral Distribution：AM1.5G

2.STC条件下测量数据 Measurement Data and I-V/P-V Curves under STC

正扫 Forward Scan

I_{sc} (mA)	V_{oc}(V)	I_{MPP}(mA)	V_{MPP}(V)	P_{MPP} (mW)	FF(%)	A (cm^2)
0.7759	0.9378	0.7274	0.7963	0.5792	79.60	0.0309

反扫 Reverse Scan

I_{sc} (mA)	V_{oc}(V)	I_{MPP}(mA)	V_{MPP}(V)	P_{MPP} (mW)	FF(%)	A (cm^2)
0.7753	0.9379	0.7270	0.7963	0.5789	79.61	0.0309

失配因子 Mismatch factor: 0.9944

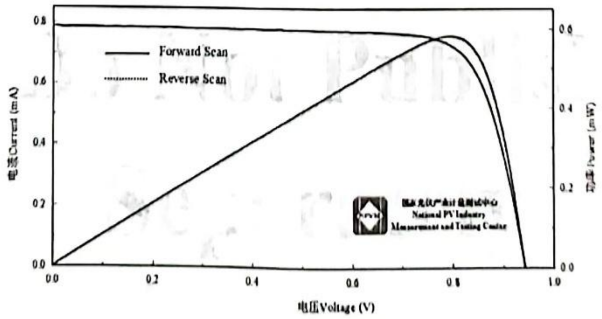

图1 STC下电流-电压特性曲线和功率-电压特性曲线
Figure 1. I-V and P-V characteristic curves of the measured sample under STC

检测结果/说明：
Results of Test and additional explanation.

3.STC条件下最大功率稳态输出的测量数据及曲线Measurement Data and Curves for MPPT under STC

η (%)	18.65
P_{MPP} (mW)	0.5763
I_{MPP} (mA)	0.7305
V_{MPP} (V)	0.7889

说明：上表为 300秒测试过程中最后 30 秒内 STC 条件下的最大功率稳态输出平均测试数据
Note: Measurement data for MPPT under STC in the above table was the mean value acquired during the final 30 seconds of the 300 seconds test

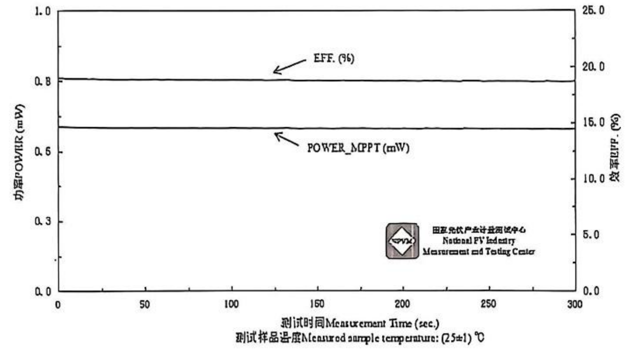

图2 被测样品最大功率稳态输出的功率-效率曲线
Figure 2. Measurement curves of the measured sample for MPPT

福建省计量科学研究院
FUJIAN METROLOGY INSTITUTE
（国家光伏产业计量测试中心）
National PV Industry Measurement and Testing Center

报告编号： 24Q3-00731
Report No.

检测结果/说明：
Results of Test and additional explanation.

4.Pictures of the Measured Sample

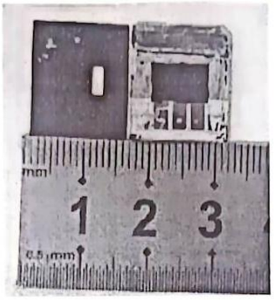

图3 被测样品的正面图像及测试中使用的掩膜板
Figure/

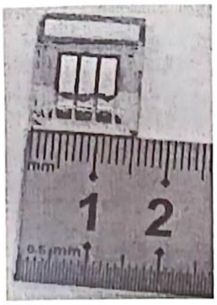

图4 被测样品的反面图像
Figure 4. Reverse side of the measured sample

测量结果的不确定度为/Uncertainty of measurement results:
短路电流/Short-Circuit Current: U_{rel}=1.8 % (k=2); 开路电压/Open-Circuit Voltage: U_{rel}=1.0 % (k
最大功率/Maximum Power: U_{rel}=2.2 % (k=2); 转换效率/Efficiency: U_{rel}=2.2 % (k=2);
填充因子/Fill Factor: U_{rel}=3.2 % (k=2).
说明：该报告中的面积为限定辐照面积。
Explanation： The measured area refers to designated illuminated area.

检测报告续页专用
Continued page of test report

第 6 页/共 7 页
Page of Pages

Figure S27. Certification report. The certification report obtained from the National Photovoltaic Industry Metrology and Testing Center (NPVM), China.

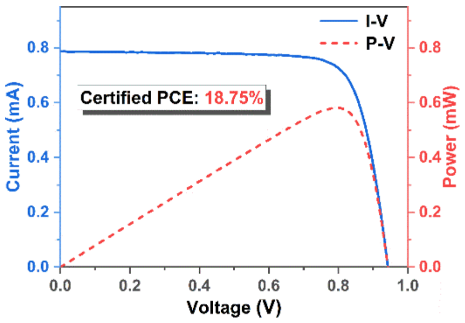

Figure S28. Certified results. *I–V* characteristic for the optimized D18:3TT-SeS-based OSCs.

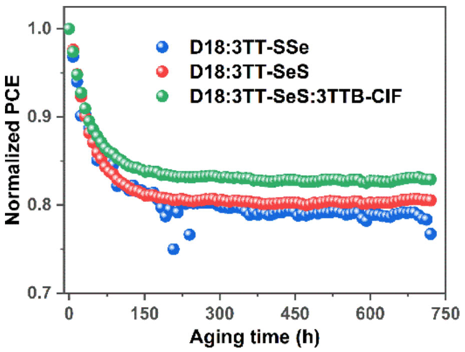

Figure S29. Operational stability. Device stability of encapsulated devices under continuous one-sun illumination (AM 1.5G).

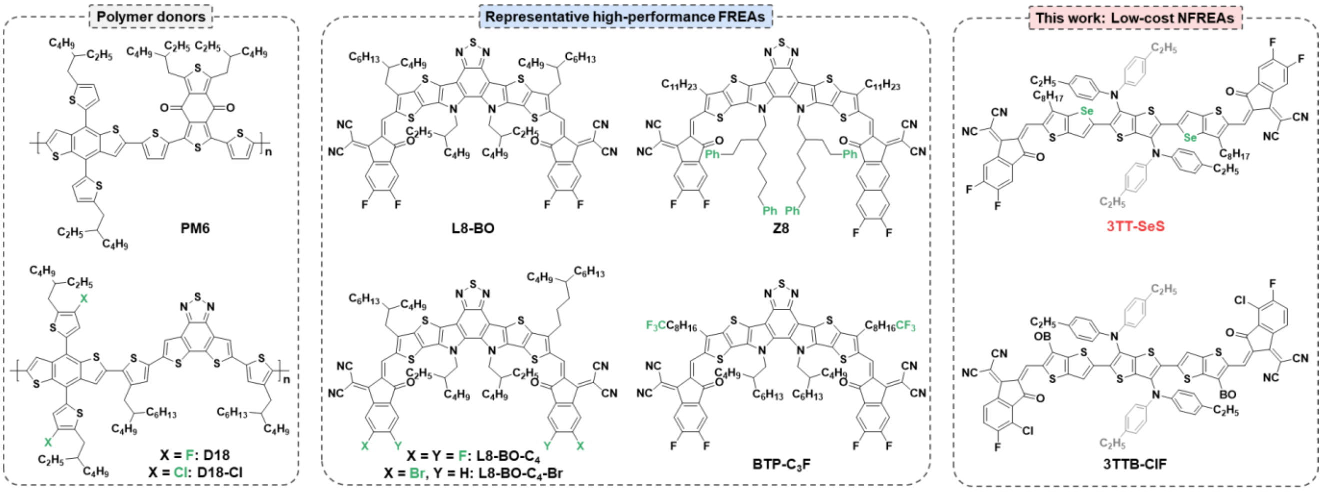

Figure S30. Chemical structures. Molecular structures of representative donors and acceptors for cost calculation.

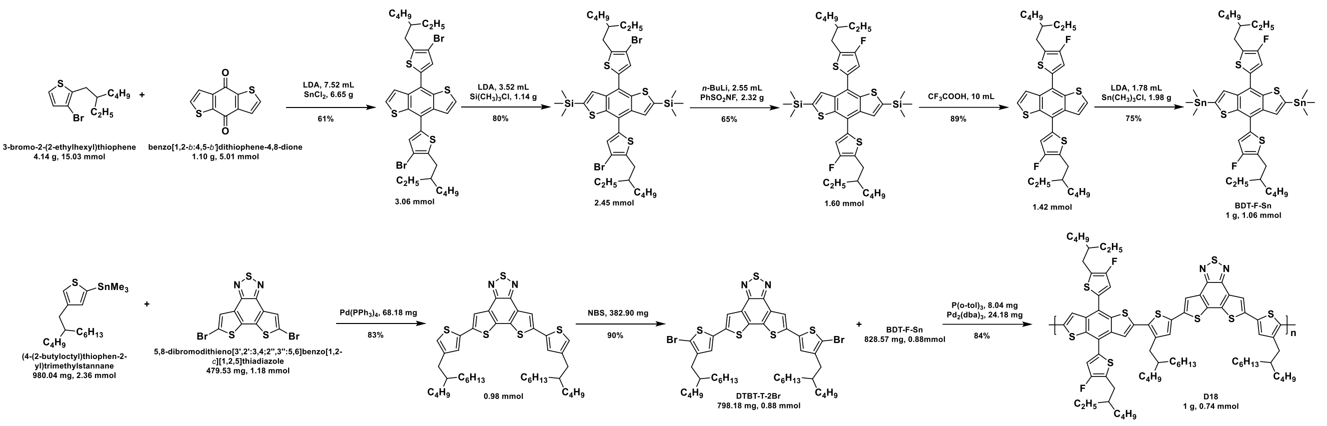

Figure S31. Synthetic route for MOC calculations. The detailed synthetic routes and yields for D18[22-24].

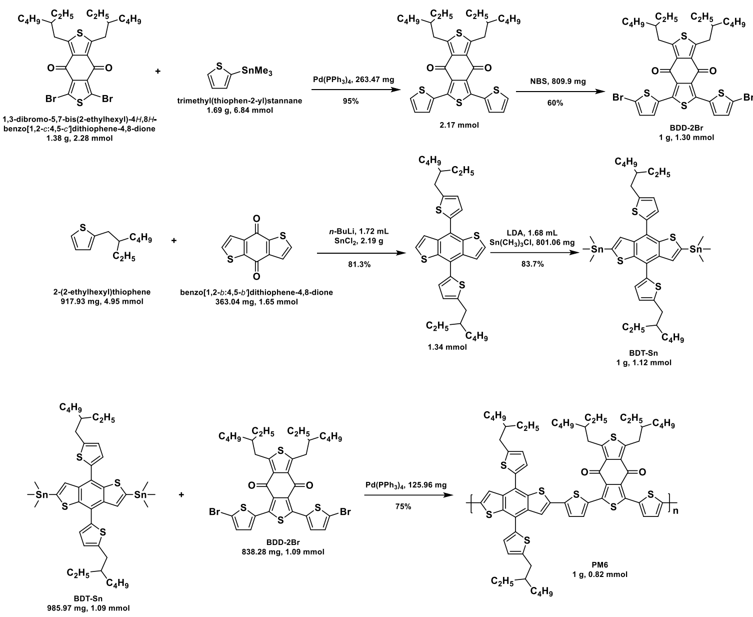

Figure S32. The detailed synthetic routes and yields for PM6[25].

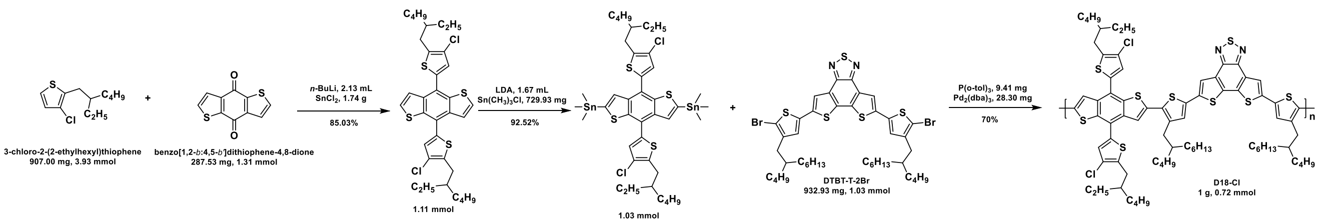

Figure S33. Synthetic route for MOC calculations. The detailed synthetic routes and yields for D18-Cl[26, 27].

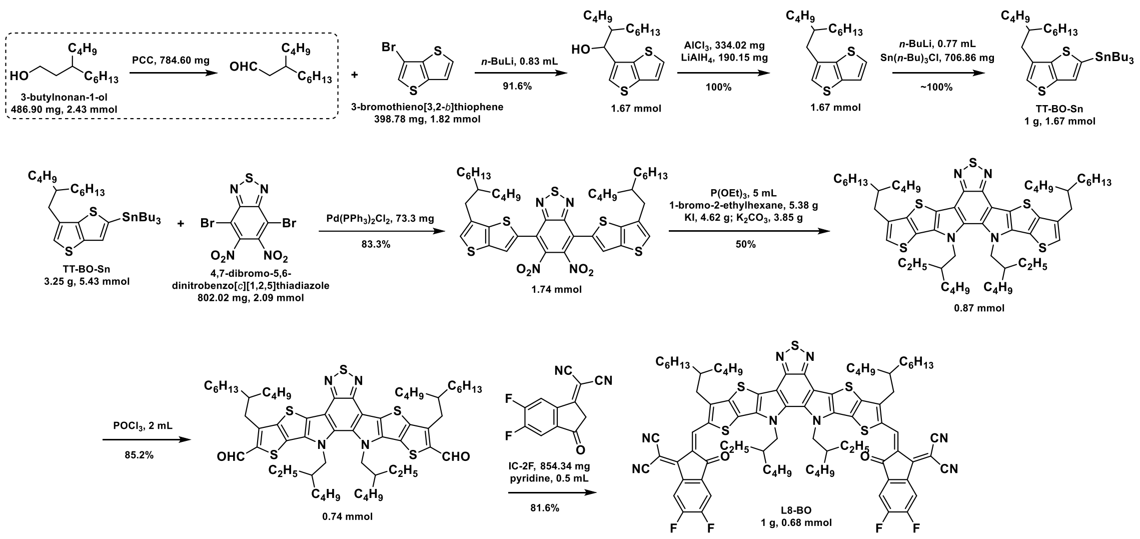

Figure S34. **Synthetic route for MOC calculations.** The detailed synthetic routes and yields for L8-BO[28].

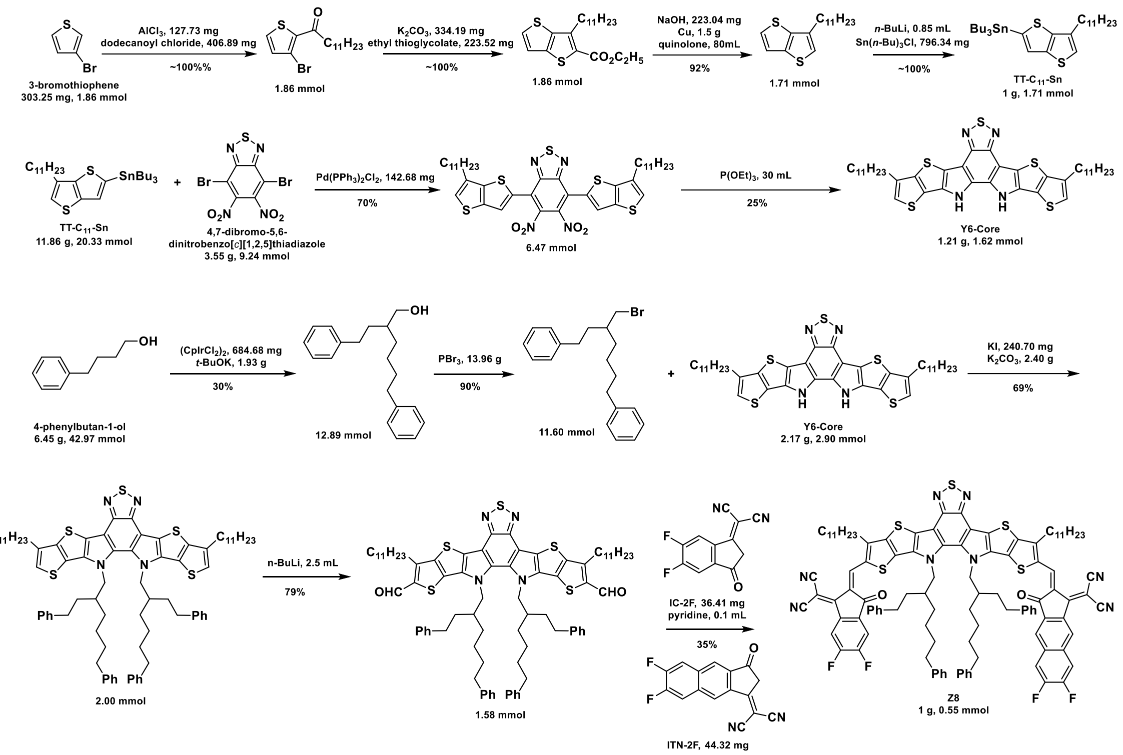

Figure S35. **Synthetic route for MOC calculations.** The detailed synthetic routes and yields for Z8[29, 30].

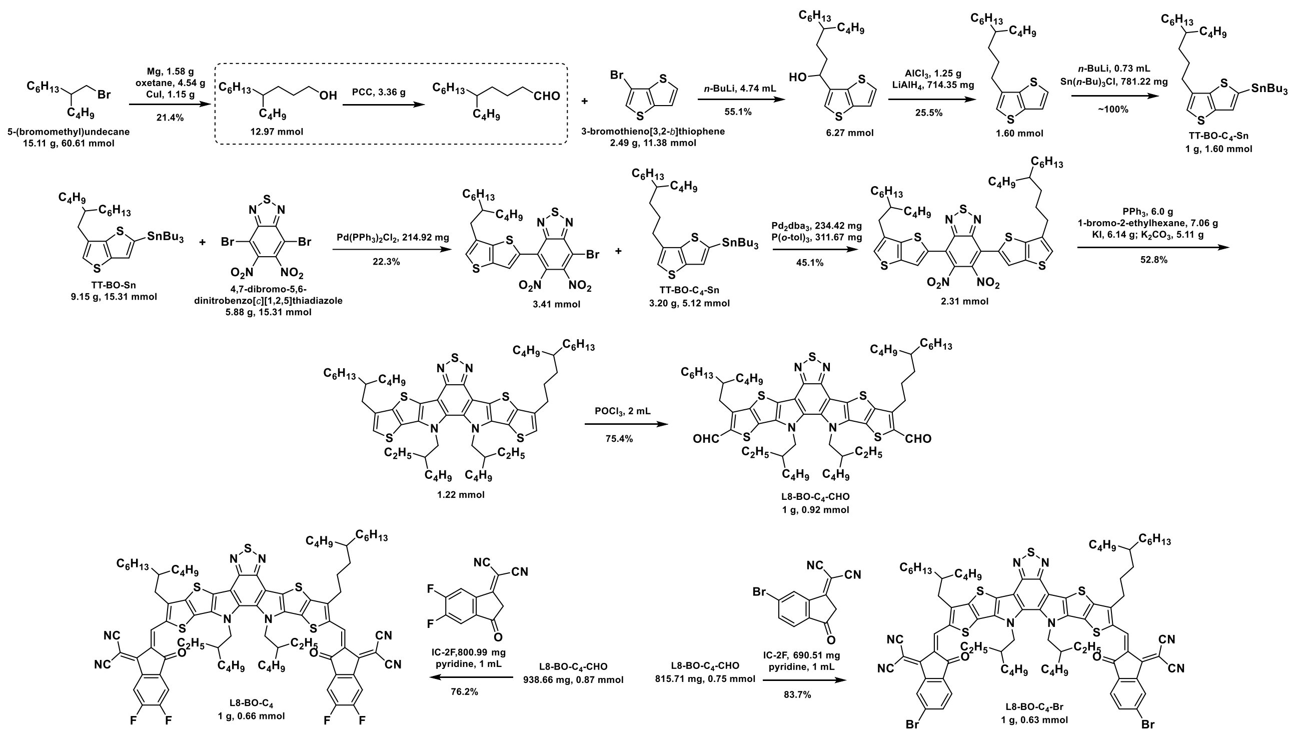

Figure S36. **Synthetic route for MOC calculations.** The detailed synthetic routes and yields for L8-BO-C_4 and L8-BO-C_4-Br[31].

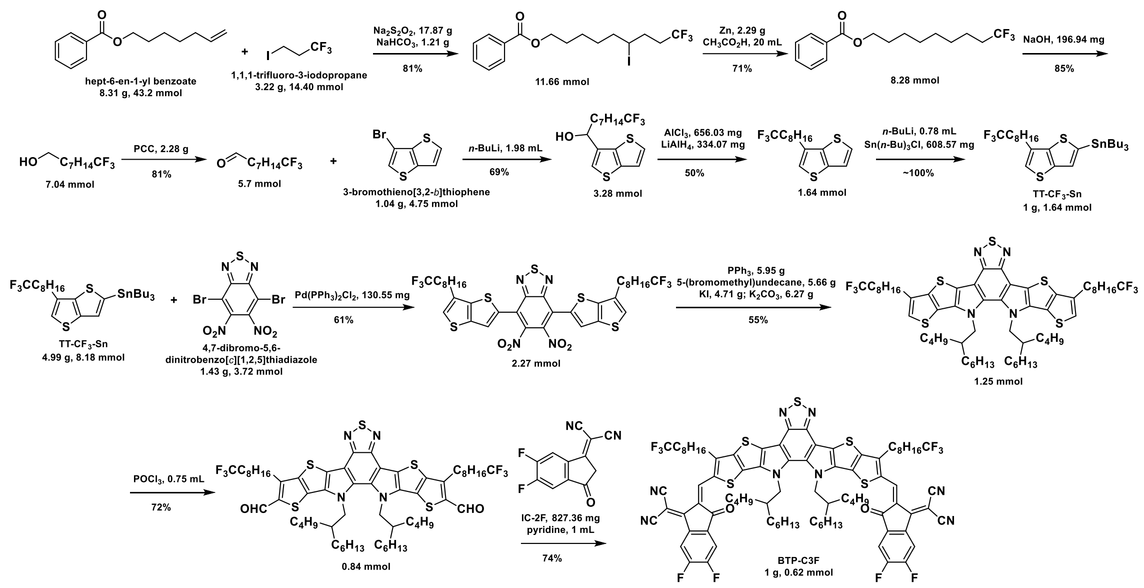

Figure S37. **Synthetic route for MOC calculations.** The detailed synthetic routes and yields for BTP-C3F[32].

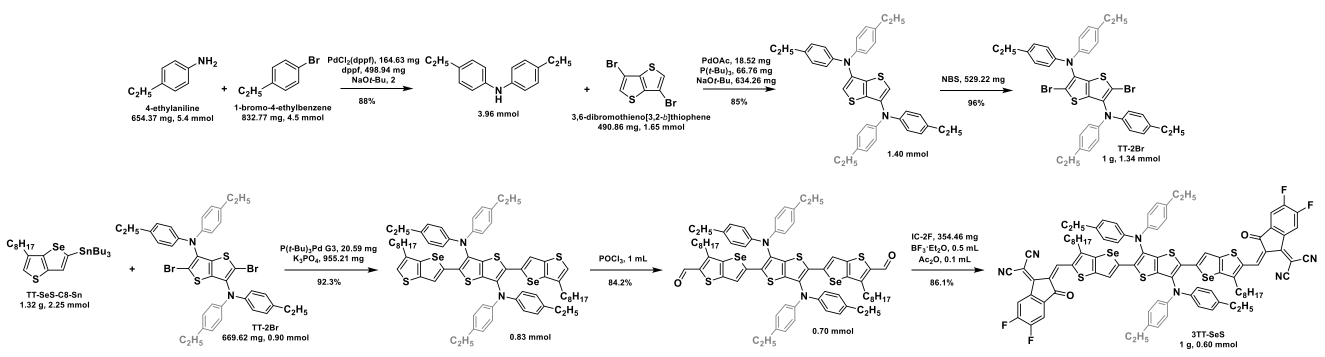

Figure S38. **Synthetic route for MOC calculations.** The detailed synthetic routes and yields for 3TT-SeS[19, 20].

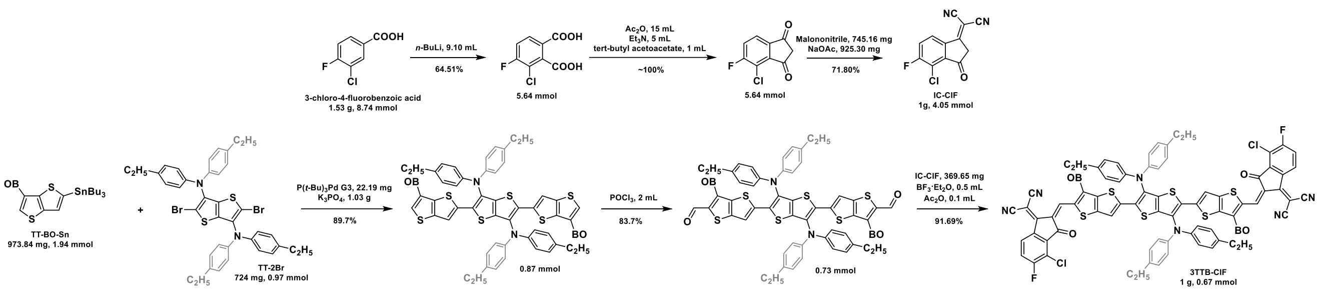

Figure S39. **Synthetic route for MOC calculations.** The detailed synthetic routes and yields for 3TTB-ClF[33, 34].

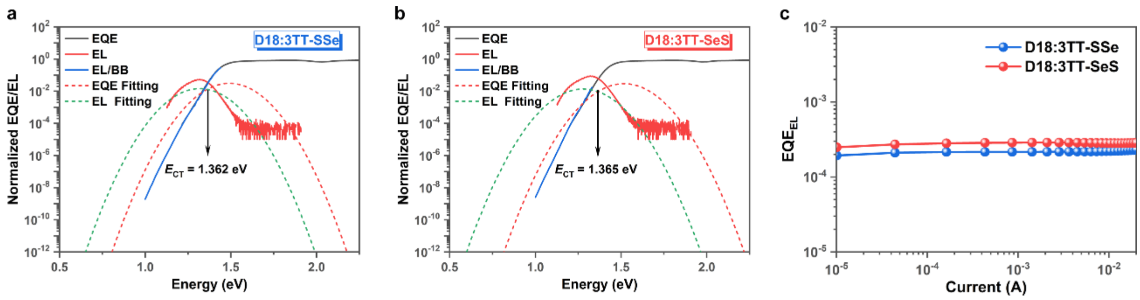

Figure S40. Energy loss analysis in OSCs. Semilogarithmic plots of normalized EL and normalized FTPS-EQE (solid lines), and of reciprocally calculated EL and EQE (dashed lines) as a function of energy for OSC devices based on D18:3TT-SSe (**a**) and D18:3TT-SeS (**b**). **c**, EQE_{EL} curves of the two optimized devices.

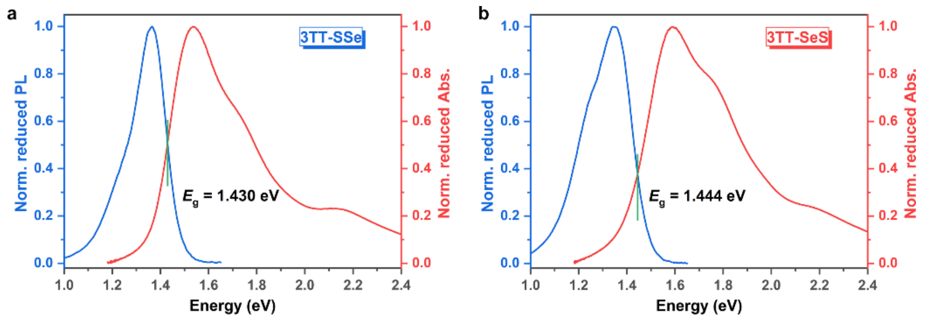

Figure S41. E_g calculation. Normalized PL and absorption spectra of neat acceptor films.

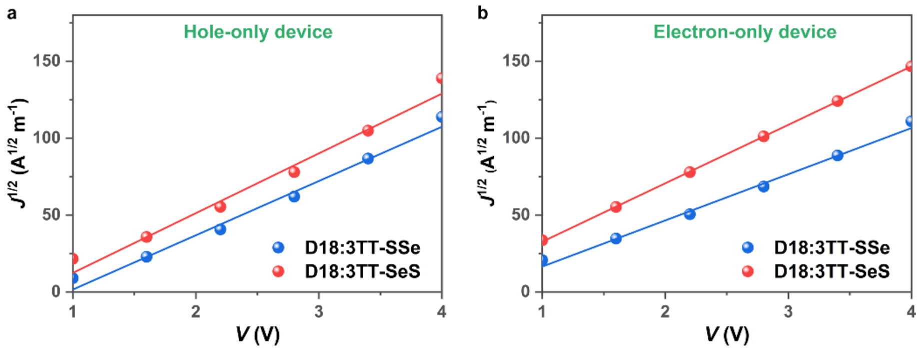

Figure S42. Carrier mobilities. SCLC curves of hole-only (**a**) and electron-only (**b**) devices.

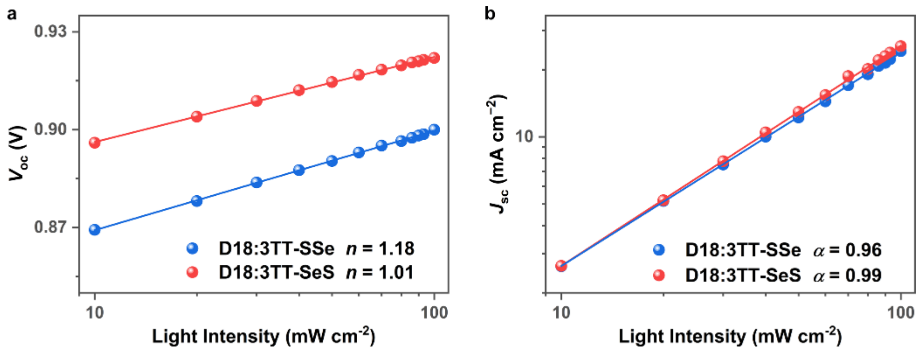

Figure S43. Light-intensity dependences of V_{oc} and J_{sc}. P_{light}-dependent V_{oc} (**a**) and J_{sc} (**b**) plots of D18:3TT-SSe- and D18:3TT-SeS-based devices.

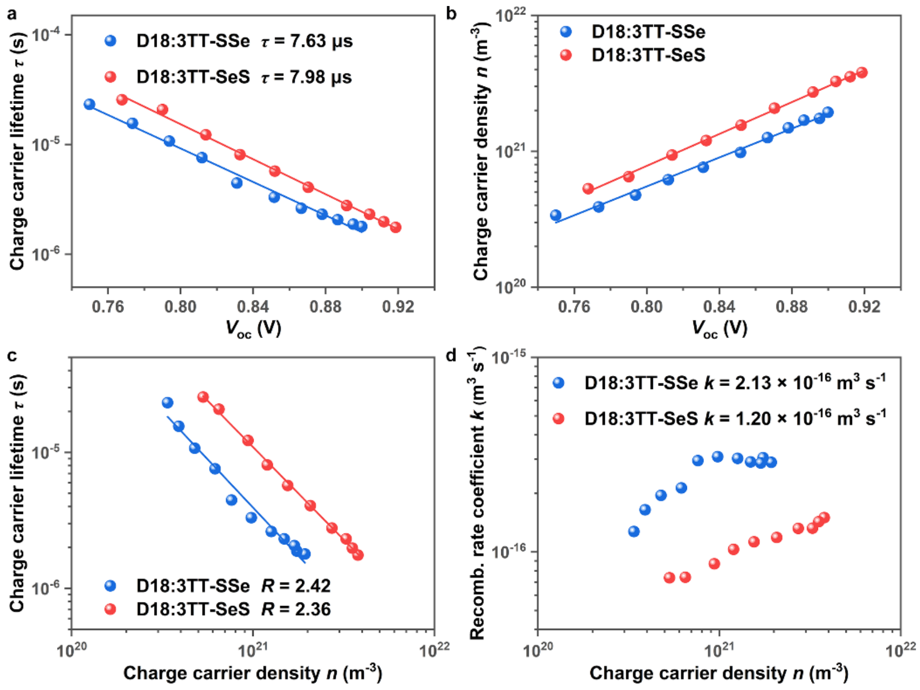

Figure S44. TPV and TPC measurements. The extracted charge carrier lifetime (τ) (**a**) and charge carrier density (n) (**b**) as a function of V_{oc} under different bias light intensities. **c,** Charge carrier lifetime (**c**) and recombination rate coefficient (k) (**d**) as a function of the carrier density.

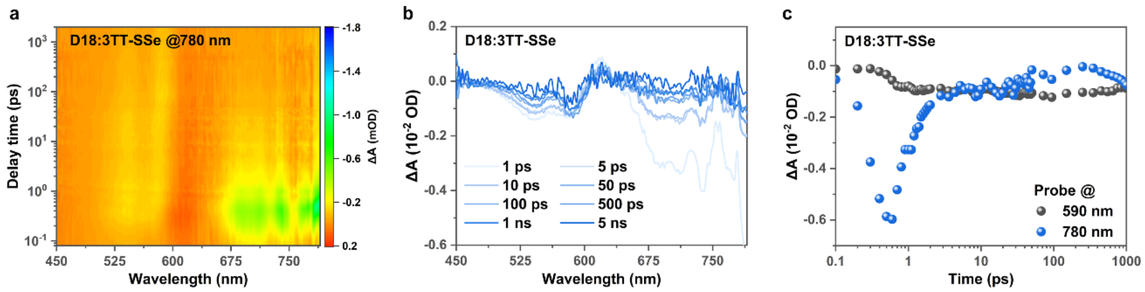

Figure S45. Ultrafast absorption spectroscopy. 2D color plots of TA spectra (**a**), representative TA spectra recorded at indicated delay times (**b**), and TA kinetic probed at 590 nm and 780 nm (**c**) for the 3TT-SSe-based blend film.

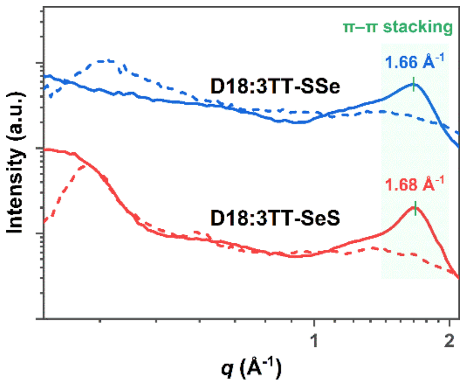

Figure S46. Molecular stacking behavior in blend films. 2D-GIWAXS scattering profiles in the in-plane (dashed lines) and out-of-plane (solid lines) directions.

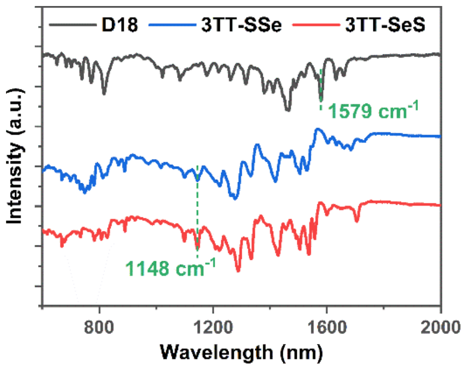

Figure S47. FT-IR spectra of D18, 3TT-SSe, and 3TT-SeS.

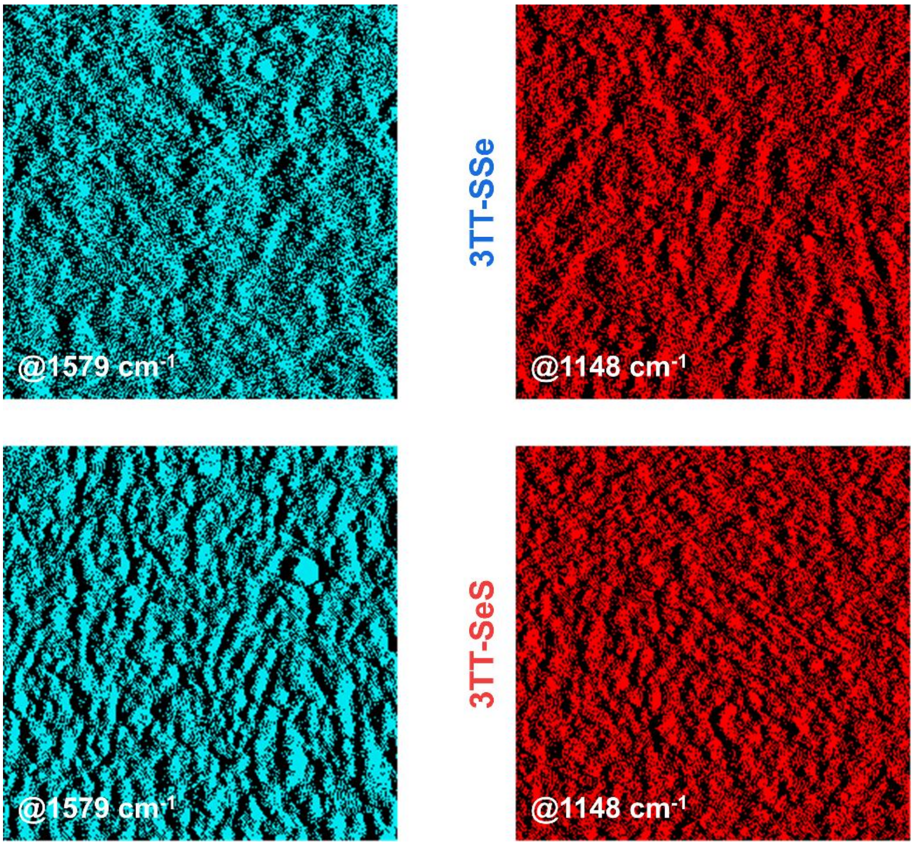

Figure S48. AFM-IR characterization. AFM-IR images at the wavenumber of 1579 cm^{-1} (representing D18) and 1148cm^{-1} (representing acceptor).

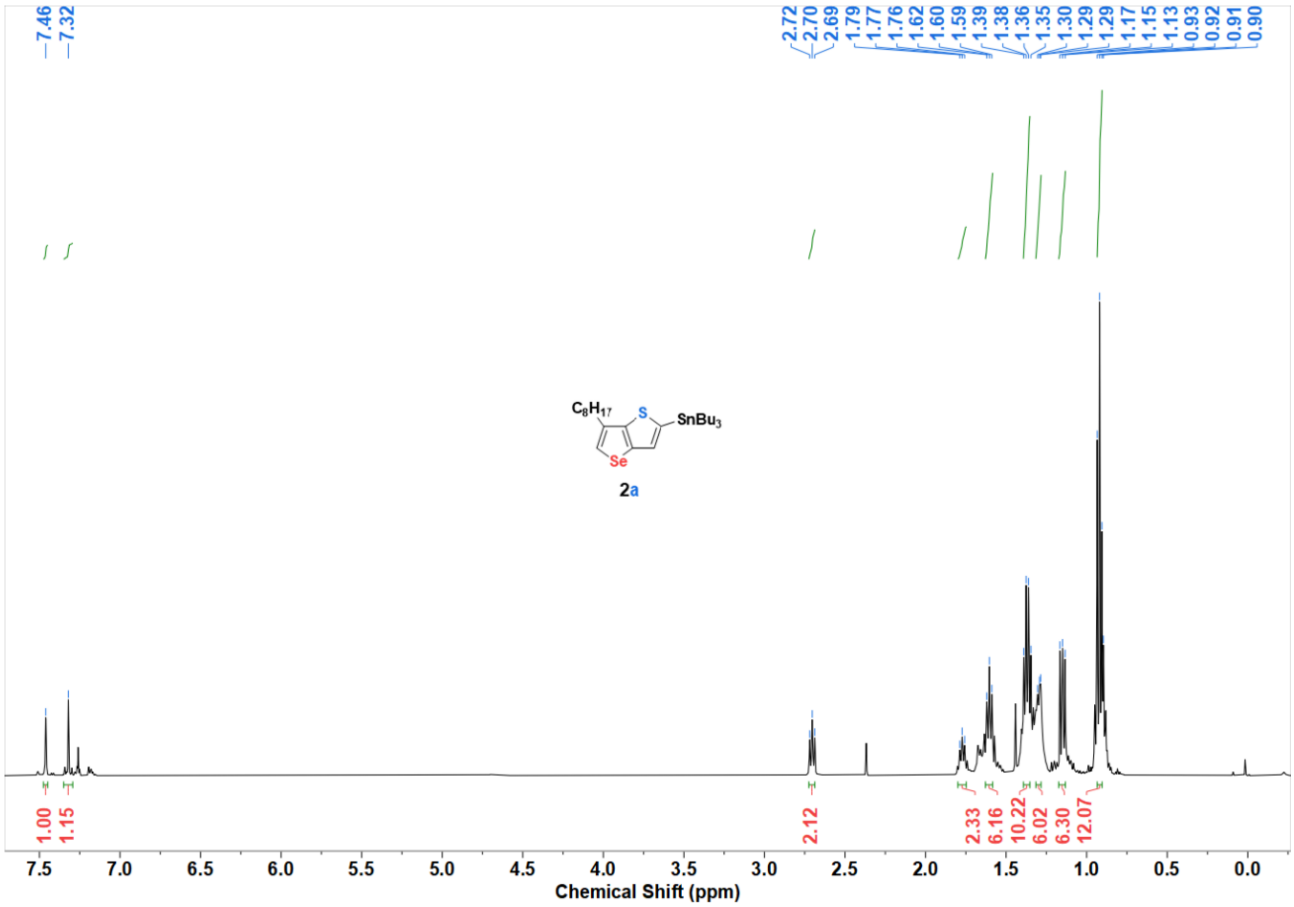

Figure S49. ^{1}H NMR (500 MHz) spectrum of compound 2a.

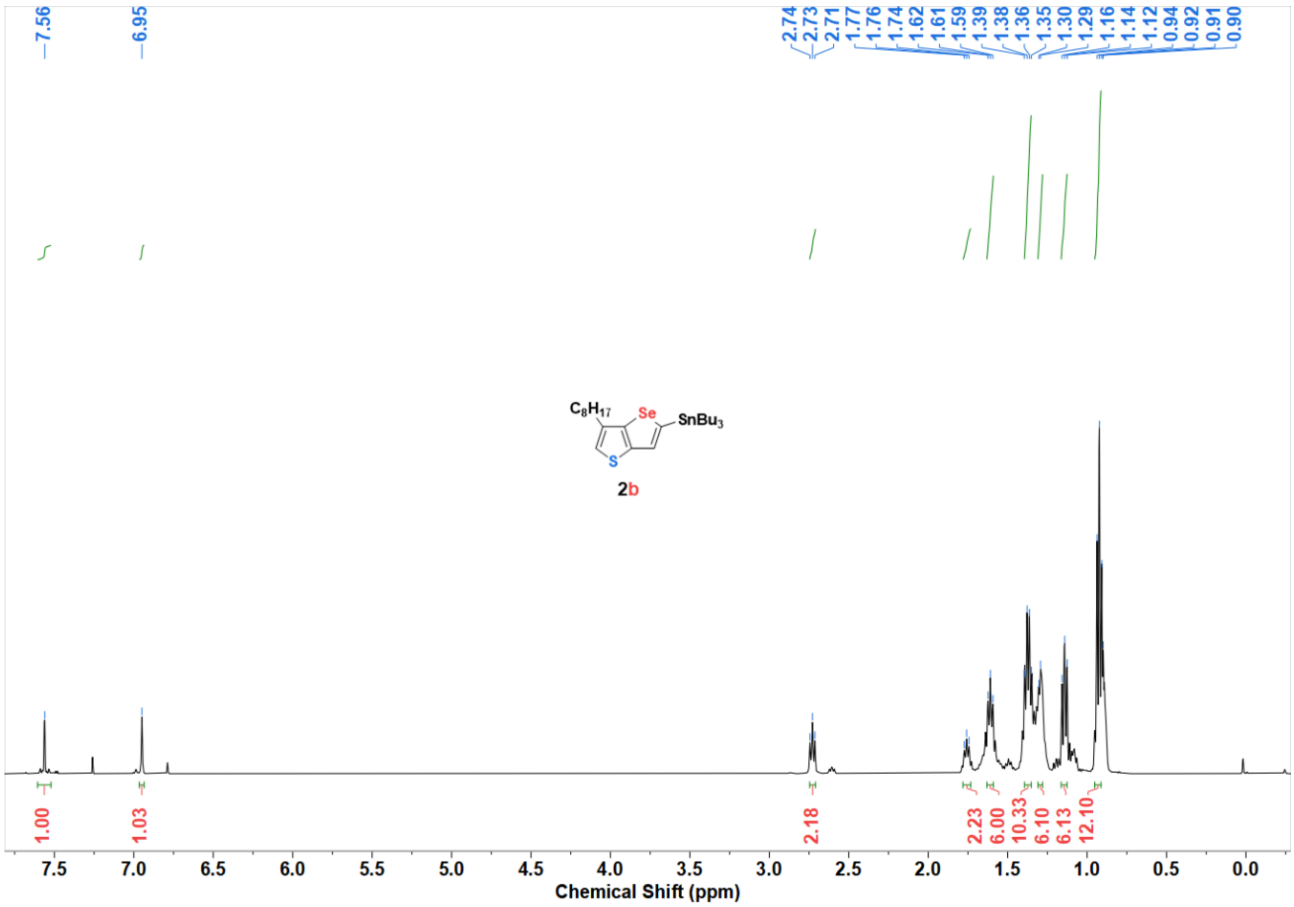

Figure S50. ^{1}H NMR (500 MHz) spectrum of compound 2b.

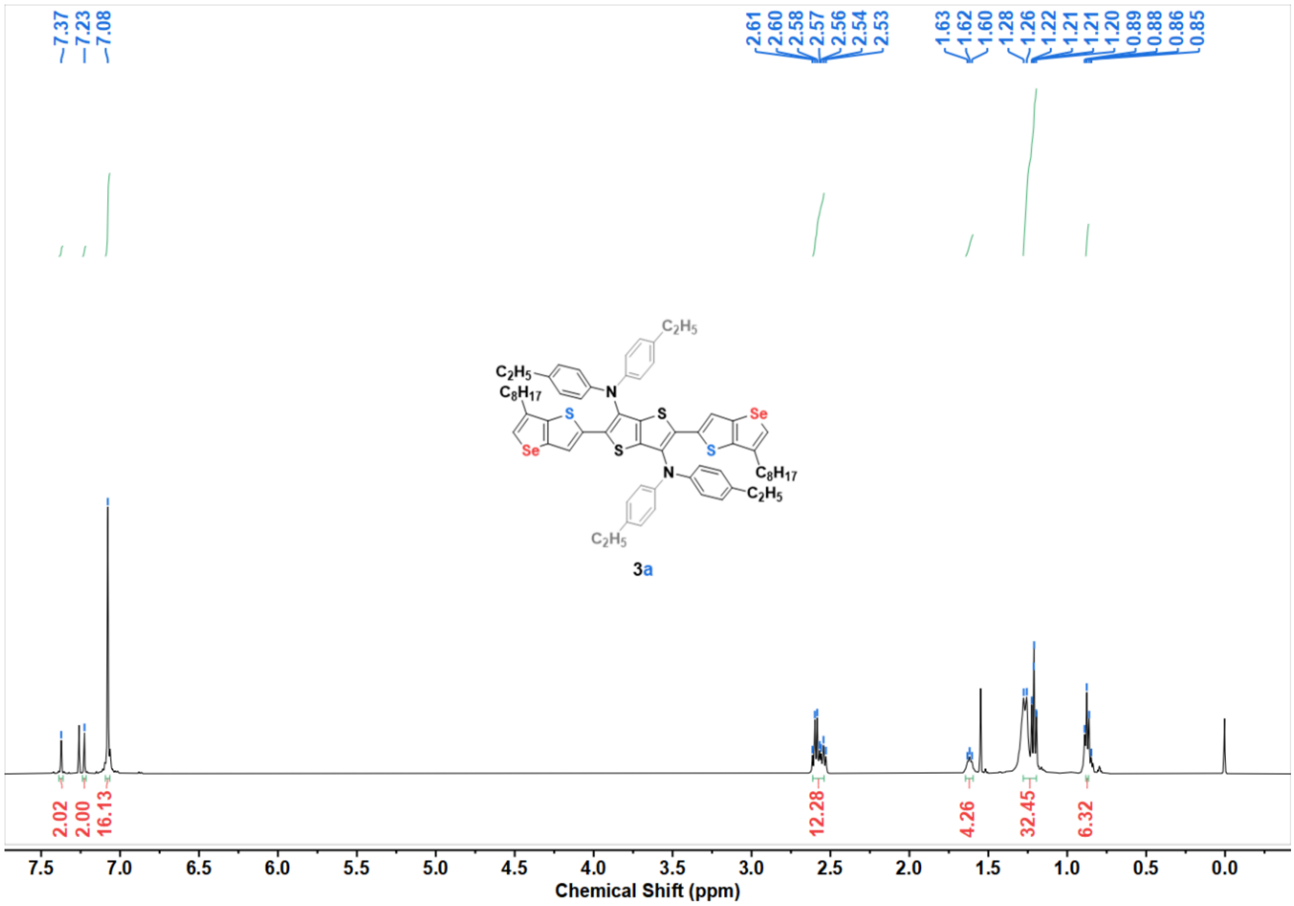

Figure S51. ^{1}H NMR (500 MHz) spectrum of compound 3a.

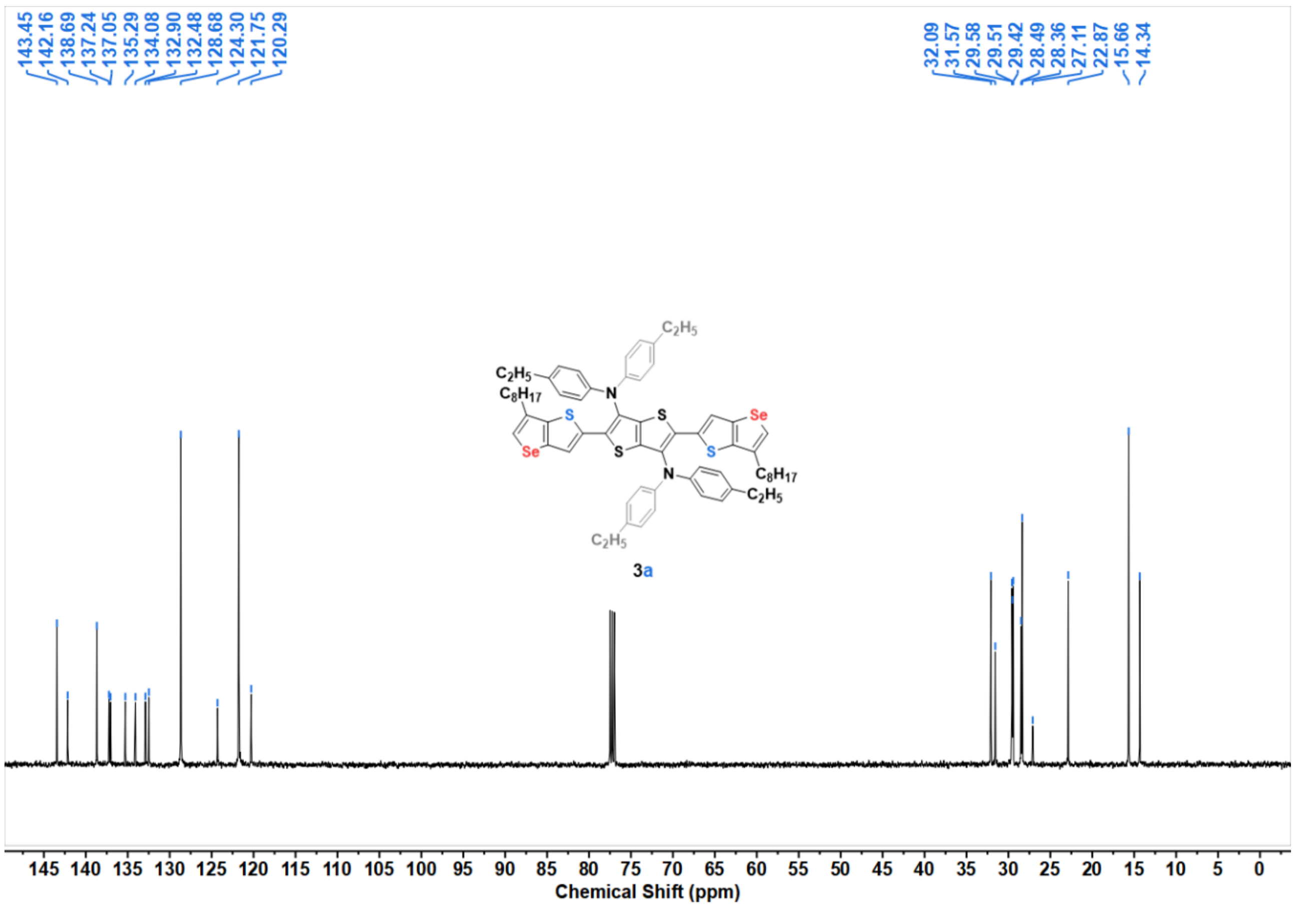

Figure S52. ^{13}C NMR (126 MHz) spectrum of compound 3a.

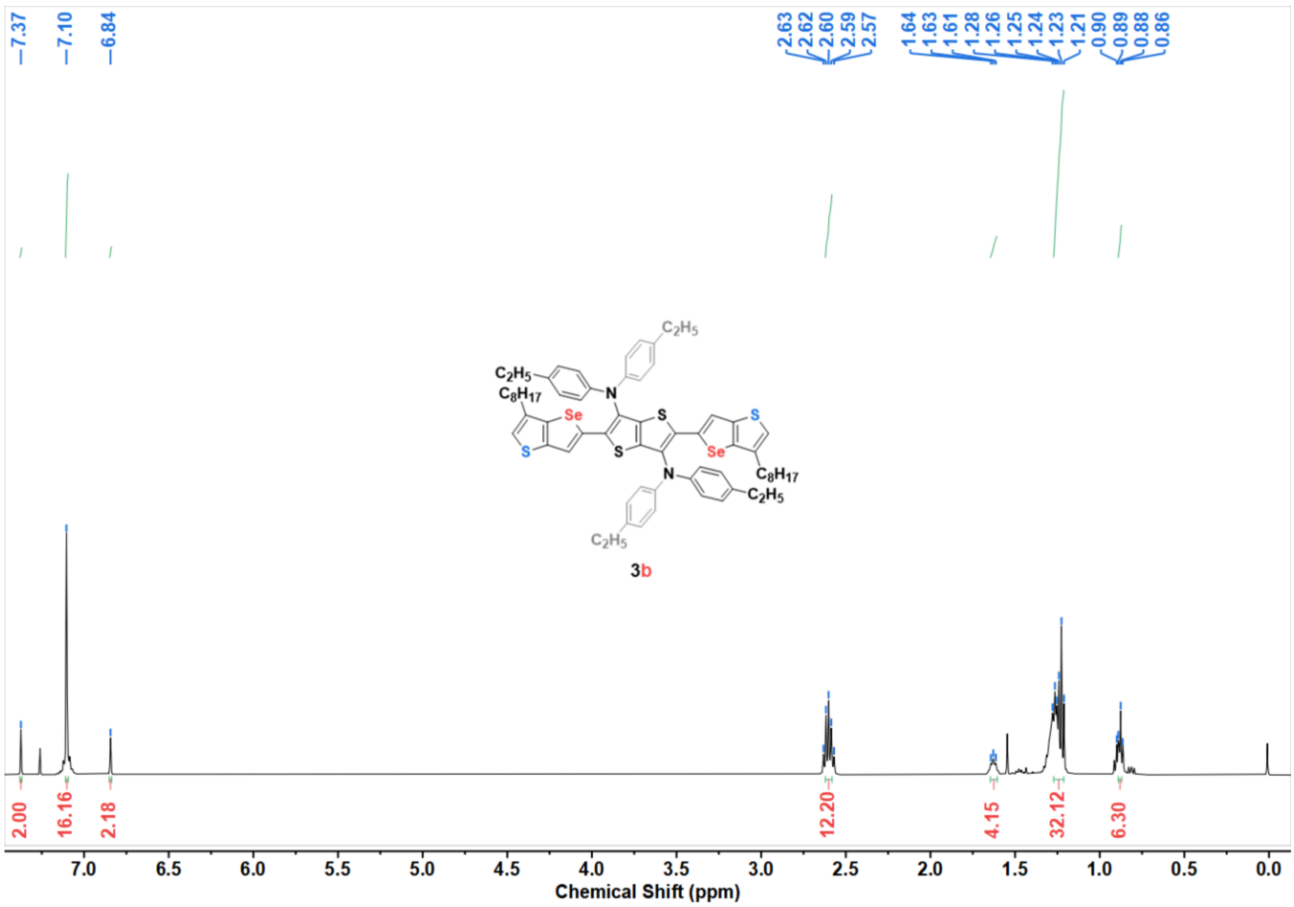

Figure S53. ^{1}H NMR (500 MHz) spectrum of compound 3b.

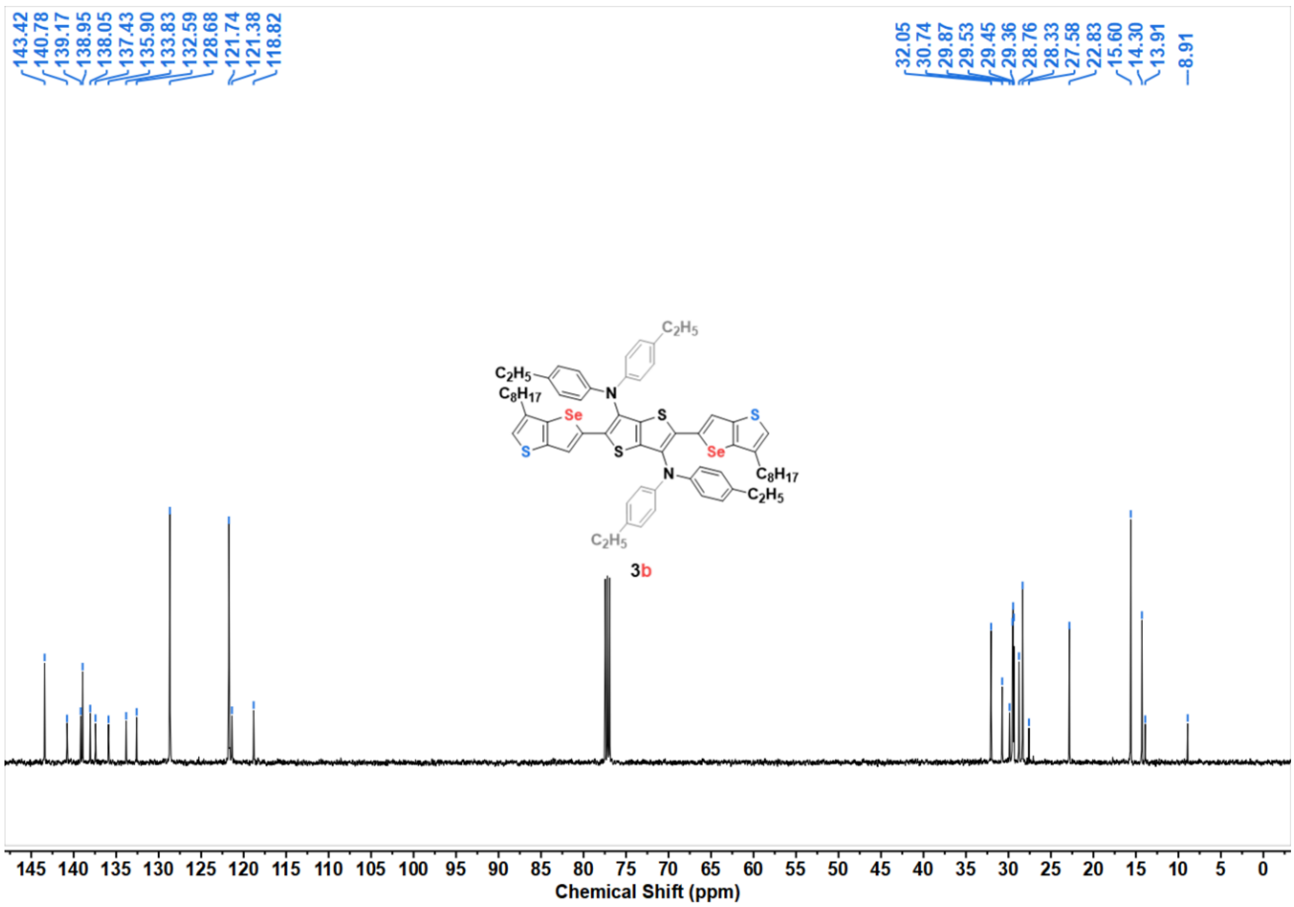

Figure S54. ^{13}C NMR (126 MHz) spectrum of compound 3b.

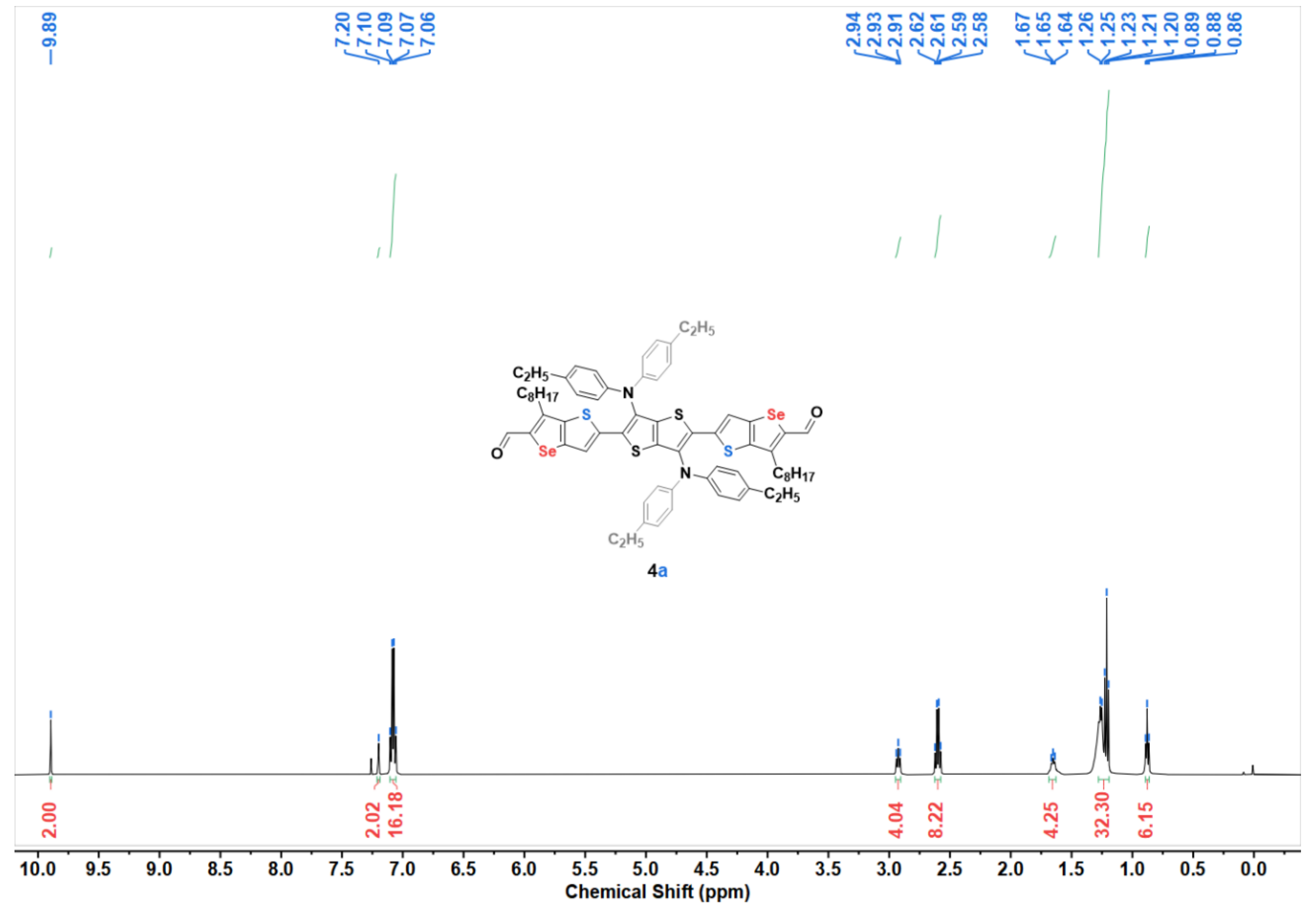

Figure S55. ¹H NMR (500 MHz) spectrum of compound 4a.

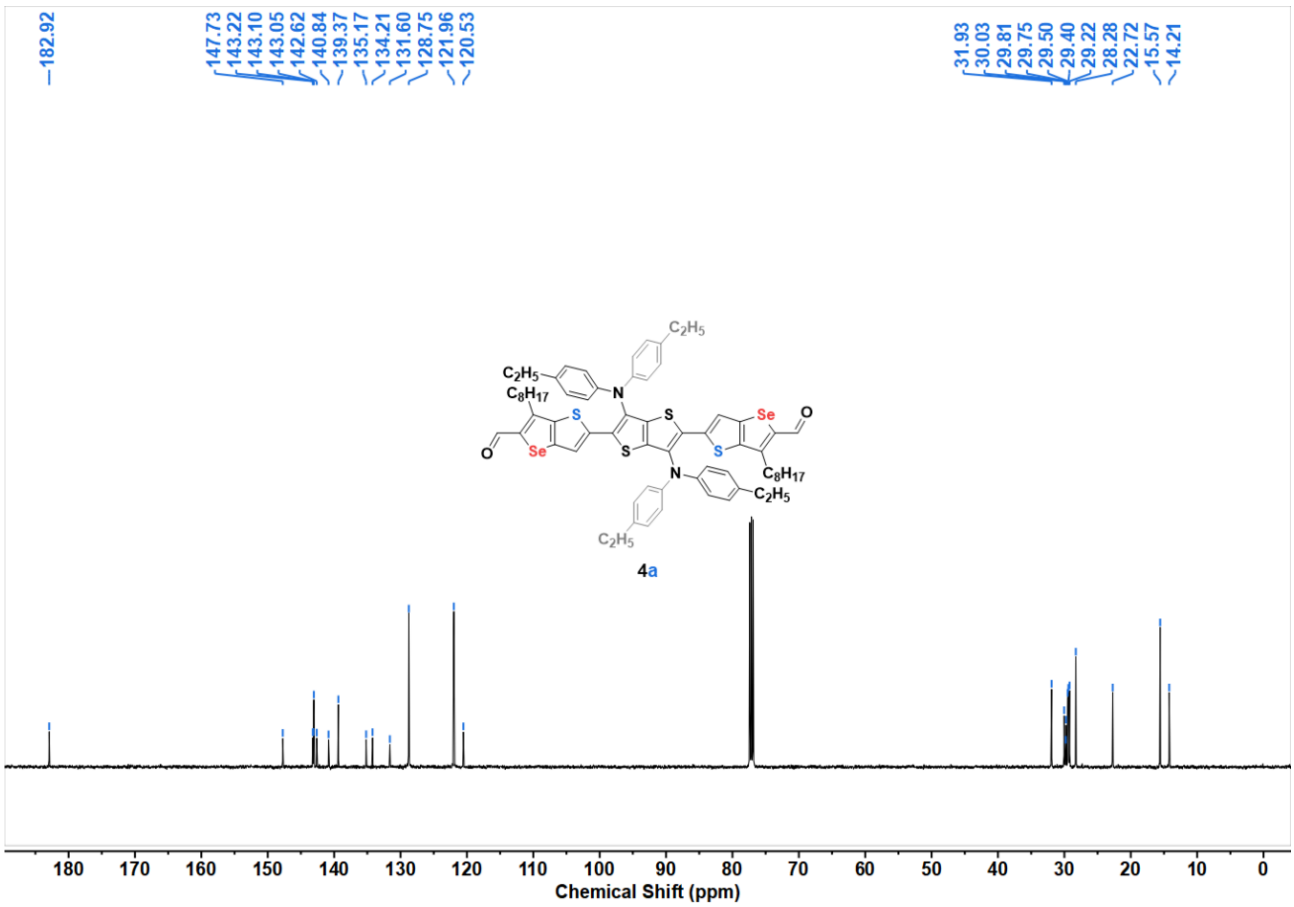

Figure S56. ^{13}C NMR (126 MHz) spectrum of compound 4a.

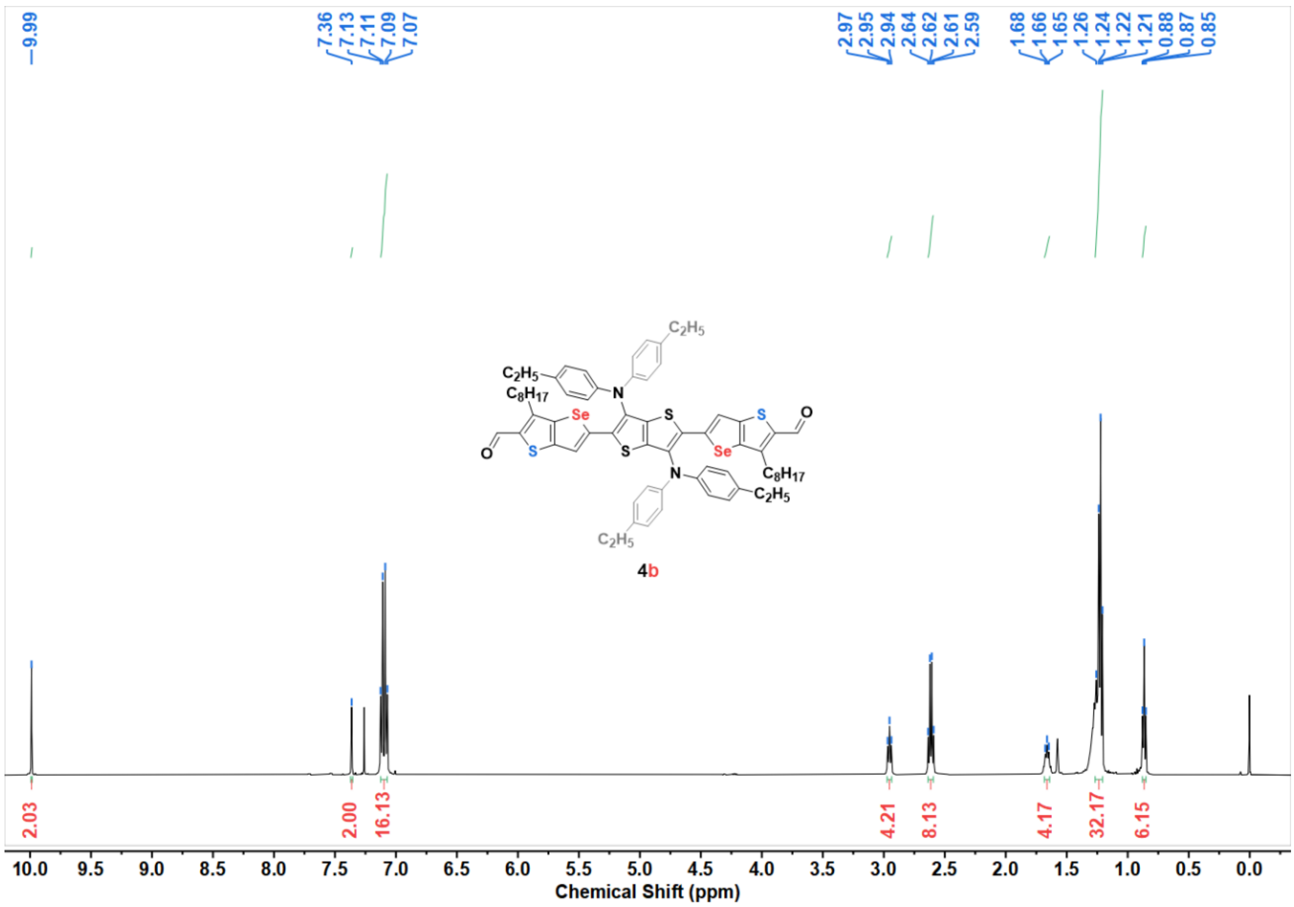

Figure S57. ^{1}H NMR (500 MHz) spectrum of compound 4b.

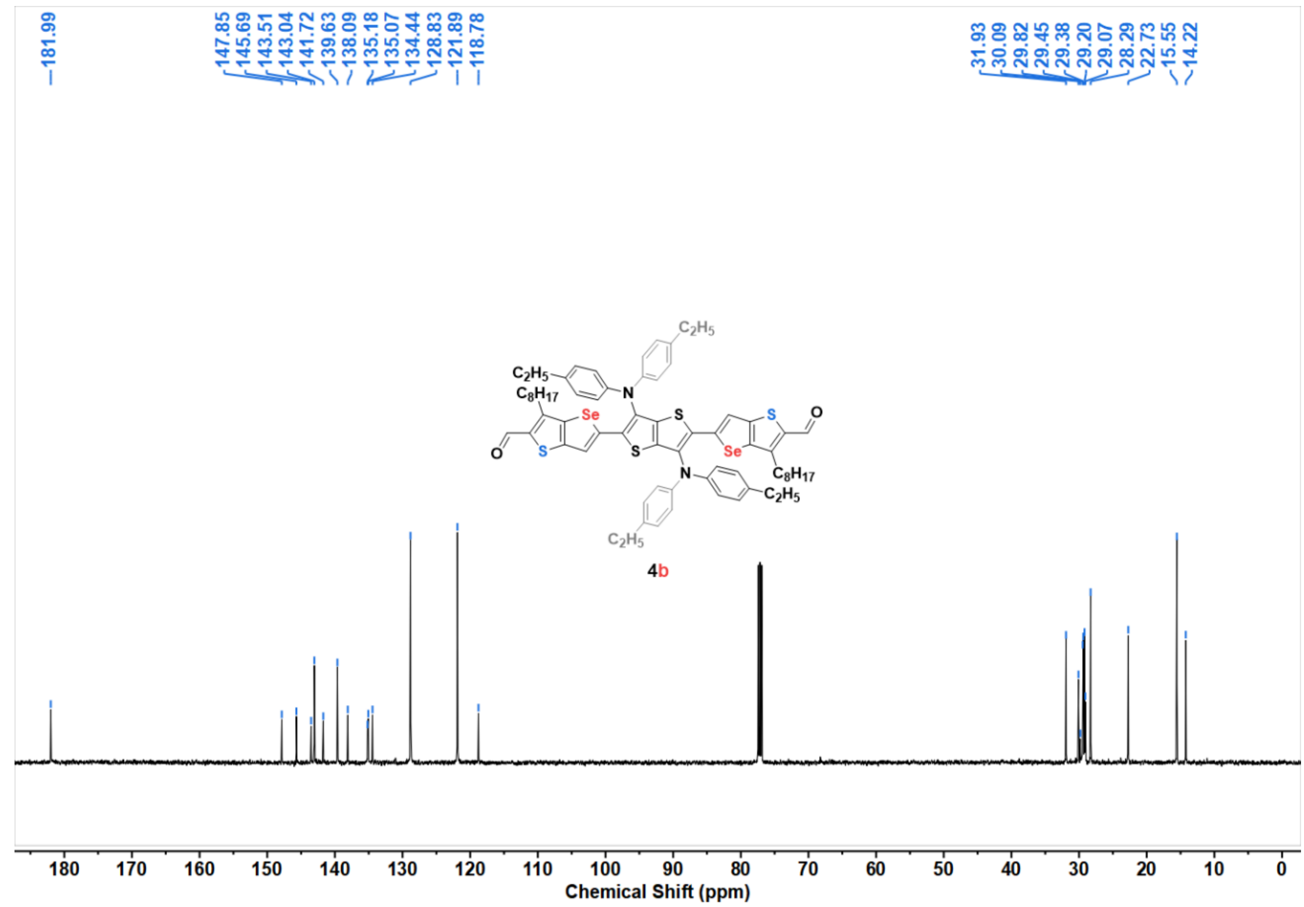

Figure S58. ^{13}C NMR (126 MHz) spectrum of compound 4b.

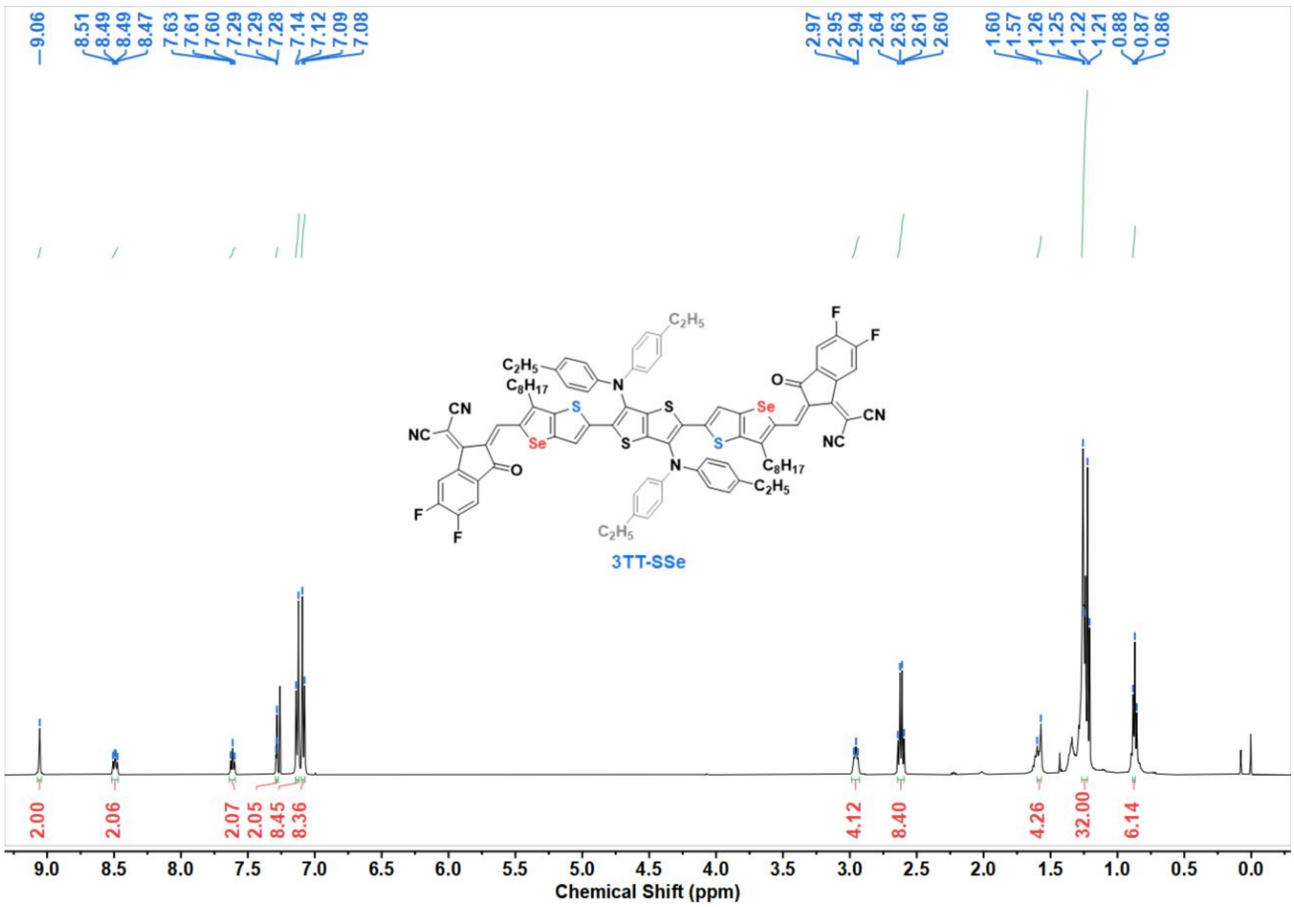

Figure S59. ^{1}H NMR (500 MHz) spectrum of 3TT-SSe.

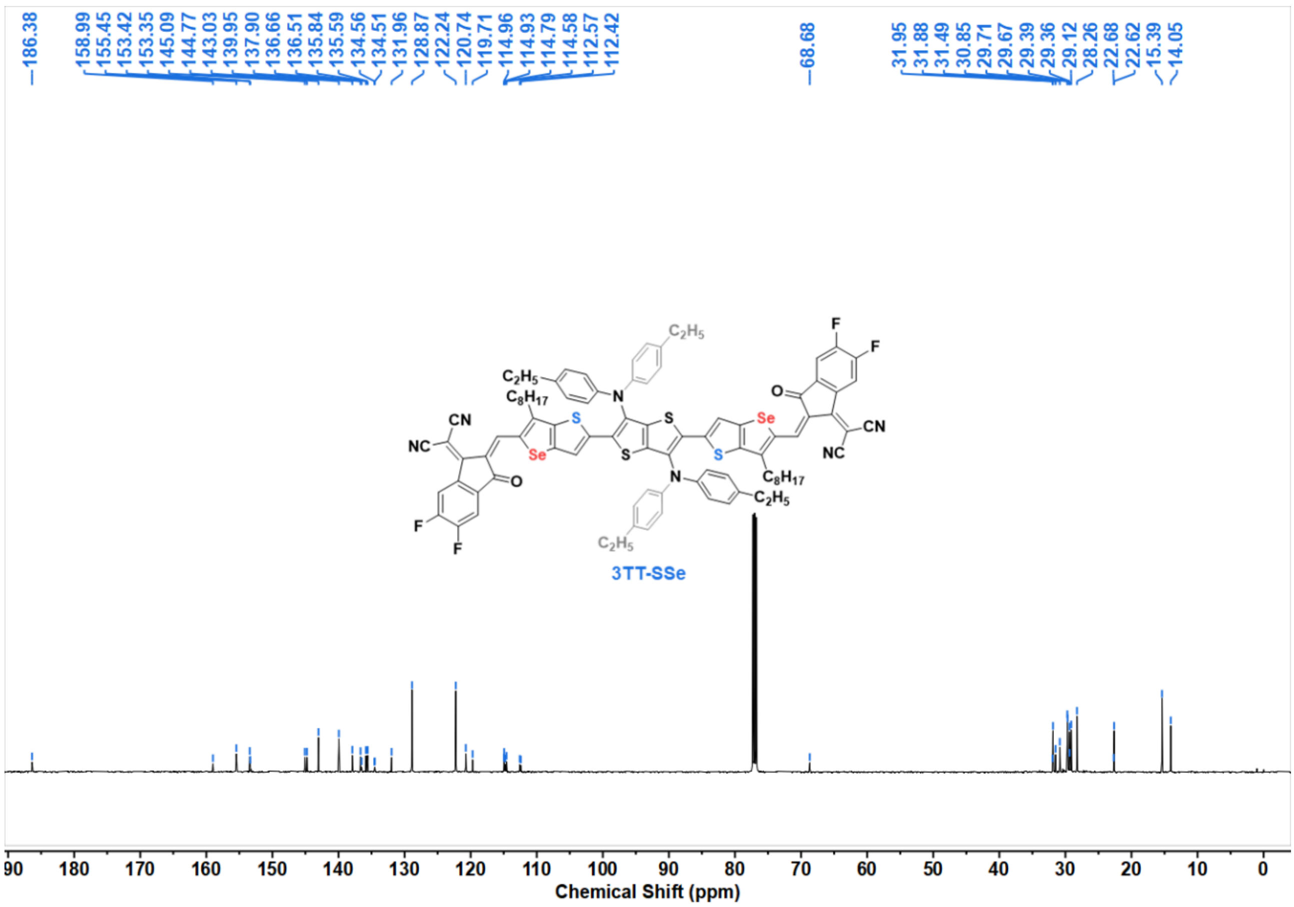

Figure S60. ^{13}C NMR (126 MHz) spectrum of 3TT-SSe.

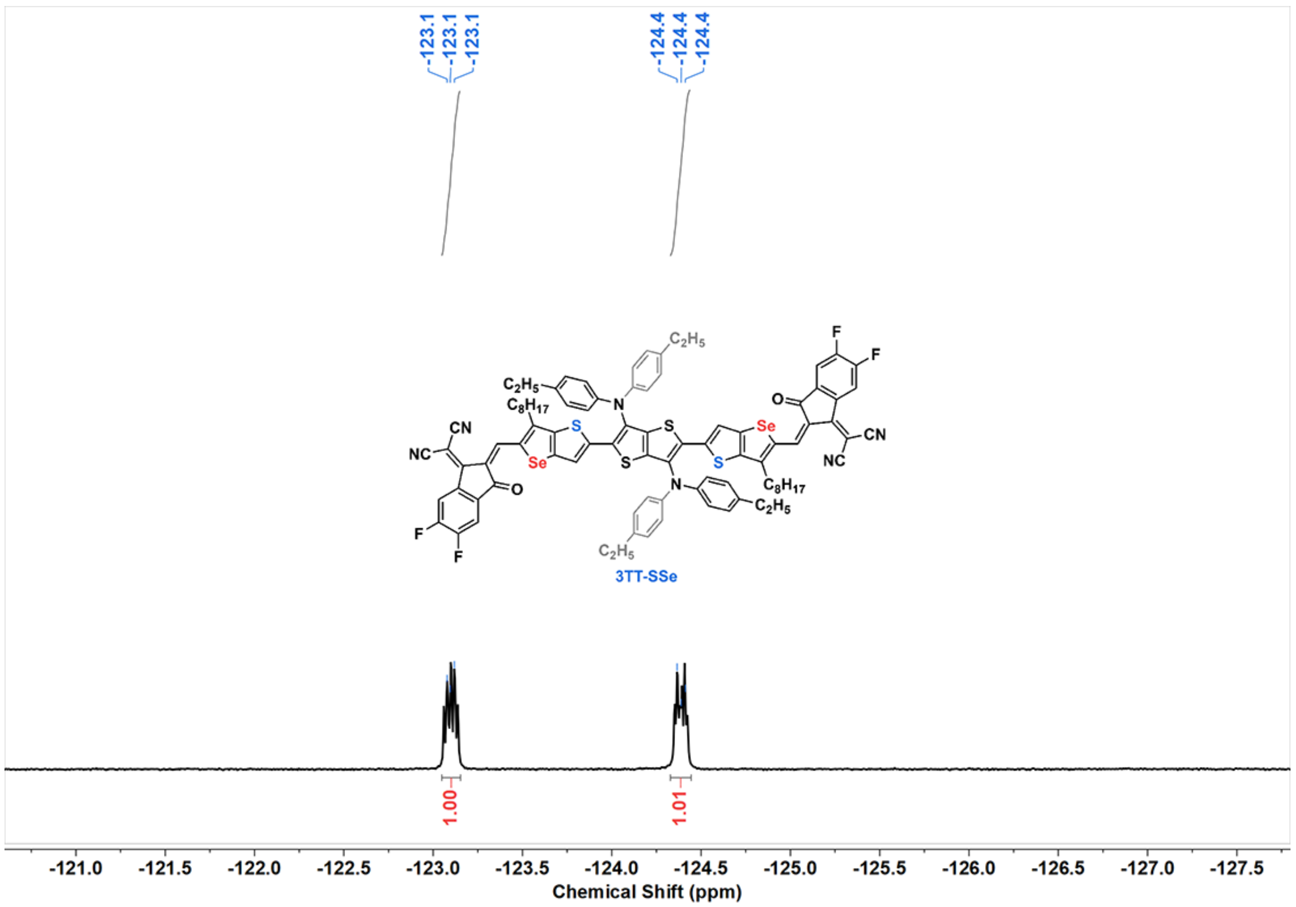

Figure S61. ^{19}F NMR (471 MHz) spectrum of 3TT-SSe.

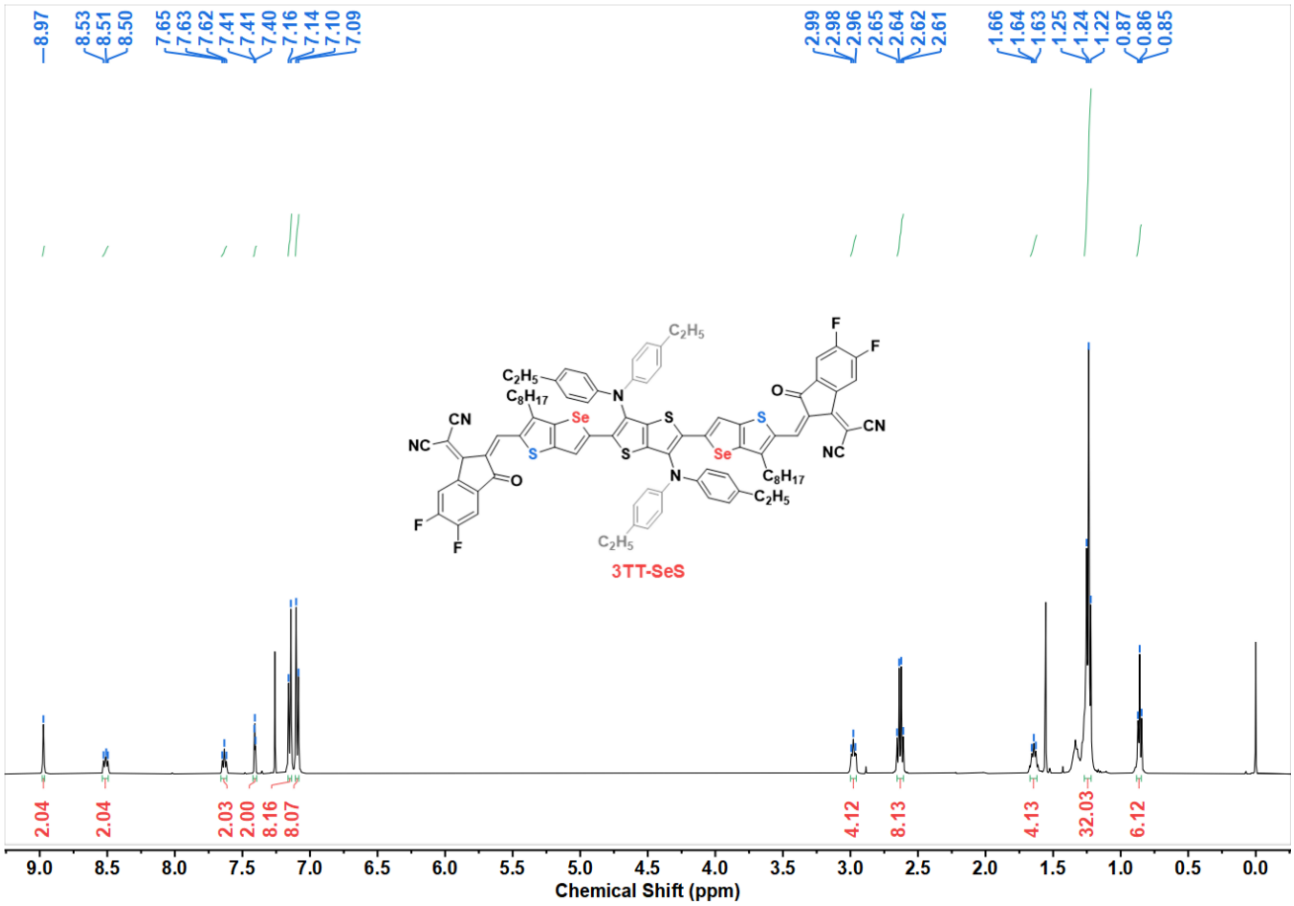

Figure S62. ^{1}H NMR (500 MHz) spectrum of 3TT-SeS.

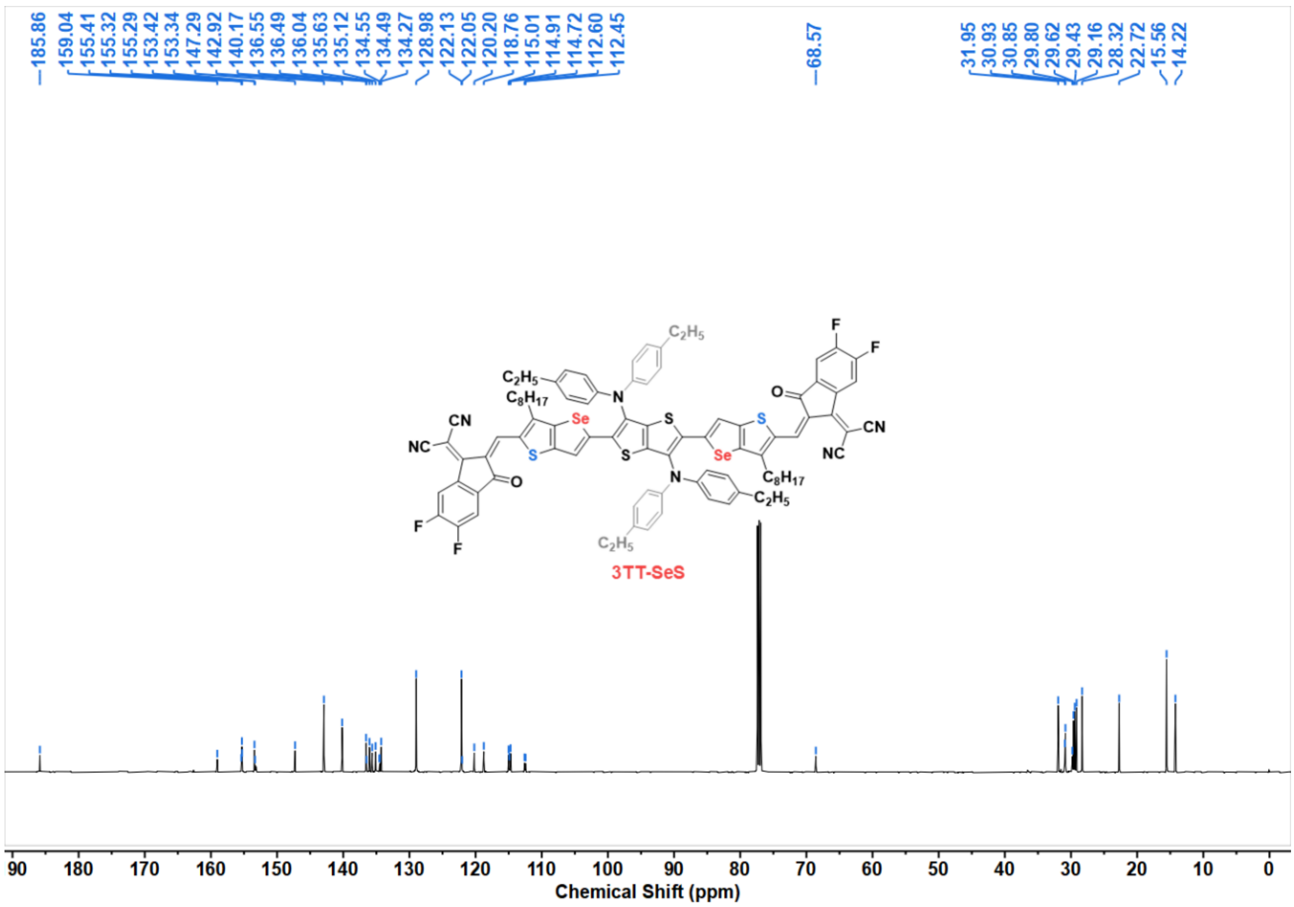

Figure S63. ^{13}C NMR (126 MHz) spectrum of 3TT-SeS.

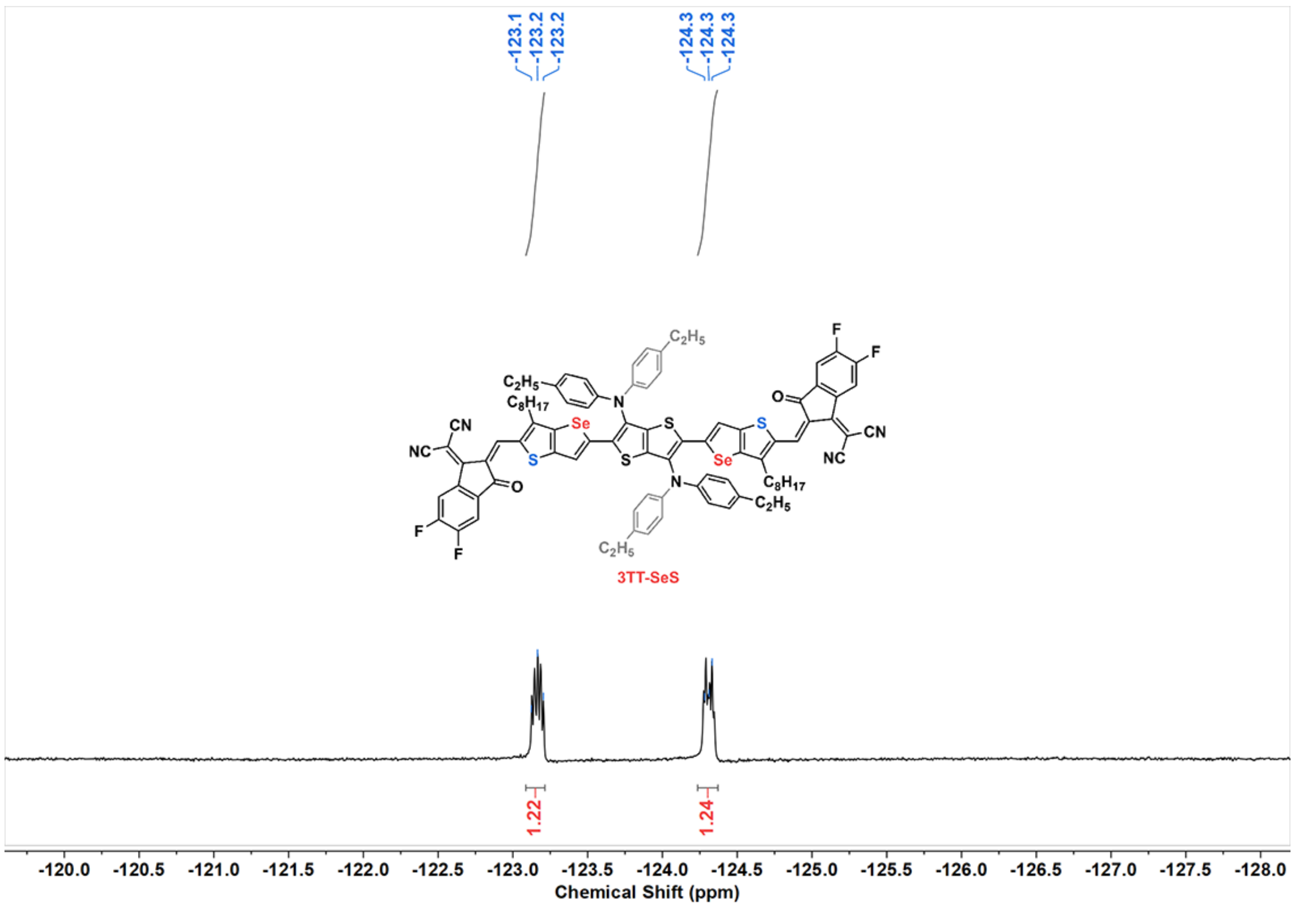

Figure S64. ^{19}F NMR (471 MHz) spectrum of 3TT-SeS.

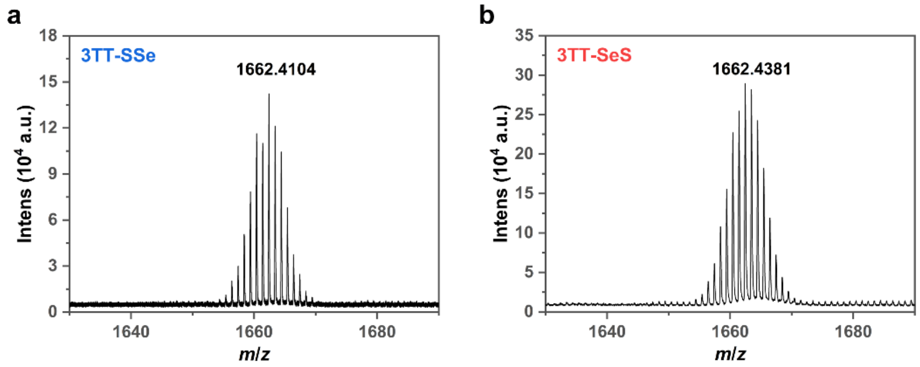

Figure S65. High-resolution mass spectrum of 3TT-SSe (**a**) and 3TT-SeS (**b**).

4 Supplementary Tables

Table S1. The crystal geometric parameters of NFREAs reported in literatures.

NFREAs	CCDC No.	Conformer	θ_1 (°)	d_1 (Å)	$C_{Packing}$ (%)	Refs.
2BTh-2F	2081488	*anti-*	15.07	3.00	55.93	[20]
		anti-	14.87	3.06		
L1	2211870	*anti-*	23.12	3.09	52.67	[35]
		anti-	20.25	3.08		
L2	2211871	*anti-*	23.41	3.10	54.18	[35]
		anti-	21.71	3.09		
DPA-3	2300825	*anti-*	11.86	3.04	58.28	[36]
		anti-	7.96	3.01		
DPA-4	2300827	*anti-*	22.99	2.99	67.20	[36]
		anti-	21.11	3.04		
DPA-5	2300828	*anti-*	30.25	3.05	64.43	[36]
		anti-	28.32	3.10		
3TTS-4F	2289598	*syn-*	148.54	-	66.22	[33]
		syn-	175.63	-		
3TTB-4F	2289599	*anti-*	6.83	3.08	60.42	[33]
		anti-	9.07	3.03		
3TTB-FCl	2330497	*syn-*	179.67	-	64.55	[34]
		anti-	29.25	3.03		
3TTB-ClF	2330498	*anti-*	12.66	2.96	48.93	[34]
		anti-	17.68	3.00		

Table S2. The extracted geometric parameters (dihedral angles (θ), orbital plane angles (α), bond lengths (d)) based DFT optimized molecular structures.

	θ_1 (°)	θ_2 (°)	α_1 (°)	α_2 (°)	d_1 (Å)	d_2 (Å)
3TT-OO	5.01	3.24	146.32	151.31	2.86	2.70
3TT-OS	3.96	0.40	146.23	178.90	2.86	2.70
3TT-OSe	3.25	0.20	146.15	172.92	2.87	2.62
3TT-SO	17.49	0.18	160.10	150.95	3.10	2.70
3TT-SS	18.19	0.43	159.60	178.89	3.12	2.71
3TT-SSe	17.42	0.13	159.99	173.27	3.13	2.63
3TT-SeO	7.23	1.64	160.82	151.04	2.91	2.70
3TT-SeS	7.49	0.38	160.68	179.41	2.93	2.71
3TT-SeSe	6.77	0.10	160.81	173.26	2.94	2.64

Table S3. The calculated *S* value for quantify the strength of INIs[a].

	r_w (Å)	S_1				r_w (Å)	S_2		
		3TT-XO	3TT-XS	3TT-XSe			3TT-OY	3TT-SY	3TT-SeY
X = O (O···S)	3.25	0.188	0.188	0.178	Y = O (O···O)	2.80	0.0097	0.0097	0.0096
X = S (S···N)	3.35	0.069	0.057	0.052	Y = S (S···O)	3.25	0.537	0.513	0.537
X = Se (Se···N)	3.50	0.601	0.547	0.525	Y = Se (Se···O)	3.40	1.385	1.336	1.287

[a] The *S* value is calculated according to Equation: $S = (-\cos\alpha)\cos^2\theta\,[1 - e^{(r_w - d)}]^2$, where r_w is the sum of van der Waals radii of two involved atoms, Δd is defined as $r_w - d$, respectively.

Table S4. The energy of stable state and metastable state, equilibrium constant, and distribution of the conformational isomers.

		G_s[a] (kcal/mol)	G_{ms}[b] (kcal/mol)	ΔG (kcal/mol)	Equilibrium constant (k)[c]	N_s:N_{ms}
σ_1	3TT-OS	–3429798.36	8.442	8.442	0.0332	3.32:100
	3TT-SS	–3835148.61	0.415	0.415	0.8459	84.59:100
	3TT-SeS	–6346696.08	14.245	14.245	0.0032	0.32:100
σ_2	3TT-SO	–3429791.75	3.613	3.613	0.2328	23.28:100
	3TT-SS	–3835148.61	18.858	18.858	0.0005	0.05:100
	3TT-SSe	–6346690.89	34.440	34.440	9×10^{-7}	0.44:100

[a] The zero point of energy with reference to the lowest point of energy.

[b] Relative to stable state energy.

[c] Equilibrium constant of the conformational isomers: $k = N_{\text{stable state}}:N_{\text{metastable state}} = \exp(-\Delta E/RT)$, where R is the gas constant (8.314 J/(mol K), T is the temperature in Kelvin (298.15 K).

Table S5. The MOC evaluation of 3-alkylselenopheno[3,2-*b*]thiophene (3R-SeS) and 3-alkylselenopheno[3,2-*b*]selenophene (3R-SeSe).[a, b]

**3-alkylselenopheno[3,2-*b*]thiophene (1g)**			
Reagent	Quantity	Unit (source) (By Dec. 2024; www.casmart.com.cn)	Cost (¥)
3-bromothiophene	3.54 g	¥235/100 g (*J&K*)	8.11
n-BuLi	9.41 mL	¥443/500 mL (2.4 M, *J&K*)	8.34
selenium power	1.85 g	¥181/25 g (*J&K*)	13.39
Br-CH_2CO_2Et	3.99 g	¥77/25 g (*J&K*)	12.29
N-methylformanilide	3.40 g	¥131/100 g (*J&K*)	4.45
$POCl_3$	3.55 g	¥76.42/100 g (Aladdin)	2.71
K_2CO_3	6.72 g	¥150.1/500 g (*J&K*)	2.02
LiOH	706.53 mg	¥368/100 g (*J&K*)	2.60
NBS	2.48 g	¥53/5 g (*J&K*)	26.29
LDA	13.03 mL	¥258/500 mL (2.0 M, *J&K*)	6.72
n-octylboronic acid	2.07 g	¥59/5 g (*J&K*)	24.43
$Pd(OAc)_2$	39.20 mg	¥298/1 g (*J&K*)	11.68
RuPhos	162.97 mg	¥35.61/1 g (Aladdin)	5.80
K_2CO_3	3.63 g	¥150.1/500 g (*J&K*)	1.09
Ag_2CO_3	144.49 mg	¥183/5 g (*J&K*)	5.29
AcOH	64 µL	¥132/1 L (*J&K*)	0.008
**Total cost (R substituent as *n*-octyl):**		**¥135.22 ($18.53)**	

3-alkylselenopheno[3,2-*b*]selenophene (1g)			
Reagent	Quantity	Unit (source)<br>(By Dec. 2024; www.casmart.com.cn)	Cost (¥)
3-bromoselenophene	19.04 g	¥5060/1 g (*J&K*)	96,342.4
n-BuLi	104.43 mL	¥443/500 mL (2.4 M, *J&K*)	92.5
selenium power	7.90 g	¥181/25 g (*J&K*)	57.20
Br-CH_2CO_2Et	18.22 g	¥77/25 g (*J&K*)	56.12
$SnCl_4$	7.00 g	¥58/25 g (*J&K*)	16.24
nonanoyl chloride	4.62 g	¥69/25 g (*J&K*)	12.75
NaOEt	1.38 g	¥187/100 g (*J&K*)	2.58
Ag_2CO_3	125.08 mg	¥183/5 g (*J&K*)	4.58
AcOH	55 µL	¥132/1 L (*J&K*)	0.007
**Total cost (R substituent as *n*-octyl):**		**¥96,584.377 ($13,232.06)**	

[a] The reactants shown in Figure S6 are taken into account, excluding organic solvents, inorganic substances (acids, bases and salt), solvents for post-treatment, water, electricity, rent and labor costs.

[b] The prices were sourced from reputable suppliers, including *J&K* and Aladdin.

Table S6. Relevant parameters involved in the calculation of conformation distribution.

NFREAs	Conformers	θ_1 (°)	θ_2 (°)	d_1 (Å)	d_2 (Å)	Relative G (kJ/mol)	Boltzmann weight (%)	$S_{conf.}$ (J/mol/K)
3TT-SSe	Conf. 1	0.29	17.27	2.63	3.13	0.000	33.633	9.849
	Conf. 2	0.27	17.99	2.63	3.13	0.038	33.126	
	Conf. 3	0.28	18.03	2.63	3.13	0.175	31.340	
	Conf. 4	0.45	19.87	2.63	3.15	8.790	0.970	
	Conf. 5	0.49	20.18	2.63	3.15	8.892	0.931	
3TT-SeS	Conf. 1	0.06	13.31	2.71	2.97	0.000	99.750	0.162
	Conf. 2	0.53	15.83	2.71	2.98	16.509	0.128	
	Conf. 3	0.58	16.27	2.71	2.99	16.763	0.115	
	Conf. 4	0.92	18.28	2.71	3.00	25.538	0.003	
	Conf. 5	0.95	18.67	2.71	3.00	25.763	0.003	

Table S7. Crystallographic data for 3TT-SSe and 3TT-SeS.

	**3TT-SSe**	**3TT-SeS**
CCDC Deposition Number	2416621	2416620
Empirical Formula	$C_{92}H_{78}F_4N_6O_2S_4Se_2$	$C_{92}H_{78}F_4N_6O_2S_4Se_2$
Formula weight	1661.76	1661.76
Temperature/K	193.00	223.00
Crystal system	triclinic	monoclinic
Space group	*P*-1	$P2_1/c$
a/Å	13.9994(4)	14.7729(11)
b/Å	18.5767(6)	38.061(2)
c/Å	20.1286(6)	7.8070(5)
α/°	102.782(2)	90
β/°	101.739(2)	97.277(3)
γ/°	104.597(2)	90
Volume/Å^3	4750.2(3)	4354.3(5)
Z	2	8
ρ_{calc}/(g/cm^3)	1.162	1.267
μ/mm^{-1}	2.225	1.003
F(000)	1712.0	1712.0
Crystal size/mm^3	0.12 × 0.1 × 0.09	0.12 × 0.1 × 0.08
Radiation	Cu-K$_\alpha$ (λ = 1.54178)	Mo-K$_\alpha$ (λ = 0.71073)
2θ range for data collection/°	5.132 to 136.486	4.28 to 51.402
Index ranges	$-16 \le h \le 16$, $-22 \le k \le 22$, $-22 \le l \le 24$	$-18 \le h \le 18$, $-46 \le k \le 46$, $-9 \le l \le 9$
Reflections collected	61455	40484
Independent reflections	17353 [R_{int} = 0.0638, R_{sigma} = 0.0539]	8225 [R_{int} = 0.0469, R_{sigma} = 0.0375]
Data/restraints/parameters	17353/740/971	8225/63/528
Goodness-of-fit on F^2	1.176	1.038
Final *R* indexes [I >=2σ (I)]	R_1 = 0.1008, wR_2 = 0.2963	R_1 = 0.0566, wR_2 = 0.1760
Final *R* indexes [all data]	R_1 = 0.1286, wR_2 = 0.3305	R_1 = 0.0730, wR_2 = 0.1907
Largest diff. peak/hole/e Å^{-3}	0.64/-1.08	0.74/-0.70

Table S8. Fractional atomic coordinates (×10^4) and equivalent isotropic displacement parameters (Å^2×10^3) for 3TT-SSe and 3TT-SeS.

3TT-SSe				3TT-SeS			
Atom	*x*	*y*	*z*	Atom	*x*	*y*	*z*
Se1	-7615.1(5)	6984.8(4)	6334.4(3)	Se1	2913.0(2)	5960.2(2)	2504.5(5)
S1	-5135.6(14)	9753.9(9)	8921.1(7)	S1	4278.2(6)	6941.3(2)	3779.4(13)
S2	-7025.8(16)	7380.5(10)	8587.8(9)	S2	4516.3(6)	4492.6(2)	4484.8(11)
F1	-10077(5)	2926(4)	2422(3)	F1	5262(2)	8998.4(7)	5239(4)
F2	-9447(5)	4351(5)	2281(3)	F2	3790(2)	9267.6(6)	3430(4)
O1	-8358(4)	6093(4)	4967(3)	O1	4676(2)	7628.1(8)	4248(5)
N1	-9917(6)	2080(5)	4553(6)	N1	1077(4)	8680.3(12)	431(7)
N2	-9000(11)	3595(6)	6569(7)	N2	934(3)	7590.7(12)	-462(7)
N3	-6654(6)	8546(4)	10001(3)	N3	3100.2(18)	5176.9(7)	3085(4)
C4	-9030(4)	4914(3)	3511(3)	C1	3870(3)	8915.6(10)	3489(6)
C5	-8960(3)	4809(3)	4180(3)	C2	4638(3)	8776.2(11)	4418(6)
C6	-9265(4)	4064(4)	4250(3)	C3	4776(3)	8418.5(11)	4530(6)
C1	-9639(4)	3424(3)	3652(3)	C4	4100(3)	8210.0(10)	3671(5)
C2	-9709(4)	3529(4)	2984(3)	C5	3322(3)	8345.5(10)	2727(5)
C3	-9404(4)	4274(4)	2913(2)	C6	3205(3)	8712.9(10)	2644(5)
C7	-9116(5)	4117(5)	4994(5)	C7	4078(3)	7824.4(10)	3575(5)
C8	-8697(5)	4973(5)	5386(5)	C8	3228(3)	7726.7(10)	2529(5)
C9	-8613(5)	5399(5)	4869(4)	C9	2737(3)	8053.2(10)	1996(5)
C10	-9272(6)	3522(6)	5289(6)	C10	1909(3)	8091.1(10)	1023(5)
C11	-9657(6)	2711(5)	4851(6)	C11	1470(3)	8421.8(12)	698(6)
C12	-9121(9)	3575(6)	5994(8)	C12	1371(3)	7808.4(12)	240(6)
C13	-8483(5)	5260(4)	6102(4)	C13	2900(3)	7393.1(10)	2148(5)
C14	-8050(5)	6036(4)	6576(4)	C14	3240(3)	7050.3(9)	2553(5)
C15	-7895(5)	6159(4)	7295(4)	C15	2710(2)	6750.4(9)	2026(5)
C16	-8148(7)	5544(5)	7661(5)	C16	1791(3)	6771.5(12)	985(7)
C17	-7232(8)	5311(6)	7970(6)	C17	1207(4)	6463(2)	783(10)
C18	-7483(13)	4737(9)	8381(9)	C18	253(4)	6555(3)	-139(12)
C19	-6810(13)	4225(10)	8410(12)	C19	-446(7)	6665(4)	953(16)

C20	-7180(17)	3549(9)	8674(10)	C20	-1357(7)	6799(4)	205(18)
C21	-8138(14)	2938(10)	8186(13)	C21	-1817(12)	6969(9)	1680(30)
C22	-7949(16)	2308(15)	7675(14)	C21A	-1675(9)	6666(7)	-1610(30)
C23	-8940(20)	1744(15)	7150(14)	C22	-2794(11)	7049(8)	1130(30)
C24	-7405(5)	6938(4)	7680(4)	C22A	-2666(12)	6755(5)	-2190(30)
C25	-7180(5)	7467(4)	7314(3)	C23	-3305(11)	7225(6)	2400(30)
C26	-6677(5)	8251(4)	7749(3)	C23A	-3198(8)	6453(5)	-3040(20)
C27	-6530(5)	8285(4)	8450(3)	C24	3175(2)	6443.5(9)	2632(4)
C28	-5979(5)	8958(4)	9052(3)	C25	4017(2)	6501.4(9)	3588(5)
C29	-5988(5)	9070(4)	9751(3)	C26	4506(2)	6202.5(9)	4235(5)
C30	-5266(5)	9774(4)	10183(3)	C27	4066(2)	5892.5(9)	3827(4)
C31	-7702(8)	8430(7)	9789(5)	C28	4404(2)	5547.5(9)	4302(4)
C32	-8086(8)	8948(8)	9577(5)	C29	3999(2)	5224.4(8)	3930(4)
C33	-9133(10)	8812(10)	9371(6)	C30	4585(2)	4944.9(8)	4562(4)
C34	-9845(10)	8098(12)	9353(8)	C31	2369(2)	5237.6(8)	4094(4)
C35	-9432(11)	7632(10)	9579(7)	C32	2514(2)	5242.4(10)	5861(5)
C36	-8400(9)	7725(8)	9774(5)	C33	1794(3)	5324.0(13)	6794(6)
C37	-10979(10)	7972(11)	9137(9)	C34	932(3)	5396.5(12)	5964(6)
C38	-11440(17)	7117(12)	8751(13)	C35	796(3)	5384.7(12)	4211(6)
C39	-6253(8)	8355(5)	10609(5)	C36	1496(2)	5310.5(11)	3257(5)
C40	-6677(8)	8403(5)	11183(5)	C37	136(3)	5500.9(16)	6928(7)
C41	-6268(10)	8190(6)	11767(6)	C38	283(4)	5490(3)	8746(8)
C42	-5391(10)	7933(7)	11803(6)	C39	3006(2)	4922.9(9)	1732(4)
C43	-4984(10)	7928(6)	11272(6)	C40	2363(3)	4658.4(11)	1664(5)
C44	-5413(9)	8110(6)	10640(6)	C41	2301(3)	4411.9(11)	335(6)
C45	-4897(11)	7808(7)	12494(6)	C42	2887(3)	4421.4(11)	-927(5)
C46	-5185(16)	6959(7)	12368(10)	C43	3533(3)	4681.4(11)	-818(5)
Se2	-6149.3(5)	5634.4(4)	4281.3(4)	C44	3596(2)	4933.6(9)	484(5)
S3	-4925.0(12)	7802.6(10)	5935.6(7)	C45	2776(3)	4156.9(15)	-2380(6)
S4	-4982.6(12)	11001.1(9)	5714.1(7)	C46	1930(5)	4227(2)	-3654(8)
F3	-8740(6)	541(3)	3622(5)				
F4	-8808(5)	1058(4)	2505(5)				

O2	-6954(4)	4083(3)	3702(3)
N4	-7113(9)	1887(6)	6092(6)
N5	-5883(10)	4338(7)	6905(7)
N6	-4873(3)	9427(3)	6335(2)
C51	-7447(4)	2859(3)	3905(4)
C52	-7410(4)	2567(4)	4485(4)
C47	-7834(5)	1776(4)	4384(4)
C48	-8294(5)	1277(3)	3703(5)
C49	-8331(5)	1569(4)	3122(4)
C50	-7907(5)	2360(4)	3223(4)
C53	-6914(6)	3248(5)	5109(6)
C54	-6711(8)	3218(7)	5791(7)
C55	-6965(9)	2446(6)	5925(7)
C56	-6315(11)	3812(9)	6382(8)
C57	-6681(6)	3955(5)	4890(5)
C58	-7002(6)	3706(5)	4106(6)
C59	-6241(5)	4697(5)	5306(5)
C60	-5967(5)	5456(4)	5180(4)
C61	-5544(5)	6144(4)	5742(4)
C62	-5328(6)	6130(5)	6507(4)
C63	-4817(6)	6899(5)	7046(4)
C64	-4586(7)	6843(6)	7797(4)
C65	-4141(8)	7591(7)	8320(5)
C66	-3845(9)	7541(7)	9063(5)
C67	-3345(10)	8325(8)	9595(6)
C68	-2891(11)	8259(8)	10354(6)
C69	-2545(14)	9056(10)	10907(7)
C70	-5357(5)	6811(4)	5507(3)
C71	-5607(5)	6672(3)	4777(3)
C72	-5444(4)	7368(3)	4567(3)
C73	-5097(4)	8014(3)	5134(3)
C74	-4957(4)	8807(3)	5097(3)

C75	-4899(4)	9455(3)	5638(3)
C76	-4961(4)	10081(3)	5359(3)
C77	-3900(4)	9741(4)	6858(3)
C78	-3797(5)	9489(5)	7475(3)
C79	-2888(6)	9788(6)	7990(4)
C80	-2039(6)	10338(6)	7934(4)
C81	-2139(5)	10530(5)	7305(4)
C82	-3077(5)	10240(4)	6772(3)
C83	-1078(7)	10642(8)	8547(5)
C84	-360(14)	11359(11)	8520(10)
C85	-5771(4)	9502(3)	6547(3)
C86	-6726(4)	8980(4)	6140(3)
C87	-7600(5)	9039(4)	6326(4)
C88	-7575(4)	9587(4)	6914(4)
C89	-6623(5)	10106(4)	7315(4)
C90	-5712(5)	10065(4)	7131(3)
C91	-8536(5)	9618(6)	7136(5)
C92	-8736(7)	9136(7)	7622(6)

Table S9. The dihedral angles (θ), orbital plane angles (α), and bond lengths (d) extracted from single-crystal structures, and calculated S values.

Crystals	Confs.	θ_1 (°)	θ_2 (°)	α_1 (°)	α_2 (°)	d_1 (Å)	d_2 (Å)	S_1	S_2
3TT-SSe	Conf. 1	14.93	1.60	160.25	179.28	2.92	2.70	0.254	1.027
	Conf. 2	19.22	0.66	160.79	171.57	3.03	2.71	0.120	0.977
3TT-SeS	-	0.02	0.10	160.53	177.12	3.02	2.69	0.358	0.563

Table 10. Conformational entropy, packing coefficient, and photophysical properties of 3TT-SSe and 3TT-SeS.

NFREAs	$S_{conf.}$ (J mol^{-1} K^{-1})	$C_{packing}$ (%)	$\lambda_{abs.}^{sol./film}$ (nm)	ε_{max}^{film} (10^5 M^{-1} cm^{-1})	$\lambda_{em.}^{sol./film}$ (nm)	$\Delta\nu^{sol./film}$ (cm^{-1})	g-factor
3TT-SSe	9.849	56.1	745/807	1.21	849/908	1644/1378	0.189
3TT-SeS	0.162	64.7	732/781	1.36	830/872	1613/1336	0.152

Table S11. The reorganization energies (λ) of among the ground state (S_0), the lowest singlet excited state (S_1), and the anion state during the photoelectric conversion processes.

	$\lambda_{S0 \to S1}$	$\lambda_{S1 \to S0}$	λ_{EET}	$\lambda_{S0 \to anion}$	$\lambda_{anion \to S0}$	λ_{ET}
3TT-SSe	71.25	59.33	130.58	103.86	95.47	199.33
3TT-SeS	64.39	51.24	115.63	94.28	87.89	182.17

Table S12. Detailed GIWAXS data of the 3TT-SSe and 3TT-SeS neat films.

	IP		OOP			
	location ($Å^{-1}$)	*d*-spacing (Å)	location ($Å^{-1}$)	*d*-spacing (Å)	CCL (Å)	*g*-factor
3TT-SSe	0.31	20.27	1.68	3.74	16.61	0.189
3TT-SeS	0.34	18.48	1.71	3.67	25.13	0.152

Table S13. Photovoltaic parameters of the D18:3TT-SSe devices under different optimization conditions.

D18 (mg mL^{-1})	3TT-SSe (mg mL^{-1})	DIB (mg mL^{-1})	V_{oc} (V)	J_{sc} (mA cm^{-2})	FF (%)	PCE (%)
	7.0		0.898	22.91	72.47	14.91
	7.7		0.898	23.39	73.00	15.33
7	8.4	-	0.899	23.88	73.41	15.76
	9.1		0.900	23.57	72.85	15.45
	9.8		0.901	23.04	72.51	15.05
		2.0	0.898	23.79	73.64	15.73
		2.5	0.900	24.05	73.97	16.01
7	8.4	3.0	0.900	24.22	74.20	16.17
		3.5	0.902	24.11	74.00	16.09
		4.0	0.903	23.87	73.85	15.92

Table S14. Photovoltaic parameters of the D18:3TT-SeS devices under different device optimization conditions.

D18 ($mg\ mL^{-1}$)	3TT-SeS ($mg\ mL^{-1}$)	DIB ($mg\ mL^{-1}$)	V_{oc} (V)	J_{sc} ($mA\ cm^{-2}$)	FF (%)	PCE (%)
	7.0		0.923	23.36	75.00	16.17
	7.7		0.924	23.72	75.89	16.63
7	8.4	-	0.924	24.23	76.51	17.13
	9.1		0.925	24.05	76.11	16.93
	9.8		0.925	23.87	75.62	16.70
		2.0	0.922	24.53	76.90	17.39
		2.5	0.923	25.01	77.65	17.92
7	8.4	3.0	0.925	25.47	78.21	18.44
		3.5	0.925	24.88	77.37	17.80
		4.0	0.926	24.14	77.02	17.22

Table S15. Summary of MOC evaluation (C_g) for representative donors and acceptors.

	D18	PM6	D18-Cl	L8-BO	Z8
C_g ($ g^{-1})	276.36	221.34	183.72	518.10	3743.83
	L8-BO-C_4	L8-BO-C_4-Br	BTP-C3F	3TT-SeS	3TTB-ClF
C_g ($ g^{-1})	3263.32	2835.51	1352.50	61.69	60.84

Table S16. Summary of PCE, C_g, and CPK for representative organic photovoltaic systems.

Active layers	PCE (%)	D:A (w:w)	C_g of acceptors ($ g^{-1})	C_g of blend ($ g^{-1})	Area of per cell (cm^{-2})	Cost of per cell (10^{-7} $)[a]	Power generation of per cell (10^{-7} kW)	CPK ($ kW^{-1})[b]	Refs.
D18:L8-BO:Z8	20.20	1:0.9:0.3	1324.53	848.09	0.03159	26.79	6.38	4.20	[29]
PM6:L8-BO-C_4:L8-BO-C_4-Br	20.42	1:1:0.2	3192.02	1841.71	0.03152	58.05	6.44	9.01	[31]
D18-Cl:BTP-C3F	20.80	1:1	1352.50	768.11	0.04	30.72	8.32	3.69	[32]
D18:3TT-SeS:3TTB-ClF	19.26	1:1.2:0.3	60.84	147.46	0.0309	4.56	5.95	0.77	This work

[a] The thickness and density of the active layer are assumed to be 100 nm and 1 g cm^{-3} for calculation

[b] The solar-cell performance test used an Air Mass 1.5 Global (AM 1.5 G) solar simulator with an irradiation intensity of 100 mW cm^{-2}.

Table S17. The MOC evaluation of D18.

Reagent	Quantity	Unit (source) (By Dec. 2024; www.casmart.com.cn)	Cost (¥)
3-bromo-2-(2-ethylhexyl)thiophene	4.14 g	¥1600/5 g (Alfachem)	1324.8
benzo[1,2-*b*:4,5-*b*']dithiophene-4,8-dione	1.10 g	¥246/5 g (*J&K*)	54.12
LDA	7.52 mL	¥88/100 mL (2.0 M, *J&K*)	6.62
$SnCl_2$	6.65 g	¥81/25 g (*J&K*)	21.55
LDA	2.55 mL	¥88/100 mL (2.0 M, *J&K*)	2.24
$Si(CH_3)_3Cl$	1.14 g	¥149/25 g (*J&K*)	6.79
n-BuLi	2.55 mL	¥443/500 mL (2.4 M, *J&K*)	2.26
$PhSO_2NF$	2.32 g	¥639/25 g (*J&K*)	59.30
CF_3COOH	10 mL	¥451/250 mL (*J&K*)	18.04
LDA	1.78 mL	¥88/100 mL (2.0 M, *J&K*)	1.57
$Sn(CH_3)_3Cl$	1.98 g	¥369/5 g (*J&K*)	146.12
**Cost of BDT-F-Sn (1g)**		**¥732.61**	
(4-(2-butyloctyl) thiophen-2-yl)trimethylstannane	980.04 mg	¥500/5 g (Derthon)	98.00
5,8-dibromodithieno [3',2':3,4;2",3":5,6]benzo[1,2-*c*][1,2,5] thiadiazole	479.53 mg	¥1400/2 g (Derthon)	335.67
$Pd(PPh_3)_4$	68.18 mg	¥87/1 g (*J&K*)	59.32
NBS	382.90 mg	¥211/25 g (*J&K*)	3.23
**Cost of DTBT-T-2Br (1g)**		**¥621.69 (¥496.22/798.17 mg)**	
DTBT-T-2Br	798.17 mg	¥621.69/1 g (self-made)	496.22
BDT-F-Sn	828.57 mg	¥732.61/1 g (self-made)	607.02
Pd_2dba_3	24.18 mg	¥976.74/10 g (Bidepharm)	2.36
P(*o*-tol)$_3$	8.04 mg	¥1047/25 g (*J&K*)	0.34

**Total cost (D18, 1g)**	**¥2016.74 ($276.36)**

Table S18. The MOC evaluation of PM6.

Reagent	Quantity	Unit (source)<br>(By Dec. 2024; www.casmart.com.cn)	Cost (¥)
1,3-dibromo-5,7-bis(2-ethylhexyl)-4*H*,8*H*-benzo[1,2-*c*:4,5-*c*']dithiophene-4,8-dione	1.38 g	¥600.70/1 g (Derthon)	828.97
trimethyl(thiophen-2-yl)stannane	1.69 g	¥2350/5 g (*J&K*)	794.3
$Pd(PPh_3)_4$	263.47 mg	¥87/1 g (*J&K*)	20.42
NBS	809.9 mg	¥211/25 g (*J&K*)	6.84
**Cost of BDD-2Br (1g)**		**¥1650.53**	
2-(2-ethylhexyl) thiophene	917.93 mg	¥7447/50 g (*J&K*)	136.72
benzo[1,2-*b*:4,5-*b*']dithiophene-4,8-dione	363.04 mg	¥246/5 g (*J&K*)	17.86
n-BuLi	1.72 mL	¥443/500 mL (2.4 M, *J&K*)	1.52
$SnCl_2$	2.19 g	¥81/25 g (*J&K*)	7.10
LDA	1.68 mL	¥88/100 mL (2.0 M, *J&K*)	1.48
$Sn(CH_3)_3Cl$	801.06 mg	¥369/5 g (*J&K*)	59.12
**Cost of BDT-Sn (1g)**		**¥223.8**	
BDT-Sn	985.97 mg	¥223.8/1 g (self-made)	220.66
BDD-2Br	838.28 mg	¥1650.53/1 g (self-made)	1383.62
$Pd(PPh_3)_4$	125.96 mg	¥87/1 g (*J&K*)	10.96
**Total cost (PM6, 1g)**		**¥1615.24 ($221.34)**	

Table S19. The MOC evaluation of D18-Cl.

Reagent	Quantity	Unit (source) (By Dec. 2024; www.casmart.com.cn)	Cost (¥)
3-chloro-2-(2-ethylhexyl)thiophene	907.00 mg	¥750/1 g (*J&K*)	680.25
benzo[1,2-*b*:4,5-*b*']dithiophene-4,8-dione	287.53 mg	¥246/5 g (*J&K*)	14.45
n-BuLi	2.13 mL	¥443/500 mL (2.4 M, *J&K*)	1.89
$SnCl_2$	1.74 g	¥81/25 g (*J&K*)	5.64
LDA	1.67 mL	¥88/100 mL (2.0 M, *J&K*)	1.47
$Sn(CH_3)_3Cl$	729.93 mg	¥369/5 g (*J&K*)	53.87
DTBT-T-2Br	932.93 mg	¥621.69/1 g (self-made)	579.99
Pd_2dba_3	28.30 mg	¥976.74/10 g (Bidepharm)	2.76
P(*o*-tol)$_3$	9.41 mg	¥1047/25 g (*J&K*)	0.39
**Total cost (D18-Cl, 1g)**		**¥1340.71 ($183.72)**	

Table S20. The MOC evaluation of L8-BO.

Reagent	Quantity	Unit (source) (By Dec. 2024; www.casmart.com.cn)	Cost (¥)
3-butylnonan-1-ol	486.90 mg	¥1000/5 g (Derthon)	97.38
PCC	784.60 mg	¥45/25 g (*J&K*)	1.41
3-bromothieno[3,2-*b*]thiophene	398.78 mg	¥842/1 g (TCI)	335.77
n-BuLi	0.83 mL	¥443/500 mL (2.4 M, *J&K*)	0.74
$AlCl_3$	334.02 mg	¥41/100 g (*J&K*)	0.14
$LiAlH_4$	190.15 mg	¥237.91/25 g (Aladdin)	1.81
n-BuLi	0.77 mL	¥443/500 mL (2.4 M, *J&K*)	0.68
Sn(*n*-Bu)$_3$Cl	706.86 mg	¥51.77/25 g (Aladdin)	1.46
**Cost of TT-BO-Sn (1g)**		**¥439.39**	
TT-BO-Sn	3.25 g	¥223.25/1 g (self-made)	1428.02
4,7-dibromo-5,6-dinitrobenzo[*c*][1,2,5]thiadiazole	802.05 mg	¥2200/1 g (*J&K*)	1764.51
$Pd(PPh_3)_2Cl_2$	73.3 mg	¥45/250 mg (*J&K*)	13.19
$P(OEt)_3$	5 mL	¥50.91/100 mL (Aladdin)	2.54
1-bromo-2-ethylhexane	5.38 g	¥382/100 g (*J&K*)	20.55
KI	4.62 g	¥490.36/100 g (Aladdin)	22.65
K_2CO_3	3.85 g	¥150.1/500 g (*J&K*)	1.16
$POCl_3$	2 mL	¥50.91/50 mL (Aladdin)	2.04
IC-2F	854.34 mg	¥1000/2 g (Derthon)	427.17
pyridine	0.5 mL	¥44.96/100 mL (Aladdin)	0.22
**Total cost (L8-BO, 1g):**		**¥3780.83 ($518.10)**	

Table S21. The MOC evaluation of Z8.

Reagent	Quantity	Unit (source) (By Dec. 2024; www.casmart.com.cn)	Cost (¥)
3-bromothiophene	303.25 mg	¥213/100 g (*J&K*)	0.65
$AlCl_3$	1.25 g	¥41/100 g (*J&K*)	0.51
dodecanoyl chloride	406.89 mg	¥84/50 g (*J&K*)	0.68
K_2CO_3	334.19 mg	¥150.1/500 g (*J&K*)	0.10
ethyl thioglycolate	223.52 mg	¥221/25 g (*J&K*)	1.98
NaOH	223.04 mg	¥35/500 g (*J&K*)	0.02
Cu	1.5 g	¥24/10 g (*J&K*)	3.60
quinolone	80 mL	¥75/100 mL (*J&K*)	60
n-BuLi	0.85 mL	¥443/500 mL (2.4 M, *J&K*)	0.75
Sn(*n*-Bu)$_3$Cl	796.34 mg	¥51.77/25 g (Aladdin)	1.65
**Cost of TT-C$_{11}$-Sn (1g)**		**¥69.94**	
TT-C$_{11}$-Sn	11.86 g	¥69.94/1 g (self-made)	829.49
4,7-dibromo-5,6-dinitrobenzo[*c*][1,2,5]thiadiazole	3.55 g	¥2200/1 g (*J&K*)	7810
$Pd(PPh_3)_2Cl_2$	142.68 mg	¥45/250 mg (*J&K*)	25.68
$P(OEt)_3$	30 mL	¥50.91/100 mL (Aladdin)	15.27
**Cost of Y6-Core (1g)**		**¥11220.11 (11220.76/1.21g)**	
4-phenylbutan-1-ol	6.45 g	¥295/100 g (*J&K*)	19.03
$(CplrCl_2)_2$	684.68 mg	¥953/250 mg (*J&K*)	2610.00
t-BuOK	1.93 g	¥82/100 g (*J&K*)	1.58
PBr_3	13.96 g	¥70/100 g (*J&K*)	9.77
Y6-Core	2.17 g	¥11220.11/1 g (Derthon)	24347.64
KI	240.70 mg	¥490.36/100 g (Aladdin)	1.18
K_2CO_3	2.40 g	¥150.1/500 g (*J&K*)	0.72

n-BuLi	2.5 mL	¥443/500 mL (2.4 M, *J&K*)	2.22
IC-2F	36.41 mg	¥1000/2 g (Derthon)	18.21
pyridine	0.1 mL	¥44.96/100 mL (Aladdin)	0.05
ITN-2F	44.32 mg	¥7000/1 g (Derthon)	310.24
**Total cost (Z8, 1g)**		**¥27320.64 ($3743.83)**	

Table S22. The MOC evaluation of L8-BO-C_4 and L8-BO-C_4-Br.

Reagent	Quantity	Unit (source)<br>(By Dec. 2024; www.casmart.com.cn)	Cost (¥)
5-(bromomethyl) undecane	15.11 g	¥1105/25 g (*J&K*)	667.86
Mg	1.58 g	¥137.5/250 g (*J&K*)	0.87
Oxetane	4.54 g	¥587/5 g (*J&K*)	533.00
CuI	1.15 g	¥269/5 g (*J&K*)	61.87
PCC	3.36 g	¥45/25 g (*J&K*)	6.05
3-bromothieno[3,2-*b*]thiophene	2.49 g	¥842/1 g (TCI)	2096.58747
n-BuLi	4.74 mL	¥443/500 mL (2.4 M, *J&K*)	4.20
$AlCl_3$	1.25 g	¥41/100 g (*J&K*)	0.51
$LiAlH_4$	714.35 mg	¥237.91/25 g (Aladdin)	6.80
n-BuLi	0.73 mL	¥443/500 mL (2.4 M, *J&K*)	0.65
Sn(*n*-Bu)$_3$Cl	781.22 mg	¥51.77/25 g (Aladdin)	1.62
**Cost of TT-BO-C_4-Sn (1g)**		**¥3380**	
TT-BO-Sn	9.15 g	¥439.39/1 g (self-made)	4020.42
4,7-dibromo-5,6-dinitrobenzo[*c*][1,2,5]thiadiazole	5.88 g	¥2200/1 g (*J&K*)	12936
$Pd(PPh_3)_2Cl_2$	214.92 mg	¥45/250 mg (*J&K*)	38.68
TT-BO-C_4-Sn	3.20 g	¥3380/1 g (self-made)	7846.92
Pd_2dba_3	234.42 mg	¥976.74/10 g (Bidepharm)	22.90
P(*o*-tol)$_3$	311.67 mg	¥1047/25 g (*J&K*)	13.05
PPh_3	6.0 g	¥76.42/100 g (Aladdin)	4.59
1-bromo-2-ethylhexane	7.06 g	¥382/100 g (*J&K*)	26.97
KI	6.14 g	¥490.36/100 g (Aladdin)	30.11
K_2CO_3	5.11 g	¥150.1/500 g (*J&K*)	1.53
$POCl_3$	2 mL	¥50.91/50 mL (Aladdin)	2.04

Cost of L8-BO-C_4-CHO (1 g)		¥24943.21	
L8-BO-C_4-CHO	938.66 mg	¥24943.21/1 g (self-made)	23413.19
IC-2F	800.99 mg	¥1000/2 g (Derthon)	400.50
pyridine	1 mL	¥44.96/100 mL (Aladdin)	0.45
**Total cost (L8-BO-C_4, 1g)**		**¥23814.14 ($3263.32)**	
L8-BO-C_4-CHO	815.71 mg	¥24943.21/1 g (self-made)	20346.43
IC-2F	690.51 mg	¥1000/2 g (Derthon)	345.26
pyridine	1 mL	¥44.96/100 mL (Aladdin)	0.45
**Total cost (L8-BO-C_4-Br, 1g)**		**¥20692.14 ($2835.51)**	

Table S23. The MOC evaluation of BTP-C3F.

Reagent	Quantity	Unit (source) (By Dec. 2024; www.casmart.com.cn)	Cost (¥)
hept-6-en-1-yl benzoate	8.31 g	¥289/25 g (*J&K*)	96.06
1,1,1-trifluoro-3-iodopropane	3.22 g	¥50/5 g (*J&K*)	32.2
$Na_2S_2O_2$	17.87 g	¥964/100 g (*J&K*)	172.27
$NaHCO_3$	1.21 g	¥45/500 g (*J&K*)	0.11
Zn	2.29 g	¥25/100 g (*J&K*)	0.57
NaOH	196.94 mg	¥35/500 g (*J&K*)	0.01
PCC	2.28 g	¥45/25 g (*J&K*)	4.10
3-bromothieno[3,2-*b*]thiophene	1.04 g	842/1 g (TCI)	875.68
n-BuLi	1.98 mL	¥443/500 mL (2.4 M, *J&K*)	1.75
$AlCl_3$	656.03 mg	¥41/100 g (*J&K*)	0.27
$LiAlH_4$	334.07 mg	¥237.91/25 g (Aladdin)	3.18
n-BuLi	0.78 mL	¥443/500 mL (2.4 M, *J&K*)	0.69
Sn(*n*-Bu)$_3$Cl	608.57 mg	¥51.77/25 g (Aladdin)	1.26
**Cost of TT-CF$_3$-Sn (1g)**		**¥1188.14**	
TT-CF$_3$-Sn	4.99 g	¥1188.14/1 g (self-made)	5928.82
4,7-dibromo-5,6-dinitrobenzo[*c*][1,2,5]thiadiazole	1.43 g	¥2200/1 g (*J&K*)	3146
$Pd(PPh_3)_2Cl_2$	130.55 mg	¥45/250 mg (*J&K*)	23.50
PPh_3	5.95 mL	¥76.42/100 mL (Aladdin)	4.55
5-(bromomethyl) undecane	5.66 g	¥289/5 g (TCI)	327.15
KI	4.71 g	¥490.36/100 g (Aladdin)	23.10
K_2CO_3	6.27 g	¥150.1/500 g (*J&K*)	1.88
$POCl_3$	0.75 mL	¥50.91/50 mL (Aladdin)	0.76
IC-2F	827.36 mg	¥1000/2 g (Derthon)	413.68

pyridine	1 mL	¥44.96/100 mL (Aladdin)	0.45
**Total cost (BTP-C_3F, 1g)**		**¥9869.89 ($1352.50)**	

Table S24. The MOC evaluation of 3TT-SeS.

Reagent	Quantity	Unit (source)<br>(By Dec. 2024; www.casmart.com.cn)	Cost (¥)
4-ethylaniline	654.37 mg	¥49/25 g (J&K)	1.26
1-bromo-4-ethylbenzene	832.77 mg	¥53/10 g (J&K)	4.41
PdCl2(dppf)	164.63 mg	¥149/1 g (J&K)	24.53
NaOt-Bu	864.9 mg	¥44/25 g (J&K)	1.52
3,6-dibromothieno [3,2-b]thiophene	490.86 mg	¥517/5 g (J&K)	50.75
PdOAc	18.52 mg	¥990/5 g (J&K)	36.67
P(t-Bu)3	66.76 mg	¥254.91/25 g (Aladdin)	6.81
NaOt-Bu	1.04 g	¥60/100 g (J&K)	0.62
NBS	529.22 mg	¥51/100 g (J&K)	0.27
**Cost of TT-2Br (1g)**		**¥126.84**	
TT-2Br	669.62 mg	¥126.84/1 g (self-made)	84.93
TT-SeS-C8-Sn	1.32 g	¥135.22/1 g (self-made)	178.49
$P(t\text{-}Bu)_3Pd$ G3	20.59 mg	¥34/100 mg (*J&K*)	7.00
K_3PO_4	955.21 mg	¥53/100 g (*J&K*)	0.51
$POCl_3$	1 mL	¥50.91/50 mL (Aladdin)	1.02
IC-2F	354.46 mg	¥1000/2 g (Derthon)	177.23
$BF_3{\cdot}Et_2O$	0.5 mL	¥191/100 mL (*J&K*)	0.96
Ac_2O	0.1 mL	¥56.3/500 mL (*J&K*)	0.01
**Total cost (3TT-SeS, 1g)**		**¥450.15 ($61.69)**	

Table S25. The MOC evaluation of 3TTB-ClF.

Reagent	Quantity	Unit (source)<br>(By Dec. 2024; www.casmart.com.cn)	Cost (¥)
3-chloro-4-fluorobenzoic acid	1.53 g	¥282/100 g (*J&K*)	4.31
n-BuLi	9.10 mL	¥443/500 mL (2.4 M, *J&K*)	8.06
Ac_2O	15 mL	¥56.3/500 mL (*J&K*)	16.89
Et_3N	5 mL	¥86/100 mL (*J&K*)	4.30
tert-butyl acetoacetate	1 mL	¥53/25 mL (*J&K*)	2.12
malononitrile	745.16 mg	¥53/25 g (*J&K*)	1.28
NaOAc	925.30 mg	¥290/50 g (*J&K*)	5.37
**Cost of IC-ClF (1g)**		**¥42.33**	
TT-BO-Sn	973.84 mg	¥223.25/1 g (self-made)	217.41
TT-2Br	724 mg	¥42.33/1 g (self-made)	30.65
P(*t*-Bu)$_3$Pd G3	22.19 mg	¥34/100 mg (*J&K*)	7.54
K_3PO_4	1.03 g	¥53/100 g (*J&K*)	0.55
$POCl_3$	2 mL	¥50.91/50 mL (Aladdin)	2.04
IC-2F	369.65 mg	¥1000/2 g (Derthon)	184.83
$BF_3 \cdot Et_2O$	0.5 mL	¥191/100 mL (*J&K*)	0.96
Ac_2O	0.1 mL	¥56.3/500 mL (*J&K*)	0.01
**Total cost (3TTB-ClF, 1g)**		**¥443.99 ($60.84)**	

Table S26. Photovoltaic parameters and E_{loss} for the NFREA-based OSCs with over 15% efficiency in literatures.

Active layers	V_{oc} (V)	J_{sc} (mA cm^{-2})	FF (%)	PCE (%)	E_{loss} (eV)	Refs.
JD40:R4T-1	0.80	25.48	74.52	15.10	0.619	[37]
D18:DPA-5	0.924	22.54	72.58	15.12	0.556	[36]
PBDB-TF:A4T-16	0.876	21.8	79.8	15.2	0.634	[38]
D18:TT-S-2F	0.83	24.23	76.10	15.29	0.66	[39]
D18:HIOTT-4F	0.93	23.1	71.7	15.4	0.572	[40]
D18:3TT-C2	0.90	22.31	76.47	15.42	0.572	[41]
D18:2BTh-2F(C2)	0.90	23.61	72.30	15.44	0.67	[20]
D18:3TTB-FCl	0.945	22.95	71.53	15.51	0.536	[34]
D18:3TTS-4F	0.917	24.17	71.58	15.86	0.545	[33]
PBQx-TF:TBT-13	0.79	25.9	77.9	16.1	0.611	[42]
D18:BM-2F	0.91	24.21	73.35	16.15	0.58	[43]
D18:3TT-C2-Cl	0.89	23.14	77.91	16.17	0.606	[41]
D18:CH3-OC4	0.84	26.00	75.27	16.44	0.64	[44]
D18:OC4-4Cl-C8	0.90	24.40	75.10	16.56	0.56	[45]
D18:DPA-4	0.924	24.41	73.85	16.67	0.541	[36]
PQSe-TCl:A4T-16	0.885	24.0	79.7	16.9	0.627	[46]
PBQx-TF:TBT-26	0.808	26.1	80.7	17.0	0.608	[47]
D18:3TT-C2-F	0.90	24.13	78.75	17.19	0.598	[41]
D18:3TTB-4F	0.947	24.83	73.91	17.38	0.536	[33]
D18:3TTB-ClF	0.971	24.45	73.56	17.46	0.529	[34]
D18:FIOTT-4F	0.92	24.0	79.1	17.5	0.601	[40]
D18:412-6F	0.897	25.83	77.83	18.03	0.61	[48]

Table S27. Detailed parameters of energy loss in photovoltaic devices.

Devices	E_g (eV)	EQE_{EL}	E_{CT} (eV)	ΔE_{CT} (eV)	ΔE_r (eV)	ΔE_{nr} (eV)	E_{loss} (eV)
D18:3TT-SSe	1.428	2.16×10^{-4}	1.364	0.064	0.253	0.211	0.528
D18:3TT-SeS	1.444	2.87×10^{-4}	1.372	0.072	0.243	0.204	0.519

Table S28. Detailed GIWAXS data of the D18:3TT-SSe and D18:3TT-SeS blend films.

	OOP			
	Location (Å^{-1})	*d*-spacing (Å)	CCL (Å)	*g*-factor
D18:3TT-SSe	1.66	3.79	13.96	0.208
D18:3TT-SeS	1.68	3.74	21.67	0.166

5 Supplementary References

1. Frisch MJ, Trucks GW, Schlegel HB *et al.* Gaussian 16 Rev. B.01. *Wallingford CT* 2016.

2. Lu T, Chen F. Multiwfn: A multifunctional wavefunction analyzer. *J Comput Chem* 2012; **33**: 580–592.

3. Tian L. Molclus Program, Version 1.9.9.9. http://www.keinsci.com/research/molclus.html (6 March 2022, date last accessed).

4. *Semiempirical Extended Tight-Binding Program Package xtb*. (2022-07-20)

5. Bannwarth C, Caldeweyher E, Ehlert S *et al.* Extended tight-binding quantum chemistry methods. *Wiley Interdiscip Rev:Comput Mol Sci* 2021; **11**: e1493.

6. Frisch MJ, Trucks GW, Schlegel HB *et al.* Gaussian 16 Rev. B.01. Wallingford, CT; 2016.

7. Neese F. Software update: The ORCA program system-Version 5.0. *Wiley Interdiscip Rev:Comput Mol Sci* 2022; **12**: e1606.

8. Grimme S, Bannwarth C, Shushkov P. A Robust and Accurate Tight-Binding Quantum Chemical Method for Structures, Vibrational Frequencies, and Noncovalent Interactions of Large Molecular Systems Parametrized for All spd-Block Elements (Z = 1–86). *J Chem Theory Comput* 2017; **13**: 1989–2009.

9. Stephens PJ, Devlin FJ, Chabalowski CF *et al.* Ab Initio Calculation of Vibrational Absorption and Circular Dichroism Spectra Using Density Functional Force Fields. *J Phys Chem* 1994; **98**: 11623-11627.

10. Grimme S. Density functional theory with London dispersion corrections. *Wiley Interdiscip Rev:Comput Mol Sci* 2011; **1**: 211–228.

11. Grimme S, Ehrlich S, Goerigk L. Effect of the damping function in dispersion corrected density functional theory. *J Comput Chem* 2011; **32**: 1456–1465.

12. Weigend F, Ahlrichs R. Balanced basis sets of split valence, triple zeta valence and quadruple zeta valence quality for H to Rn: Design and assessment of accuracy. *Phys Chem Chem Phys* 2005; **7**: 3297–3305.

13. Weigend F. Accurate Coulomb-fitting basis sets for H to Rn. *Phys Chem Chem Phys* 2006; **8**: 1057–1065.

14. Zhao Y, Truhlar DG. The M06 suite of density functionals for main group thermochemistry, thermochemical kinetics, noncovalent interactions, excited states, and transition elements: two new functionals and systematic testing of four M06-class functionals and 12 other functionals. *Theor Chem Account* 2008; **120**: 215–241.

15. Lu T, Chen Q. Shermo: A general code for calculating molecular thermochemistry properties. *Comput Theor Chem* 2021; **1200**: 113249.

16. Humphrey W, Dalke A, Schulten K. VMD: Visual molecular dynamics. *J Mol Graph Model* 1996; **14**: 33–38.

17. Kyaw AKK, Wang DH, Wynands D *et al.* Improved Light Harvesting and Improved Efficiency by Insertion of an Optical Spacer (ZnO) in Solution-Processed Small-Molecule Solar Cells. *Nano Lett* 2013; **13**: 3796–3801.

18. Shen Y, Hosseini AR, Wong MH *et al.* How To Make Ohmic Contacts to Organic Semiconductors. *ChemPhysChem* 2004; **5**: 16–25.

19. Gu X, Wei Y, Zeng R *et al.* Suppressing Exciton–Vibration Coupling via Intramolecular Noncovalent Interactions for Low-Energy-Loss Organic Solar Cells. *Angew Chem Int Ed* 2025; **64**: e202418926.

20. Wang X, Lu H, Liu Y *et al.* Simple Nonfused Ring Electron Acceptors with 3D Network Packing Structure Boosting the Efficiency of Organic Solar Cells to 15.44%. *Adv Energy Mater* 2021; **11**: 2102591.

21. Zeng R, Zhang M, Wang X *et al.* Achieving 19% efficiency in non-fused ring electron acceptor solar cells via solubility control of donor and acceptor crystallization. *Nat Energy* 2024; **9**: 1117–1128.

22. Zhang M, Guo X, Zhang S *et al.* Synergistic Effect of Fluorination on Molecular Energy Level Modulation in Highly Efficient Photovoltaic Polymers. *Adv Mater* 2014; **26**: 1118–1123.
23. Zhong X, Chen T-W, Yan L *et al.* Facile Synthesis of Key Building Blocks of D18 Series Conjugated Polymers for High-Performance Polymer Solar Cells. *ACS Appl Polym Mater* 2023; **5**: 1937–1944.
24. Liu Q, Jiang Y, Jin K *et al.* 18% Efficiency organic solar cells. *Sci Bull* 2020; **65**: 272–275.
25. Yao H, Ye L, Zhang H *et al.* Molecular Design of Benzodithiophene-Based Organic Photovoltaic Materials. *Chem Rev* 2016; **116**: 7397–7457.
26. Qin J, Zhang L, Zuo C *et al.* A chlorinated copolymer donor demonstrates a 18.13% power conversion efficiency. *J Semicond* 2021; **42**: 010501.
27. Chao P, Mu Z, Wang H *et al.* Chlorination of Side Chains: A Strategy for Achieving a High Open Circuit Voltage Over 1.0 V in Benzo[1,2-b:4,5-b′]dithiophene-Based Non-Fullerene Solar Cells. *ACS Appl Energy Mater* 2018; **1**: 2365‑2372.
28. Li C, Zhou J, Song J *et al.* Non-fullerene acceptors with branched side chains and improved molecular packing to exceed 18% efficiency in organic solar cells. *Nat Energy* 2021; **6**: 605–613.
29. Jiang Y, Sun S, Xu R *et al.* Non-fullerene acceptor with asymmetric structure and phenyl-substituted alkyl side chain for 20.2% efficiency organic solar cells. *Nat Energy* 2024; **9**: 975–986.
30. Jiang K, Wei Q, Lai JYL *et al.* Alkyl Chain Tuning of Small Molecule Acceptors for Efficient Organic Solar Cells. *Joule* 2019; **3**: 3020–3033.
31. Li C, Song J, Lai H *et al.* Non-fullerene acceptors with high crystallinity and photoluminescence quantum yield enable >20% efficiency organic solar cells. *Nat Mater* 2025; **24**: 433–443.
32. Dong J, Li Y, Liao C *et al.* Dielectric Constant Engineering of Nonfullerene Acceptors Enables a Record Fill Factor of 83.58% and a High Efficiency of 20.80% in Organic Solar Cells. *Energy Environ Sci* 2025; **18**: 4982–4995.
33. Gu X, Zeng R, He T *et al.* Simple-Structured Acceptor with Highly Interconnected Electron-Transport Pathway Enables High-Efficiency Organic Solar Cells. *Adv Mater* 2024; **36**: 2401370.
34. Gu X, Zeng R, Hou Y *et al.* Precisely Regulating Intermolecular Interactions and Molecular Packing of Nonfused-Ring Electron Acceptors via Halogen Transposition for High-Performance Organic Solar Cells. *Angew Chem Int Ed* 2024; **63**: e202407355.
35. Ma D-L, Zhang Q-Q, Li C-Z. Unsymmetrically Chlorinated Non-Fused Electron Acceptor Leads to High-Efficiency and Stable Organic Solar Cells. *Angew Chem Int Ed* 2023; **62**: e202214931.
36. Han Z, Zhang Ce, He T *et al.* Precisely Manipulating Molecular Packing via Tuning Alkyl Side-Chain Topology Enabling High-Performance Nonfused-Ring Electron Acceptors. *Angew Chem Int Ed* 2024; **63**: e202318143.
37. Shen S, Mi Y, Ouyang Y *et al.* Macrocyclic Encapsulation in a Non-fused Tetrathiophene Acceptor for Efficient Organic Solar Cells with High Short-Circuit Current Density. *Angew Chem Int Ed* 2023; **62**: e202316495.
38. Ma L, Zhang S, Zhu J *et al.* Completely non-fused electron acceptor with 3D-interpenetrated crystalline structure enables efficient and stable organic solar cell. *Nat Commun* 2021; **12**: 5093.
39. Zheng X, Liu W, Wang H *et al.* Simple non-fused ring electron acceptors with well-controlled terminal group stacking. *Cell Rep Phys Sci* 2022; **3**: 101169.
40. Ye S, Chen T, Yu J *et al.* Enhanced crystal network and charge transfer of non-fused ring electron acceptors via interchain interaction for efficient and stable organic solar cells. *Energy Environ Sci* 2024; **17**: 5137–5146.
41. Li D, Zhang H, Cui X *et al.* Halogenated Nonfused Ring Electron Acceptor for Organic Solar Cells with a Record Efficiency of Over 17%. *Adv Mater* 2024; **36**: 2310362.
42. Yang N, Cui Y, Zhang T *et al.* Molecular Design of Fully Nonfused Acceptors for Efficient Organic Photovoltaic Cells. *J Am Chem Soc* 2024; **146**: 9205–9215.

43. Wang X, Zeng R, Lu H *et al.* A Simple Nonfused Ring Electron Acceptor with a Power Conversion Efficiency Over 16%. *Chin J Chem* 2023; **41**: 665–671.

44. Zheng X, Wei N, Liu W *et al.* Side Chain Enabling High-Performance Nonfused Ring Electron Acceptor with Controlled Aggregation Behavior. *CCS Chem* 2025; **7**: 2215–2224.

45. Zheng X, Liu W, Wei N *et al.* Designing high-performance nonfused ring electron acceptors via side-chain engineering. *Aggregate* 2024; **5**: e469.

46. Xiao Y, Yao H, Wang J *et al.* A Selenium-Containing Polymer Donor Enables 16.9% Efficiency in Non-Fused Electron Acceptor-Based Organic Photovoltaic Cells. *Adv Energy Mater* 2024; **14**: 2400928.

47. Yang N, Cui Y, Xiao Y *et al.* Completely Non-fused Low-cost Acceptor Enables Organic Photovoltaic Cells with 17% Efficiency. *Angew Chem Int Ed* 2024; **63**: e202403753.

48. Zheng X, Jiang X, Liu W *et al.* Boosting Organic Solar Cells to Over 18% Efficiency through Dipole-Dipole Interactions in Fluorinated Nonfused Ring Electron Acceptors. *Angew Chem Int Ed* 2024; **63**: e202412854.
